# Comparative study of Hippo pathway genes in cellular conveyor belts of a ctenophore and a cnidarian

**DOI:** 10.1186/s13227-016-0041-y

**Published:** 2016-02-19

**Authors:** Alicia Coste, Muriel Jager, Jean-Philippe Chambon, Michaël Manuel

**Affiliations:** Sorbonne Universités, Université Pierre et Marie Curie (UPMC), Institut de Biologie Paris-Seine (IBPS) CNRS, UMR 7138 Evolution Paris-Seine, Case 05, 7 quai St Bernard, 75005 Paris, France

**Keywords:** Cell proliferation, Cnidaria, Ctenophora, Development, Evolution, Growth, Hippo pathway, Yorkie

## Abstract

**Background:**

The Hippo pathway regulates growth rate and organ size in fly and mouse, notably through control of cell proliferation. Molecular interactions at the heart of this pathway are known to have originated in the unicellular ancestry of metazoans. They notably involve a cascade of phosphorylations triggered by the kinase Hippo, with subsequent nuclear to cytoplasmic shift of Yorkie localisation, preventing its binding to the transcription factor Scalloped, thereby silencing proliferation genes. There are few comparative expression data of Hippo pathway genes in non-model animal species and notably none in non-bilaterian phyla.

**Results:**

All core Hippo pathway genes could be retrieved from the ctenophore *Pleurobrachia pileus* and the hydrozoan cnidarian *Clytia hemisphaerica*, with the important exception of Yorkie in ctenophore. Expression study of the Hippo, Salvador and Scalloped genes in tentacle “cellular conveyor belts” of these two organisms revealed striking differences. In *P. pileus*, their transcripts were detected in areas where undifferentiated progenitors intensely proliferate and where expression of cyclins B and D was also seen. In *C. hemisphaerica*, these three genes and Yorkie are expressed not only in the proliferating but also in the differentiation zone of the tentacle bulb and in mature tentacle cells. However, using an antibody designed against the *C. hemiphaerica* Yorkie protein, we show in two distinct cell lineages of the medusa that Yorkie localisation is predominantly nuclear in areas of active cell proliferation and mainly cytoplasmic elsewhere.

**Conclusions:**

This is the first evidence of nucleocytoplasmic Yorkie shift in association with the arrest of cell proliferation in a cnidarian, strongly evoking the cell division-promoting role of this protein and its inhibition by the activated Hippo pathway in bilaterian models. Our results furthermore highlight important differences in terms of deployment and regulation of Hippo pathway genes between cnidarians and ctenophores.

**Electronic supplementary material:**

The online version of this article (doi:10.1186/s13227-016-0041-y) contains supplementary material, which is available to authorized users.

## Background

In the context of a multicellular organism, basic cellular processes such as cell proliferation, differentiation and apoptosis have to be finely tuned in time and space to ensure correct and coordinated cell turnover, growth rate and size at the tissue, organ and organism levels. In recent years, the Hippo pathway has emerged as a major actor of this orchestration [1–6]. Functional studies in fly have revealed that the kinase Hippo (Hpo) negatively regulates cell proliferation and organ size and promotes apoptosis and inversely that the downstream transcriptional co-activator Yorkie (Yki) enhances cell proliferation rates and organ size whilst limiting apoptosis [2–4, 7–9]. It was later discovered that orthologues of these and other components of the Hippo pathway have essentially similar functions in mammals in a wide variety of developmental stages and tissues [1, 4, 10–12]. This prompted the suggestion that the Hippo pathway has a conserved role at the phylogenetic level of Bilateria in negative regulation of tissue and organ size, through inhibition of Yki notably leading to cell cycle exit. These effects are triggered in response to multiple cues from the cell environment and in the context of an intense crosstalk with other key signalling pathways such as Wnt, TGF-β and notch [12–14].

A critical aspect of the regulatory logic of the Hippo pathway is modulation of the behaviour of the Yki protein (in mammals represented by two paralogues called YAP and TAZ). In the inactive pathway state, this protein translocates to the cell nucleus where it can bind to a variety of transcription factors to affect gene transcription. Effects on cell proliferation are principally mediated through Yki binding to Scalloped (Sd) (in mammals, TEAD transcription factors) [2, 4, 15–18]. Activated Sd enhances the transcription of genes driving stemness and cell division [5] (examples of identified target genes include *CyclinE*, *Myc*, *FoxO*, *e2f1*, *wingless* [19]). When the Hippo pathway is activated, the kinase Hpo (in mammals, MST) together with its adaptor protein Salvador (Sav) (in mammals, SAV, also called WW45) phosphorylates the complex formed by Warts (Wts) (in mammals, LATS) and Mats (in mammals, MOB1) [20–22]. Then, Warts phosphorylates Yki at a critical serine residue (S127 in human YAP) [2, 4, 23–25]. As a consequence, Yki is sequestered in the cytoplasm, notably through interaction with the 14-3-3 protein [26, 27]. Not only this prevents Yki from activating Sd in the nucleus (thus down-regulating genes associated with stem cells and cell division), but also it allows Yki to exert specific functions in the cytoplasm, including effects on cell fate and differentiation, documented in a number of systems [12, 28–31]. These core components of the Hippo pathway stand at the node of a complex network of interactions [5, 12]. Regulatory inputs upstream of Hpo are multiple and have only started to be unveiled (reviewed in [2, 4, 12]). In addition, there are Yki-independent effects of Hpo, as well as Hpo-independent regulations of Yki [12], further illustrating the complexity hiding behind what we call the “Hippo pathway”.

The Hippo pathway is evolutionarily conserved far beyond the clade of bilaterian animals. Comparative genomic studies have identified orthologues of most Hippo pathway members in non-bilaterian animal phyla (cnidarians, placozoans, sponges) as well as in the closest unicellular relatives of metazoans: choanoflagellates (e.g. *Monosiga brevicollis*) and ichthyosporids (e.g. *Capsaspora owczarzaki*) [32–34]. According to the analyses performed by Sebé-Pedrós et al. [34], the core components originated sequentially in this order: Mats in a remote eukaryote ancestor; Hpo and Sd in a common ancestor of unikonts (amoebozoans, fungi, metazoans and their kins); Warts in an ancestor of opisthokonts (the least inclusive clade containing fungi and animals); Yki in an exclusive ancestor of holozoans (*Capsaspora*, choanoflagellates and metazoans); Sav after the divergence of *Capsaspora*. The same study found evidence for secondary losses of Hpo in non-chytrid fungi and the choanoflagellate *Monosiga* (Hpo present in the other choanoflagellate *Salpingoeca*) and of Sd in chytrids.

Furthermore, recent experimental data suggest that the growth regulatory function of Hippo pathway genes has its roots in a common ancestor of holozoans. Thus, co-expression of the *Capsaspora* versions of Yki and Sd led to significant eye tissue overgrowth in *Drosophila melanogaster*, and conversely overexpression of *Capsaspora* Hpo resulted in smaller eyes [34]. Similar phenotypes were obtained from heterologous expression in fly eye imaginal discs of the Yki genes from the placozoan *Trichoplax adhaerens* and the cnidarian *Nematostella vectensis* [19]. It was furthermore demonstrated that the Yki and Sd proteins of *Capsaspora* bind together, and that *Capsaspora* Hpo induces phosphorylation of fly and *Capsaspora* Yorkies in cell cultures and of fly Yki in vivo. The molecular regulation mechanisms at the heart the Hpo pathway therefore clearly emerged before the evolutionary origin of multicellular animals.

However, there is still a critical lack of data in non-model metazoans on the expression and function of core Hippo pathway components, as well as on the subcellular localisation of Yki in relation to the proliferative capacities of cells. Apart from fly and mammals, the Hippo pathway has been substantially investigated only in flatworms (Platyhelminthes), with contradictory conclusions as to the conservation of the role of Yki, depending on the species studied. In *Macrostomum lignano*, Yki promotes cell proliferation and growth, like in *Drosophila* and mouse [35], but in the other flatworm *Schmidtea mediterranea*, Yorkie is ubiquitously expressed, has a restrictive influence on cell proliferation and is involved in morphogenesis of the excretory system [36]. Since flatworms are thought to be particularly important to understanding the early evolution of stem cell regulation mechanisms in bilaterians, this contrasted picture precludes firm conclusions concerning the extent of conservation of Yki and Hpo pathway functions. At a higher evolutionary level, up to now orthologues of Hpo pathway genes from animal lineages that diverged before the bilaterians have been studied only in the context of heterologous expression in fly.

In this contribution, we present the first expression study of core Hpo pathway genes in two non-bilaterian phyla, ctenophores and cnidarians, and the first data on Yki subcellular localisation in relation to cell proliferation in a cnidarian. Ctenophores and cnidarians are traditionally thought to be phylogenetically closer to the Bilateria than sponges and placozoans, notably because they possess nerve cells, muscle cells and a gut (synapomorphies of Eumetazoa). Recently, phylogenomic studies have confirmed a close relationships between cnidarians and bilaterians, but many of these analyses have come up with a highly unconventional placement of ctenophores as sister group to all other metazoans (including sponges and placozoans) [37–40]. The position of ctenophores is currently hotly debated, given that other phylogenomic studies suggest that an artefact known as “long-branch attraction” is in fact responsible for this “basal” placement [41–44].

Irrespective of this phylogenetic controversy, the two species investigated here, the ctenophore *Pleurobrachia pileus* and the hydrozoan medusa *C. hemisphaerica*, are of special interest to addressing molecular aspects of cell proliferation and differentiation in non-bilaterian animals, owing to the existence of “cellular conveyor belts” [45] in the proximal part of their tentacles. Cellular conveyor belts are cell renewal systems in which stem cells are spatially restricted (at one extremity of the belt), and cellular stages of proliferation, cell cycle exit and the successive steps of differentiation are spatially ordered in a sequence that recapitulates their timely succession. A seminal example in bilaterians is the vertebrate intestinal crypt [45]. Such systems offer a decisive advantage in organisms for which advanced techniques of cellular biology and gene functional studies are not available, because the association of a given gene expression pattern, or any other cell feature, with a given cell state (from stem cells to mature differentiated cells) can be deduced from the cell position along the conveyor belt [45].

**Fig. 1:**
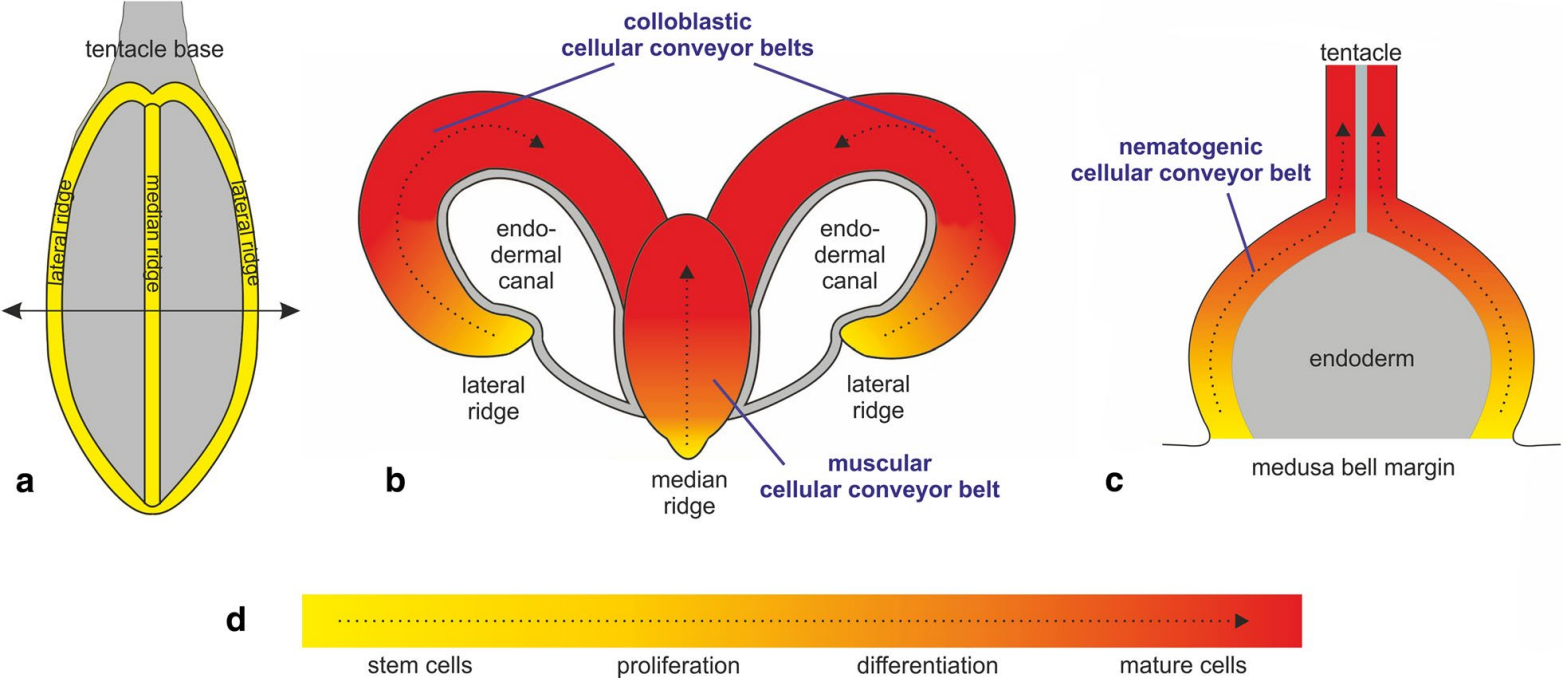
Schematic representation of *P. pileus* and *C. hemisphaerica* cellular conveyor belts involved in continuous regeneration of tentacle tissues. **a** Aspect of a *P. pileus* tentacle root extracted from the animal and viewed from the internal side. The lateral and median ridges (housing stem cells) are highlighted in *yellow* (rest of tentacle root surface in *grey*). **b** Transverse section of the *P. pileus* tentacle root according to the sectioning plane shown in (**a**; *double arrow*) (endoderm in *grey*). The epithelium of the tentacle sheath that partially surrounds the tentacle root is not represented. **c** Longitudinal section of a *C. hemisphaerica* tentacle bulb and its tentacle (endoderm in *grey*). In **b**, **c** the *dotted arrowed lines* materialise progression along cell lineages and correlative cell movements. **d** Legend of *colour scale* used in **a**–**c** in terms of dominant cellular stages, according to data published in [46, 49, 50]. Note that cellular conveyor belts are not organised into well-defined compartments (except perhaps for the stem cell niche) but instead form a more or less continuous gradient in terms of the relative abundance of cellular stages

Cellular conveyor belts housed in proximal swellings of *P. pileus* and *C. hemisphaerica* tentacles are involved in their constant and intense regeneration needed to compensate tentacle injury during feeding. They have been previously characterised at the cellular and molecular level notably through electron microscopy study, monitoring of proliferating nuclei after BrdU or EdU incorporation, and expression analyses of stem cell and differentiation genes [46–51]. In the *P. pileus* tentacle root (Fig. 1a, b), there is a central conveyor belt that provides tentacle mesogleal cells (muscle cells, and probably also other cell types including neurons) and two symmetrical lateral conveyor belts mainly involved in the production of colloblasts, the ctenophore-specific adhesive cells used to catch preys (main cell type of the tentacle ectoderm). Each one of these three conveyor belts corresponds to a flattened expansion visible in transverse section on the internal side (i.e. the side facing the body axis) of the tentacle root, with the stem cells concentrated along a ridge forming the distal margin of the expansion (yellow areas in Fig. 1a, b). The *C. hemisphaerica* tentacle bulb is more simply organised, with a single conveyor belt in the ectoderm, oriented from proximal to distal (Fig. 1c). The stem cells are located close to the insertion point of the bulb on the umbrella periphery (yellow zone in Fig. 1c). This cellular conveyor belt is thought to provide all ectodermal cells of the tentacle, notably the cnidarian-specific stinging cells or nematocytes.

In this study, we first looked for the orthologues in cnidarians and ctenophores of core Hpo pathway genes, through Blast searches and phylogenetic analyses. Whereas all searched genes could be identified in cnidarians, a *bona fide* Yorkie orthologue is apparently absent from ctenophore genomes despite presence of the other core Hpo pathway genes. Then, we analysed the expression of cyclins B and D (as markers of cell proliferation) and of the core Hpo pathway genes Hpo, Sav and Sd in the tentacle root of *P. pileus* and Hpo, Sav, Yki and Sd in the tentacle bulb of *C. hemisphaerica*. Significant differences in transcript distribution of orthologous genes were observed between the cellular conveyor belts of these two organisms. Using an antibody specifically designed to recognise *C. hemisphaerica* Yorkie, associated with EdU labelling of proliferating nuclei, we finally show that the subcellular localisation of this protein varies between cells (from entirely nuclear to entirely cytoplasmic) and that there is strong correlation across the medusa tissues between areas of predominantly nuclear Yki localisation and of intense cell proliferation.

## Methods

### Animal collection and fixation

Adult specimens of *P. pileus* were collected using plankton nets in Roscoff and in Gravelines (France) during their reproductive season, between March and June. The field work done in the context of this work did not involve any endangered or protected species and did not require any specific permission. Furthermore, no ethical approval was required for the animal experiments done in this study. Living adult *P. pileus* specimens were transferred into filtered natural seawater and kept in the laboratory at 16 °C. For in situ hybridisation, after 24 h of starvation, they were fixed at room temperature in 4 % paraformaldehyde in 50 % seawater and 50 % PBT (10 mM Na_2_HPO4, 150 mM NaCl, pH 7.5, 0.1 % Tween 20) for 60 min. Fixation for immunofluorescence was done at 4 °C for 30 min. Then, specimens were washed three times in PBT and dehydrated through a graded series of ethanol and stored in methanol at −20 °C. Medusae of *C. hemisphaerica* were obtained in our laboratory by culture of polyp colonies established since several years from strains initially provided by Evelyn Houliston (Villefranche-sur-mer) and raised as previously described [52], except that artificial seawater (Red Sea^®^) was used. Medusae were left unfed during 24 h before fixation. For in situ hybridisation, medusae were fixed during 40 min at 4 °C in 3.7 % formaldehyde, 0.2 % glutaraldehyde in PBT. For immunofluorescence, they were fixed for 30 min at room temperature in paraformaldehyde 4 % in PBT. After fixation, medusae were dehydrated as described above.

### In situ hybridisation

The in situ hybridisation (ISH) protocol for *P. pileus* was as described in [50]. Following whole-mount ISH, specimens were microdissected. Tentacle roots were extracted from the body for separate observation. Transverse cryosectioning of tentacle roots after whole-mount ISH was performed using a Leica CM1860 cryostat at a thickness of 14 µm, as described in [49]. In situ hybridisation of *C. hemisphaerica* medusae was performed as in [47]. After post-fixation and Dapi staining, samples were mounted in Citifluor^®^. All DIC images were obtained with an Olympus BX61 microscope using a Q-imaging Camera with Image Pro plus^®^ software (MediaCybernetics).

### Gene phylogenies

Data sets were built by blasting (BlastP) human proteins on NCBI against predicted proteins from the complete genomes of *C. owczarzaki* (Ichthyosporea), *M. brevicollis* (Choanoflagellata), *Amphimedon queenslandica* (Porifera, Demospongiae), *Sycon ciliatum* (Porifera, Calcarea) *T. adhaerens* (Placozoa), *N. vectensis* (Cnidaria, Anthozoa), *Hydra magnipapillata* (Cnidaria, Hydrozoa), *Lottia gigantea* (Lophotrochozoa, Mollusca), *D. melanogaster* (Ecdysozoa, Arthropoda), *Strongylocentrotus purpuratus* (Deuterostomia, Echinodermata), *Branchiostoma floridae* (Deuterostomia, Chordata, Cephalochordata), *Ciona intestinalis* (Deuterostomia, Chordata, Urochordata), *Danio rerio* (Deuterostomia, Chordata, Vertebrata, Teleostea), *Xenopus tropicalis* (Deuterostomia, Chordata, Vertebrata, Amphibia) and *Homo sapiens*. In addition, Blast (tBlastN) searches with the same query sequences were performed on the full genome of the ctenophore *Mnemiopsis leidyi* and our transcriptome assembly of the other ctenophore *P. pileus* and on the current transcriptome assembly of the hydrozoan cnidarian *C. hemisphaerica*. In the case of Yorkie, since no orthologue was found in the *M. leidyi* genome and the *P. pileus* transcriptome (see “Results”), the genome of *Pleurobrachia bachei* was blasted as well.

For these Blast searches, an *e* value threshold was determined empirically for each protein family, after blasting the human protein against the human genome, in order to retrieve not only the orthology group of interest but also at least one additional orthology group of the same multigenic family, in order to root the tree. Thus, closest orthology groups used for rooting were Nedd4 for Yorkie, membrane-associated guanylate kinase WW and PDZ domain-containing protein (MAGI) for Salvador, MOB kinase activator 3 for Mats and serine–threonine kinase 38 for Warts. In the alignment built for analysis of Hippo, a more extended sampling of several subfamilies of kinases close to Hippo was selected as outgroup (from the genome of *Branchiostoma floridae*). Because Scalloped is an isolated family of transcription factors (no paralogy above phylum level), in this case the tree was rooted taxonomically using sequences from *C. owczarzaki* and *M. brevicollis*.

Since Yorkie and Salvador both contain multiple WW domains, for these two gene families, we first built an alignment of WW domains only, in order to determine their orthologies (i.e. which WW domain of a given gene in a given species corresponds to which WW domain in other genes). When several WW domains were present in the same protein, they were labelled alphabetically according to their position, starting from the N-terminal extremity (i.e. for Yorkie: Yki a and Yki b correspond to the first and second WW domains, respectively; for Nedd4, WW domains labelled from a to e). Results of WW domain analyses were taken into account to build combined alignments; for Yorkie, of the TEAD-binding (TBD) domain and the two WW domains (TBD domain replaced with missing data for Nedd4 proteins); for Salvador, of the SARAH domain and the two WW domains.

Alignments were constructed automatically using MUSCLE [53]. They were visually checked, and ambiguously aligned parts were removed in BioEdit [54]. Alignments used to build the trees (in FASTA format) are provided as Additional file 1. Phylogenetic analyses were carried out from the amino-acid alignments by maximum likelihood (ML) using the PhyML 3.1 program [55] with the LG model of amino-acid substitution and a BioNJ tree as the input tree. A gamma distribution with four categories was used, and the gamma shape parameter and the proportion of invariant sites were optimised during the searches. Statistical robustness was estimated by computation of bootstrap values (100 replicates).

### Production and validation by SDS-PAGE and Western blot of an anti-CheYki antibody

A polyclonal antibody against *C. hemisphaerica* Yorkie (=antiserum) was produced by Eurogentec (Speedy 28-day polyclonal packages) in rabbit against the peptide H–CFNRRTTWDDPRKAHS–NH2. The antiserum and negative controls were tested by Western blot. For preparation of a protein extract, medusae were sonicated on ice in RIPA lysis buffer (150 mM NaCl, 50 mM Tris–Cl, pH 7.6, 5 mM EDTA, 0.5 % NP-40, 1 mM β-glycerophosphate, 1 mM orthovanadate, 0.1 % SDS) supplemented with Complete Protease Inhibitor Cocktail Tablets (Roche Molecular Biochemicals). Lysates were then clarified by centrifugation. Samples were diluted in sample buffer [56] and incubated at 95 °C for 5 min. Three equivalent aliquots of total proteins were separated on a 12.5 % SDS gel and transferred onto a nitrocellulose membrane (stained with Ponceau red to check for presence of proteins). The membrane was then cut in three pieces corresponding to the three protein aliquots. They were blocked with 5 % BSA in TBS-Tween (Tris 50 mM, NaCl 150 mM, pH 7.6, 0.1 % Tween 20). Then, piece 1 was incubated with the anti-CheYki rabbit antiserum diluted 1/250 in TBS-Tween; piece 2 with the same antiserum that was previously incubated with the immunising peptide (3.4 mM); piece 3 with rabbit pre-immune serum diluted 1/250 in TBS-Tween (all these incubations overnight at 4 °C). Then, the blots were washed in TBS-Tween, incubated for 1 h with the secondary antibody (HRP-conjugated anti-rabbit IgG antibody, diluted 1/30,000), then washed in TBS-Tween. Labelled proteins were detected using the ECL chemiluminescence kit (Amersham Pharmacia Biotech). Results of these validation experiments are presented in Additional file 2.

### Immunocyto-localisation

Stepwise rehydration of fixed medusae was performed using PBS (10 mM Na_2_HPO4, 150 mM NaCl, pH 7.5) + Triton-X100 0.01 %, followed by sample permeabilisation (Triton-X100 0.2 % in PBS, then 0.01 % in PBS, 10 min at room temperature). After blocking with 1 % bovine serum albumin (BSA), samples were incubated for 4 h at room temperature or overnight at 4 °C with the anti-CheYki antibody (1/100 dilution). After being washed with PBS + Triton-X100 0.01 %, samples were incubated overnight at 4 °C with the secondary antibody Alexa Fluor ^®^ 568 goat anti-rabbit IgG (1/2000 dilution, Molecular probes). Samples were finally stained with Dapi (1 µg/ml) for 15 min for DNA visualisation, washed three times for 15 min in PBS + Triton-X100 0.01 % and mounted on slides for microscope observation. Fluorescence labelling was monitored with a confocal microscope (Leica SP5), and images were acquired using Leica LAS-AF software. Negative controls were performed with pre-immune rabbit serum and with the secondary antibody only (see Additional file 3).

### EdU labelling of proliferating nuclei

EdU incorporation assays were done using the Click-iT^®^ EdU Alexa Fluor^®^ 488 Imaging Kit from Invitrogen (Durham, NC, USA). Medusae were incubated in artificial sea water containing 100 μM EdU, for 2 h; then, they were immediately fixed in PFA 4 %, 50 % sea water, 50 % PBS + Triton-X100 0.01 %. After fixation for 30 min at room temperature, the samples were blocked with 3 % BSA in PBS + Triton-X100 0.01 % and then permeabilised in PBS triton X100 0.5 % for 20 min. After washing with 3 % BSA in PBS + Triton-X100 0.01 %, animals were incubated in the EdU detection solution as indicated by the manufacturer. All samples were labelled with anti-CheYki antibody (as described in previous paragraph) and finally stained with Dapi and mounted as described for ISH experiments.

## Results

### The ctenophore repertoire of Hippo pathway genes and cyclins, and other lessons from the gene phylogenies

**Fig. 2:**
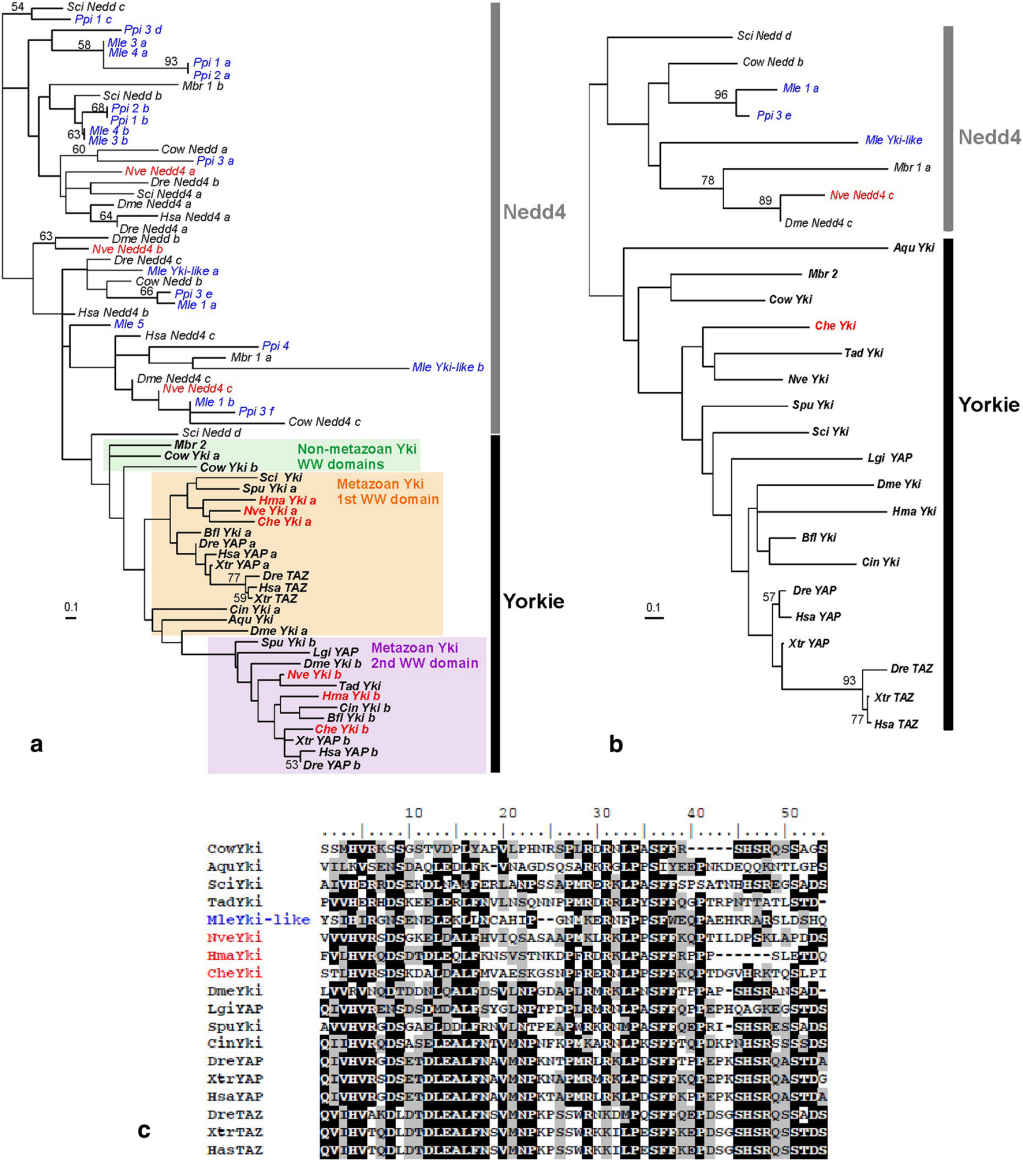
Rooted phylogenetic analyses of Yorkie sequences. **a** Analysis of aligned WW domains (multiple WW domains of the same protein numbered sequentially with letters: *a*, *b*, *c*, etc.). WW domains of Nedd4 were used to root the tree. **b** Combined analysis of the TBD domain and the two WW domains. In **a**, **b** sequences from ctenophores are in *blue* and sequences from cnidarians in *red*; support values are indicated next to the branches when higher than 50 %. **c** Alignment of the TBD domains of MleYki-like (in *blue*) with Yki TBD domains from various organisms (cnidarians in *red*). Species names are indicated by a *three*-*letter code* (see taxonomy in “Methods”): *Aqu: Amphimedon queenslandica, Bfl: Branchiostoma floridae, Che: Clytia hemisphaerica, Cin: Ciona intestinalis, Cow: Capsaspora owczarzaki, Dme: Drosophila melanogaster, Dre: Danio rerio, Hma: Hydra magnipapillata, Hsa: Homo sapiens, Lgi: Lottia gigantea, Mle: Mnemiopsis leidyi, Mbr: Monosiga brevicollis, Nve: Nematostella vectensis, Ppi: Pleurobrachia pileus, Sci: Sycon ciliatum, Spu: Strongylocentrotus purpuratus, Tad: Trichoplax adhaerens, Xtr: Xenopus tropicalis. Scale bar* inferred number of substitutions per site

Several previous studies have addressed the origin and evolution of Hippo pathway genes [19, 32–34], but this one is the first to encompass ctenophores. In addition, published trees of Hpo and Yki [32, 33] did not include closest paralogs (i.e. were unrooted), which is problematic for orthology assessment, whereas in the present study all phylogenetic trees are rooted. Presence/absence scorings deduced from these trees for core Hippo signalling pathway genes (Hpo, Sav, Mats and Warts) as well as the transcriptional co-activator Yki and the transcription factor Sd are summarised in Table 1. Trees for Yki are shown in Fig. 2. Phylogenetic analyses of Cyclins and core Hippo pathway genes other than Yki are shown in Additional file 4.

**Table 1:** Summary of the results of phylogenetic analyses

	Hippo	Salvador	Warts	Mats	Yorkie	Scalloped
*Capsaspora owczarzaki*	+	–	–	+	+	+
*Monosiga brevicollis*	+ (*)	+	–	+	+	+
*Amphimedon queenslandica*	+	– (*)	+	+	+	+
*Sycon ciliatum*	+	+	+	+	+	+
*Trichoplax adhaerens*	+	– (*)	+	+	+	+
*Pleurobrachia pileus*	+	+	+	+	–	+
*Mnemiopsis leidyi*	+	+	+	+	– (●)	+
*Hydra magnipapillata*	+	+	+	+	+ (*)	+
*Clytia hemisphaerica*	+	+	+	+	+	+
*Nematostella vectensis*	+	+	+	+	+	+
*Lottia gigantea*	+	+	+	+	+	+
*Drosophila melanogaster*	+	+	+	+	+	+
*Aedes aegypti*	+	+	+	+	+	+
*Strongylocentrotus purpuratus*	+	+	+	+	+	+
*Ciona intestinalis*	+	+	+	+	+	+
*Branchiostoma floridae*	+	+	+	+	+	+
*Xenopus tropicalis*	+	+	+	+	+	+
*Danio rerio*	+	+	+	+	+	+
*Homo sapiens*	+	+	+	+	+	+

For each species, the table indicates presence (+) or absence (–) of at least one orthologue for each of six core actors of the Hippo pathway. The symbol (*) indicates discrepancies with previous studies (Yorkie absent in *H. magnipapillata* and Salvador present in *T. adhaerens* according to [32]; Hippo absent in *M. brevicollis* and Salvador present in *A. queenslandica* according to [34]). (●) Presence of an atypical Yk-like protein in *M. leidyi*: see text

The most remarkable finding from these analyses is the absence in ctenophores of a true orthologue of Yorkie (Fig. 2; Table 1), whereas clear single orthologues of the other core Hippo signalling pathway genes Hpo, Mats, Warts, Sav and Sd are present (Additional file 4 and Table 1). Ctenophore WW domains with the closest similarity to Yki WW domains are placed in the tree with bilaterian Nedd4 WW domains, rather than with Yorkie WW domains (Fig. 2a). Combined analysis of the conserved Yorkie domains (the TBD domain and the two WW domains; see “Methods”) likewise fails to identify any Yki orthologue in ctenophores (Fig. 2b). Since the non-metazoan holozoans *C. owczarzaki* and *M. brevicollis* both possess a clear Yki orthologue (Fig. 2a, b) as previously shown [34], the absence in ctenophores is clearly derived.

However, the situation is in fact more complex, given that in the released version of genome-predicted *M. leidyi* proteins, there is a sequence annotated as Yki (here referred to as MleYki-like), which contains a TEAD-binding (TBD) domain and two WW domains (respectively, *Mle Yki*-*like a* and *Mle Yki*-*like b* in Fig. 2a). In bilaterians, this secondary structure and the TBD domain itself are unique features of Yorkie proteins. The TBD domain predicted from MleYki-like aligns reasonably well with Yorkie TBD domains from various organisms (Fig. 2c). However, as mentioned above, both WW domains of MleYki-like are more related to Nedd4 than to Yki WW domains (Fig. 2a), even if the second one (*MleYki*-*like b* in Fig. 2a) has a very long-branch casting doubt about its true phylogenetic affinity. The chimerical domain composition of MleYki-like is not due to a bioinformatic assembly error as we could retrieve the gene locus and confirm the presence and sequences of the TBD and WW domains (as they appear in the published genome sequence) by analysis of PCR products from *M. leidyi* genomic DNA (Additional file 5). Thus, the domain combination of MleYki-like probably reflects domain shuffling between ancestral Yki and Nedd4 proteins. An additional surprise was the absence of this protein from our assembly of the *P. pileus* transcriptome, as well as from the published genome of *Pleurobrachia bachei*, and from the extensive available *P. bachei* mRNA data (from all developmental stages including adult; http://neurobase.rc.ufl.edu/pleurobrachia/blast#). Whether the atypical Yki-like gene of *M. leidyi* is functional in this organism remains to be seen, given that the sequence could not be retrieved among *M. leidyi* ESTs available in NCBI. In the genus *Pleurobrachia*, this gene was apparently lost.

Outside ctenophores, our analyses identified a Yorkie orthologue in *Hydra magnipapillata* (as in other cnidarians) contrary to Zhu et al. [32]. Other instances of Yki absences in metazoans according to the same study were in fact due to insufficient genome coverage since we could recover Yki orthologues in the tunicate *Oikopleura* and the acarian *Ixodes* (analyses not shown). In addition, there are noteworthy observations emerging from the analysis of the WW domains present in Yki proteins (Fig. 2a). It has been previously stressed that the secondary structure of Yki in the holozoan *Capsaspora* is identical to that of most metazoan Yki, i.e. with the TBD domain and two WW domains [19]. However, our domain analysis shows that the two WW domains of *Capsaspora* Yki are not respectively orthologous to the first and second WW domains of metazoan Yki proteins (Fig. 2a). Therefore, these two WW domains of *Capsaspora* Yki originated from a domain doubling independent from a similar event that gave the secondary structure typical of metazoan Yorkie. In our sampling, three metazoan taxa have Yki proteins with one WW domain instead of two, but the tree topology clearly indicates that this corresponds to three convergent events whereby one of the WW domains was lost: in sponges (*A. queenslandica* and *S. ciliatum*) (loss of the second WW domain), in the placozoan *T. adhaerens* (loss of the first WW domain) and in the vertebrate Yki paralogue TAZ (loss of the second WW domain, both domains being present in the other vertebrate paralogue YAP).

Concerning the other Hippo pathway genes, we could identify a clear Hpo orthologue in the choanoflagellate *M. brevicollis* (see Table 1 and Additional file 4), which was apparently overlooked in a previous study [34]. Reciprocally, our analyses of Sav sequences, which unlike previous studies included outgroups sequences to root the WW domain and combined trees (see Additional file 4 for details), revealed that previous identification of Sav orthologues in the demosponge *Amphimedon queenslandica* and the placozoan *T. adhaerens* [32, 34] was erroneous, the gene being secondarily lost in both lineages independently (as deduced from the presence of Sav in *M. brevicollis* and in the calcisponge *S. ciliatum*) (Table 1).

### Expression of three cyclins and three Hpo pathway genes in a ctenophore cellular conveyor belt

**Fig. 3:**
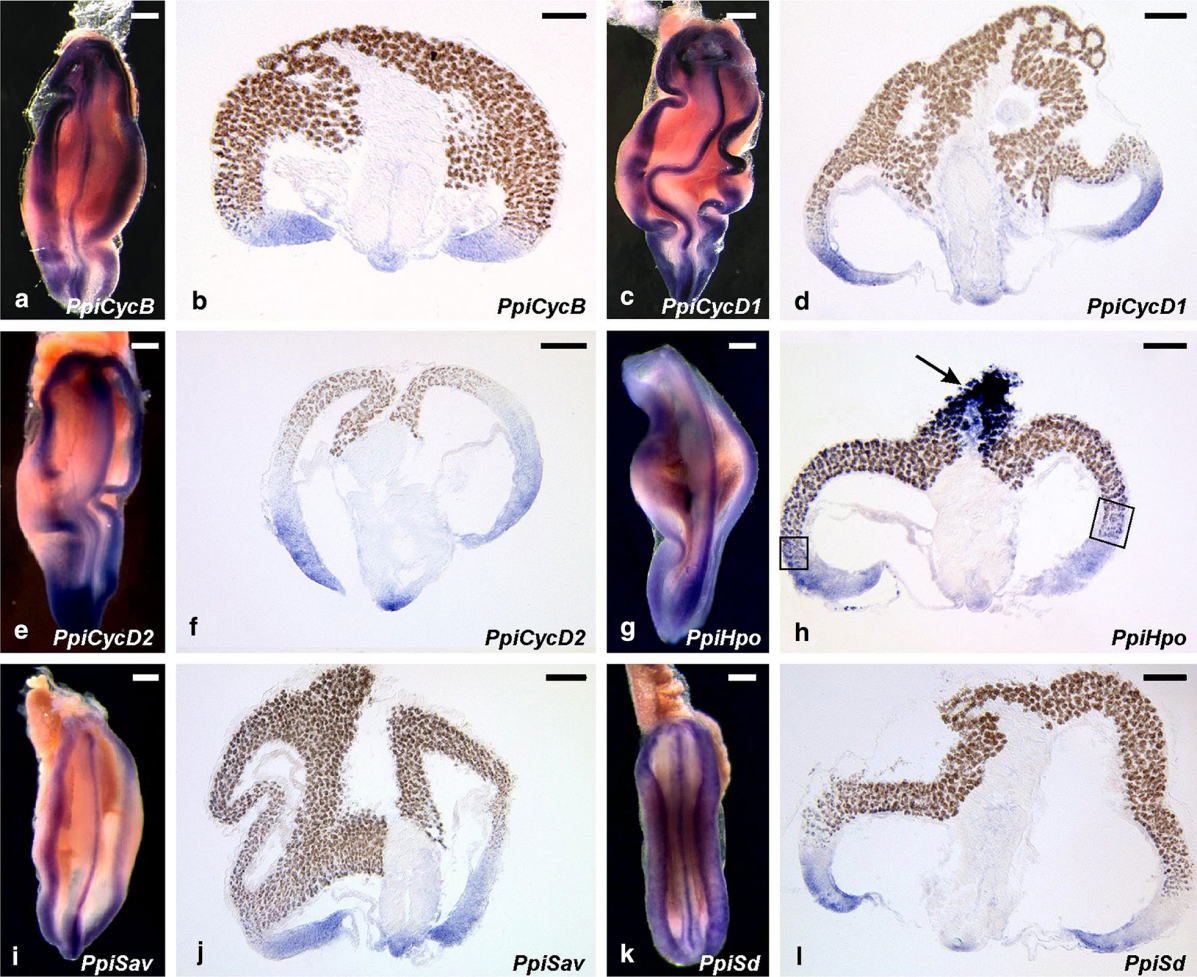
Expression of **a**, **b** *PpiCycB*, **c**, **d** *PpiCycD1*, **e**, **f** *PpiCycD2*, **g**, **h** *PpiHpo*, **i**, **j** *PpiSav*, **k**, **l** *PpiSd* in the tentacle root of *P. pileus*. **a**, **c**, **e**, **g**, **i**, **k** Whole-mount views of tentacle roots (internal side) and **b**, **d**, **f**, **h**, **j**, **l** transverse cryosections towards the middle of the tentacle root (internal side on the bottom). Refer to Fig. 1a–b for interpretations of these pictures. The *brown colour* in **b**, **d**, **f**, **h**, **j**, **l** corresponds to a pigment that appears and accumulates in the cytoplasm of colloblasts as they differentiate. In **h**, *boxes highlight* the colloblast differentiation area where *PpiHpo* is expressed contrary to the other genes, and the *arrow* shows area of strong *PpiHpo* expression at the tentacle/tentillae surface (whereas differentiated colloblasts that are still at the surface of the tentacle root do not express the gene—see more details in Additional file 7). *Scale bars* 100 µm

In the tentacle root of the ctenophore *P. pileus*, cyclin B (*PpiCycB*) and the cyclin D paralogues *PpiCycD1* and *PpiCycD2* have similar expression patterns. In whole-mount views, staining is seen along the three longitudinal ridges housing the stem cells that continuously provide progenitors of colloblasts (lateral ridges) and tentacle core cells (median ridge) (Fig. 3a, c, e). Examination of transverse cryosections indicates that transcripts of these genes are restricted to the distal part of the three ridges (Fig. 3b, d, f) and are turned off along the corresponding cellular conveyor belts before entry into the differentiation zone (materialised in the lateral ridges by appearance of a brown pigment in the differentiating colloblasts; see Fig. 1a, b for interpretation of the ISH pictures of Fig. 3). These expression patterns are closely similar to those previously published for markers of stem cells and undifferentiated progenitors, namely Piwi, Vasa, Bruno and PL10 [49]. Negative controls (with sense probe and with no probe) are provided in Additional file 6.

We examined the expression in the *P. pileus* tentacle root of three orthologues of vertebrates and fly core Hippo pathway components. *PpiSav* and *PpiSd* are expressed similarly as *PpiCycB*, *PpiCycD1* and *PpiCycD2* (and stem cell markers) (Fig. 3i–l). This is also true of *PpiHpo* in the muscular cellular conveyor belt (central part of the tentacle root, Fig. 3h). However, in the lateral (colloblastic) cellular conveyor belts, *PpiHpo* expression is turned off later than the other genes as indicated by faint signal extending to the colloblast differentiation zone (boxes in Fig. 3h). Furthermore, whereas no *PpiHpo* expression is detected in mature colloblasts on the tentacle root surface (Fig. 3h; Additional file 7), this gene appears to be re-activated at a high level in colloblasts and other epidermal cells at some distance of the tentacle insertion point (no signal in tentacle base; strong signal in the rest of tentacle and in tentillae: see arrow in Fig. 3h and details in Additional file 7).

### Expression of two cyclins and four Hpo pathway genes in a cnidarian cellular conveyor belt

**Fig. 4:**
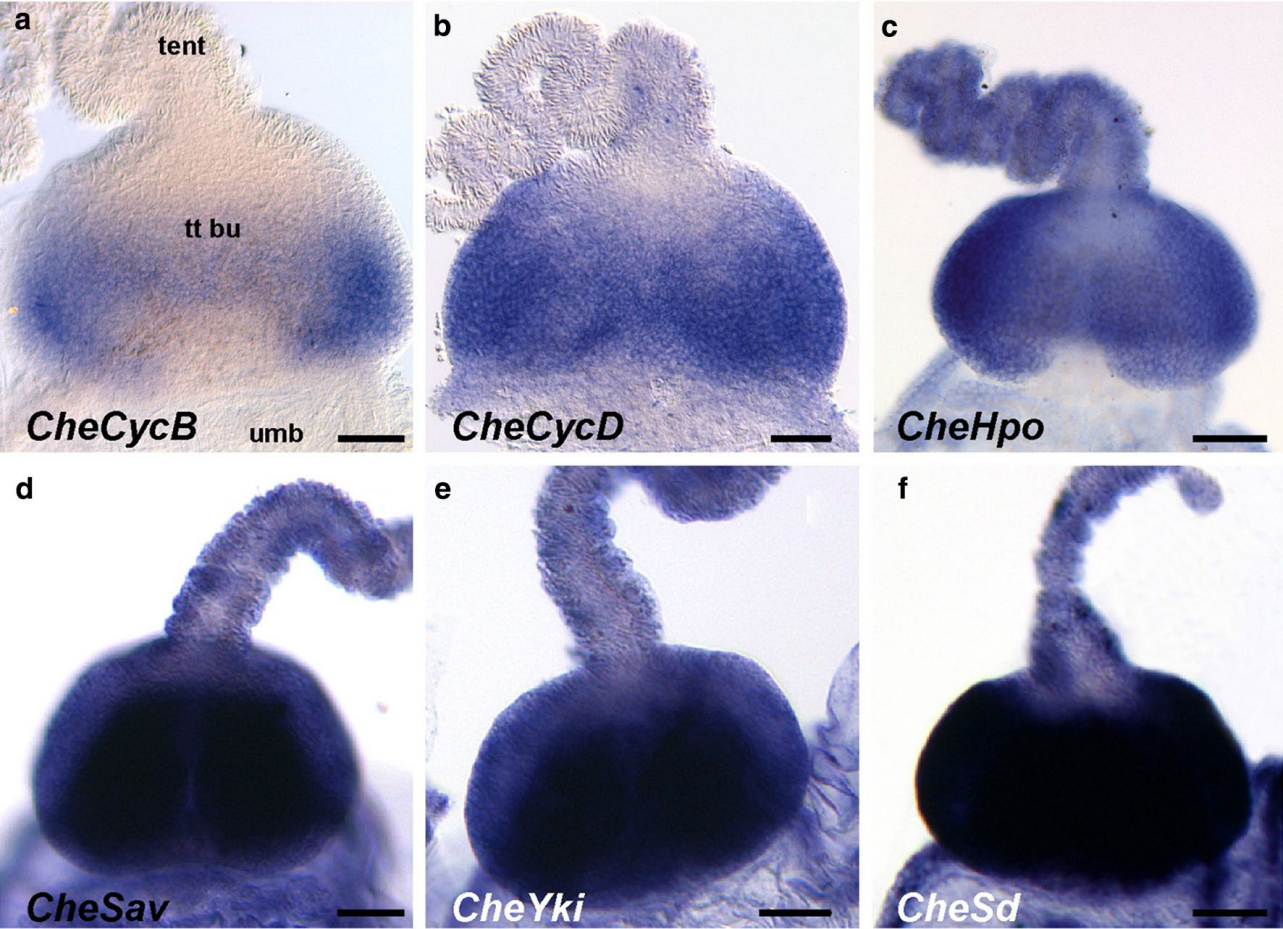
Expression of **a** *CheCycB*, **b** *CheCycD*, **c** *CheHpo*, **d** *CheSav*, **e** *CheYki* and **f** *CheSd* in the tentacle bulb of the *C. hemisphaerica* medusa. Refer to Fig. 1c for interpretation of these pictures. *tent* tentacle, *tt* *bu* tentacle bulb, *umb* periphery of the umbrella. *Scale bars* 50 µm

In the *C. hemisphaerica* tentacle bulb (the cellular conveyor belt responsible for cell renewal at the base of tentacle), *CheCycB* is expressed in two symmetrical areas located in the proximal half of the tentacle bulb (Fig. 4a). This is where populations of interstitial stem cells have been previously identified, notably based on expression of stem cell marker genes [46, 47, 51]. Transcripts of *CheCycD* show a more extended distribution along the bulb axis, being excluded only from the most distal area of nematogenic ectoderm (Fig. 4b). This might reflect maintenance of cyclin D expression after cell cycle exit and during differentiation in *C. hemisphaerica*, which would be reminiscent of the maintained expression of *Cyclin D3* (unlike the other cyclins) during differentiation in the context of spermatogenesis in mammals [57].

**Fig. 5:**
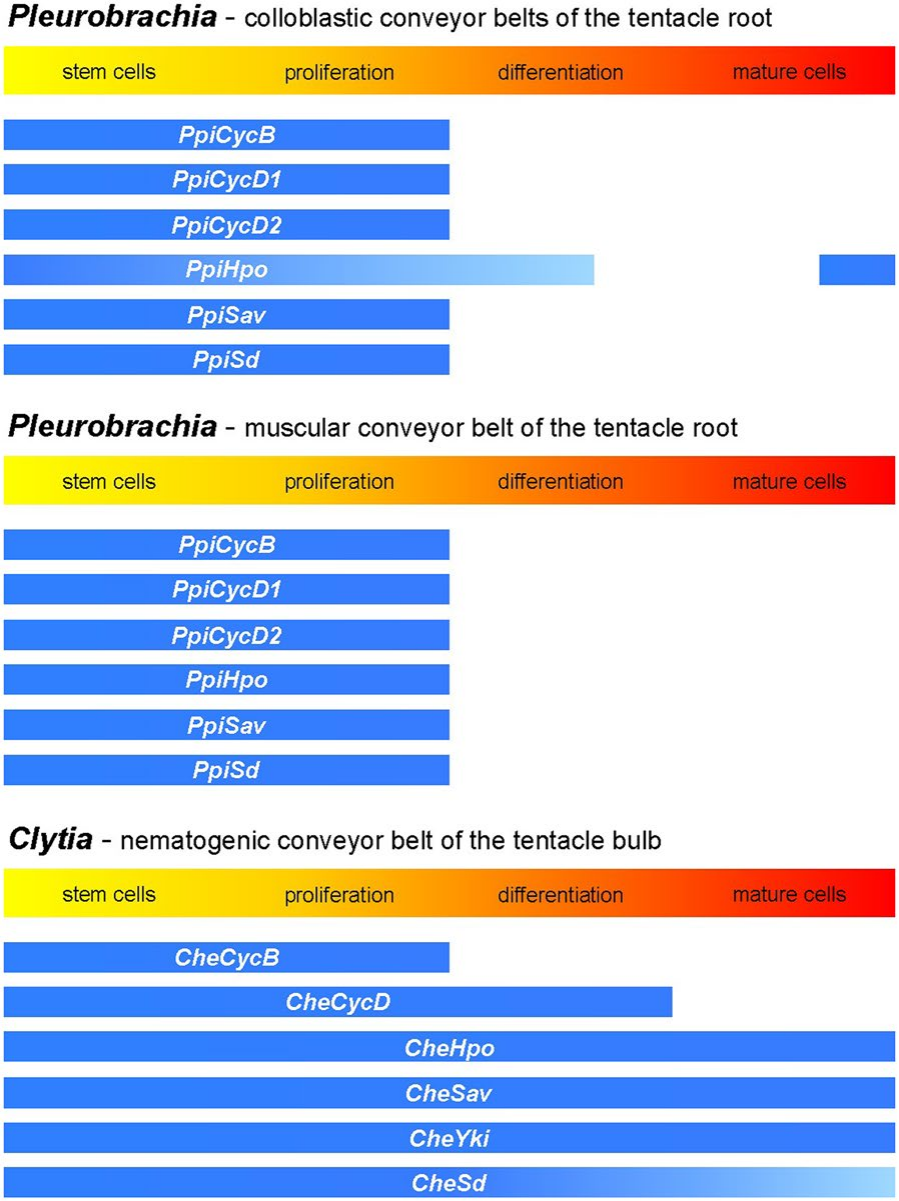
Interpretation of the in situ hybridisation data presented in Figs. 3, 4 in terms of gene expression domains along cellular conveyor belts. A *lighter blue tone* indicates lower transcript level

The *C. hemisphaerica* homologues of Hippo pathway genes investigated here show expression features very different from those of cyclins in the tentacle bulb. Indeed, *CheHpo* (Fig. 4c) and *CheSav* (Fig. 4d) transcripts were detected not only throughout the tentacle bulb nematogenic ectoderm, but also at strong level in the mature tentacle. This is also true for *CheYki* (Fig. 4e). *CheSd* displays strongest expression in the nematogenic ectoderm of the bulb, with lower expression in the bulb tip and the tentacle (Fig. 4f). Outside from the tentacular system, these four genes appear to be ubiquitously expressed throughout the medusa (see in situ hybridisation for *CheHpo, CheYki, CheSav* and *CheSd* in whole medusae in Additional file 8; negative controls in Additional file 6). A graphical summary of expression data in *P. pileus* and *C. hemisphaerica* cellular conveyor belts is presented in Fig. 5.

### Immunolocalisation of Yorkie in the *C. hemisphaerica* medusa

We designed an antibody specifically targeting the *C. hemisphaerica* Yorkie protein (see “Methods”). In Western blot (data presented in Additional file 2), the antibody stains two closely spaced bands around 38 kDa, consistent with the predicted size of the CheYki protein (351 amino-acids × 110 = 38.61 kDa). We interpret these two bands as representing different isoforms or different post-translational modifications of CheYki. The two bands are lost when using antiserum pre-incubated with the immunising peptide and are absent with the pre-immune serum. We also performed whole-mount immunolocalisation controls with the pre-immune serum and with the secondary antibody (Additional file 3), which gave no signal except in the bulb endoderm. All these results strongly support specificity of the anti-CheYki antibody.

In bilaterians, Yorkie is translocated into the nucleus when the Hippo pathway is inactive, whereas this protein is cytoplasmic if the pathway is active (see “Background”). We used the anti-CheYki antibody to look for differences in subcellular localisation of the Yorkie protein within medusa tissues. Since we know that cellular proliferation is restricted to specific areas of the medusa, this offers the opportunity of comparing the localisation of Yorkie in highly proliferative tissues versus non-proliferative ones. Prior to fixation and treatment with the anti-Yorkie antibody, living medusae were incubated during 2 h with the thymidine analogue EdU in order to label replicating nuclei; before observation, all nuclei were in addition stained using Dapi.

**Fig. 6:**
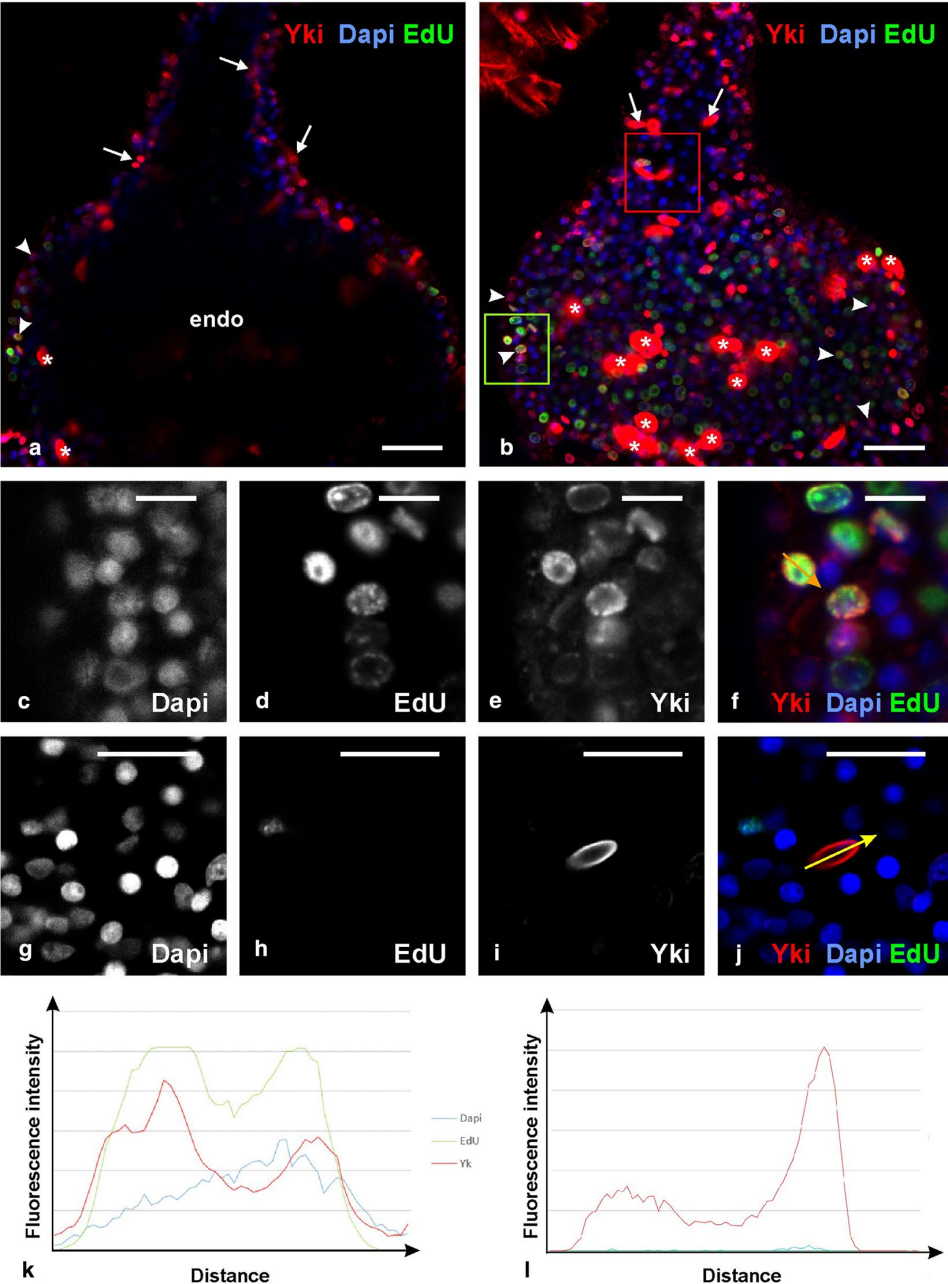
Immunolocalisation of Yorkie and EdU labelling of proliferative nuclei in the *C. hemisphaerica* tentacle bulb. **a**, **b** Confocal longitudinal sections of the whole tentacle bulb and base of tentacle, at two different focal levels (tangential in **b** and deeper in **a**), showing anti-Yki (red), Dapi (*blue*) and EdU (*green*) staining. Refer to Fig. 1c for interpretation of these pictures. *endo* endoderm. *White asterisks* in **a, b** indicate capsules of undifferentiated nematoblasts (see text and Additional file 10); white arrows point to differentiated anti-Yki-stained capsules; selected nuclei positive for Yki are indicated by white arrowheads. **c**–**f** higher magnification view of the area *boxed in green* in (**b**), showing Dapi (**c**), EdU (**d**) and anti-Yki (**e**) staining. **f** Combination of the three signals (anti-Yki in *red*, Dapi in *blue* and EdU in *green*). **g**, **j** higher magnification view of the area *boxed in red* in (**b**), showing Dapi (**g**), EdU (**h**) and anti-Yki (**i**) staining; (**j**) combination of the three signals (anti-Yki in *red*, Dapi in *blue*, EdU in *green*). The structure visible in **i** and coloured in *red* in **j** is a mature nematocyst. **k** Graph of fluorescence intensities for Dapi, EdU and anti-Yki (see *colour legend* on the *right*), showing their quantified variation based on the picture in **f** along the orange arrow (distance in abscisse is from the proximal extremity of the *arrow*). **l** Graph of fluorescence intensities for Dapi, EdU and anti-Yki (see *colour legend* on the *left*), showing their quantified variation based on the picture in **j** along the yellow arrow (distance in abscisse is from the proximal extremity of the *arrow*). *Scale bars* **a**, **b** 20 µm, **c**, **f** 5 µm, **g**, **j** 10 µm

**Fig. 7:**
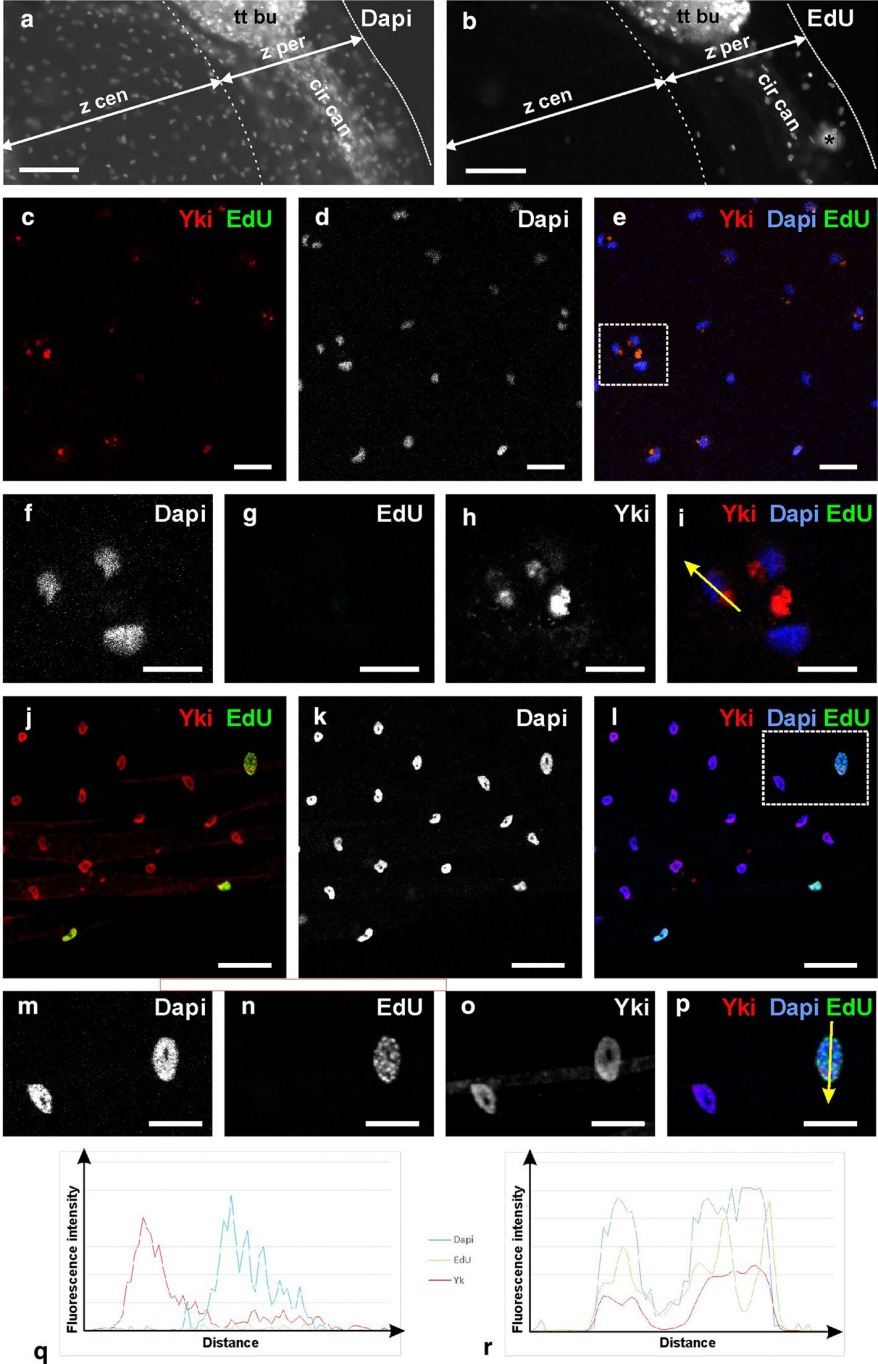
Immunolocalisation of Yorkie and EdU labelling of proliferative nuclei in the *C. hemisphaerica* exumbrellar epidermis. **a** Partial view of Dapi-stained medusa; focus on the exumbrellar epidermis. **b** EdU staining (following 2 h pulse) in the same medusa. *cir can* circular endodermal canal; *tt bu* tentacle bulb; *z cen* central region of the exumbrellar epidermis; *z per* peripheral region of the exumbrellar epidermis. The *asterisk* in **b** corresponds to an artefact. **c**–**e** Detailed view of the central area of the exumbrellar epidermis (*z cen* in **a**, **b**; *blue* in Fig. 8a) with anti-Yki (*red* in **c**, **e**), EdU (*green* in **c**, **e**) and Dapi (*white* in **d**; *blue* in **e**) staining. **f**–**i** Higher magnification view of the *area boxed with dotted line* in (**e**), showing Dapi (**f**), EdU (**g**) and anti-Yki (**h**) staining; **i** combination of the three signals (anti-Yki in *red*, Dapi in *blue* and EdU in *green*). **j**–**l** Detailed view of the peripheral area of the exumbrellar epidermis (*z per* in **a**, **b**; *red* in Fig. 8b) with anti-Yki (*red* in **j**, **l**), EdU (*green* in **j**, **l**) and Dapi (*white* in **k**; *blue* in **l**) staining. **m**–**p** Higher magnification view of the *area boxed with dotted line* in (**l**), showing Dapi (**m**), EdU (**n**) and anti-Yki (**o**) staining; (**p**) combination of the three signals (anti-Yki in *red*, Dapi in *blue* and EdU in *green*). **q** Graph of fluorescence intensities for Dapi, EdU and anti-Yki (see *colour legend* on the *right*), showing their quantified variation based on the picture in **i** along the *yellow arrow* (distance in abscisse is from the proximal extremity of the *arrow*). **r** Graph of fluorescence intensity for Dapi, EdU and anti-Yki (see *colour legend* on the *left*), showing their quantified variation based on the picture in **p** along the *yellow arrow* (distance in abscisse is from the proximal extremity of the *arrow*). *Scale bars* **a**, **b** 50 µm, **c**, **h** 20 µm, **i**, **p** 10 µm

A first important observation is that anti-Yorkie immunofluorescence was detected in all tissues throughout the medusa body (but not in all cells), in consistence with in situ hybridisation showing ubiquitous expression of *CheYki* in the medusa. The characteristics of the anti-Yorkie staining are as expected. When present, it is localised either in the nucleus, or in the cytoplasm, or in both (see examples on Figs. 6f, j, 7i, p and the graphs of Figs. 6k, l, 7q, r—explanations for interpreting the graphs in figure legends). As visible in Fig. 6f and Additional file 9B, in some areas of the medusa, nuclei with and without Yorkie are observed in neighbouring cells.

In the tentacle bulb ectoderm, these different types of subcellular Yki localisation appear to be distributed in space in a way that fits well with the cellular conveyor belt model presented in Fig. 1c and with the known behaviour of Yorkie in bilaterians with respect to cell proliferation and differentiation. As expected [46], EdU was incorporated in many nuclei of the proximal two-thirds of the bulb ectoderm, whereas almost no EdU-positive nuclei were detected in the upper bulb ectoderm and tentacle (Fig. 6a, b). In the proximal two-thirds, many cell nuclei contained anti-Yorkie immunofluorescence (Fig. 6a, b, e, f). In contrast, in the (non-proliferative) distal-most part of the bulb and tentacle ectoderm, we could not detect any positive anti-Yorkie nuclei (Fig. 6j). In addition, strong cytoplasmic anti-Yki signal was associated with differentiation of cells into nematocytes. In the basal half of the bulb, the stained cytoplasmic structures are large ovoid masses of more or less irregular shapes (asterisks in Fig. 6a, b). This is the typical aspect of capsules (nematocysts) in differentiating immature nematoblasts (see [46], and Additional file 10 for details). Closer to and within the tentacle, the capsules keep strong anti-Yki staining but differ by their regular, elongated shape characteristic of mature nematocysts (in the confocal sections of Fig. 6a, b, their aspect is either rounded, oblong or strongly elongated depending on their orientation with respect to the focal plane). A detailed view of a Yki-stained mature nematocyst is shown in Fig. 6i, j. The reader is invited to examine the provided movie of confocal longitudinal Z-sections of the tentacle bulb (Additional file 11) to get a complete picture of the spatial distribution of anti-Yki and EdU signals along the tentacle bulb axis.

**Fig. 8:**
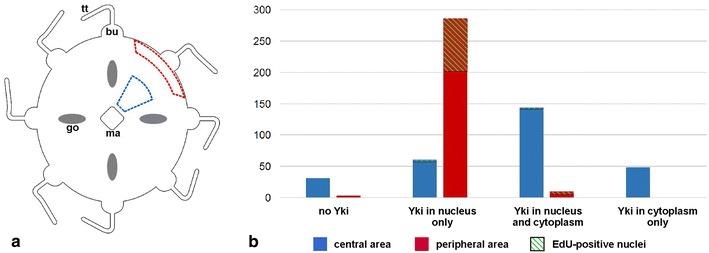
Quantification, in the central versus peripheral area of the *C. hemisphaerica* exumbrellar epidermis, of observed profiles of anti-Yki staining. **a** Diagram of the medusa showing approximate localisation of the two areas (*blue dotted line* central area; *red dotted line* peripheral area). *bu* tentacle bulb, *go* gonad, *ma* manubrium, *tt* tentacle. **b** Graph showing observed numbers of nuclei classified in four categories: no anti-Yki staining; anti-Yki staining in the nucleus but not in associated cytoplasm; anti-Yki staining in nucleus and cytoplasm; anti-Yki staining in cytoplasm but not in nucleus. *Blue* central area; red: peripheral area. Proportions of nuclei in each category that showed EdU staining are indicated with green hatching. A total of 580 nuclei were counted (282 in the *blue zone* and 298 in the *red zone*—raw data given in Additional file 12) using five medusae from the same experiment

Another part of the medusa where we observed interesting spatial differences in Yki subcellular localisation is the epidermis of the exumbrella (the convex surface of the bell), namely between its central and peripheral areas (as outlined in Fig. 8a). Like for the tentacle bulb, the differential behaviour of the Yki protein correlates with different proliferative properties of the tissues. After a 2 h pulse, many EdU-labelled nuclei were observed in the peripheral region of the exumbrellar epidermis (*z per* in Fig. 7b), whereas positive nuclei were almost absent from the rest of the exumbrella (*z cen* in Fig. 7b; see Dapi counter-staining in Fig. 7a). This is not surprising as it certainly reflects medusa bell growth by peripheral extension. We quantified for these two regions anti-Yki-positive and anti-Yki-negative nuclei and presence/absence of signal in the cytoplasm (a relatively easy task thanks to the regular and wide spacing of nuclei in this epithelium as seen in Figs. 7c–e, j–l, unlike in the tentacle bulb). The resulting graph (Fig. 8b) also indicates for each category the number of EdU-positive (green hatches) nuclei (raw numbers given in Additional file 12). The vast majority of cells in the proliferative peripheral area (red zone in Fig. 8a) are Yki-positive with signal restricted to the nucleus (99 % of counted cells; see aspect of the staining in Fig. 7j–l; detailed views Fig. 7m–p; and graph of Fig. 7r). In the non-proliferative central area (blue zone in Fig. 8a), the situation differs sharply, with a large proportion of the cells (68 %) having strong Yki signal in the cytoplasm. The localisation of this signal is remarkable. Whereas exumbrellar epidermal cells are large flat cells, the Yki-stained portion of cytoplasm is small, compact and closely apposed to the nucleus (red signal in Fig. 7e, i), but clearly located outside from the later as shown by absence of Dapi fluorescence superposing with the anti-Yki fluorescence (Fig. 7i and signal quantification in Fig. 7q). There is also detectable anti-Yki signal in most nuclei of the central area (72 %) but at a level which is much lower than in peripheral nuclei (compare Fig. 7e with 7l and 7q with 7r).

To avoid misunderstanding of our interpretation of the data, we wish to emphasise clearly that the described correlation in the tentacle bulb ectoderm and the exumbrellar epidermis between Yki subcellular localisation and proliferative/non-proliferative activity holds true at the tissular scale. We have no idea from the data of whether nuclear/cytoplasmic Yki correlates with mitosis at the level of individual cells. In Fig. 8b, we can see that a quite large proportion of peripheral Yki-positive nuclei are also EdU-positive, and all observed peripheral EdU-positive nuclei are also Yki-positive (but almost all nuclei are Yki-positive in that same area anyway). This nevertheless gives no information about the timing of Yki re-localisation, since medusae were incubated in EdU for 2 h, which may be well longer than the duration of a cell cycle (EdU detected in a nucleus does not mean that the cell was undergoing mitosis at the moment of fixation). However, this is not an important point of concern, as in the literature we found no indication of Yki nuclear-cytoplasmic shift at the cell cycle time scale.

Curiously, the Yorkie protein behaves differently in the subumbrellar epithelium, which forms the concave surface of the bell, with respect to the exumbrellar epithelium (the convex surface). Data for anti-Yorkie immunoreactivity in the *C. hemisphaerica* subumbrellar epidermis are presented in Additional file 9A. Here, all positive cells have Yorkie in the nuclei, and such nuclei are present throughout the epithelium, whereas EdU-labelled nuclei are mostly concentrated close to the radial canals. Again another type of situation was observed in the manubrium, where cell proliferation does not seem to be particularly localised, and cells with Yorkie in the nucleus, in the cytoplasm or both are mixed up throughout the manubrium ectoderm (see data concerning the manubrium in Additional file 9B).

## Discussion

### Loss of Yorkie in ctenophores?

The Yki protein is highly conserved at deep evolutionary level, and the absence of a clear Yki orthologue in ctenophore genomes reported here is unprecedented among metazoans. Indeed, previous reports of Yki losses in several organisms (hydra, the tunicate *Oikopleura*, amphioxus, the acarian *Ixodes scapularis* [32, 33]) were in fact due to insufficient genome coverage, and Yki orthologues were later recovered from these genomes ([33] for amphioxus, this study for hydra, *Ixodes* and *Oikopleura*). For ctenophores, we currently have two genomes and extensive transcriptome data for several other species [38, 39] so that it seems unlikely that a typical Yki orthologue would have been overlooked. The presence of Yki orthologues in *Capsaspora*, choanoflagellates and sponges (Fig. 2) implies that the situation in ctenophores is derived and thus, incidentally, has no bearing on the presently hotly debated phylogenetic position of ctenophores with respect to other early-diverging metazoan lineages.

The enigmatic sequence called MleYki-like in Fig. 2a–c might have originated from a recombination of domains, between the TBD domain of an ancestral Yki protein and WW domains from an ancestral Nedd4 protein. If this scenario is true, then MleYki-like could perhaps be functionally equivalent to Yki even if not being a Yki orthologue in the strict phylogenetic sense. Rather strangely, this gene recovered from the *M. leidyi* genome assembly has apparently no counterpart in *Pleurobrachia* species (see “Results”).

The apparent absence of a true Yki orthologue in ctenophores is particularly puzzling in the light of the presence of well-conserved orthologues of all other core Hippo pathway genes (Hpo, Sav, Mats, Warts and the transcription factor Sd). In addition, Hpo, Sav and Sd are expressed along both cellular conveyor belts of the ctenophore tentacle root in the proliferation zone and turned off in the differentiation zone, consistent with an involvement of activated Hippo pathway in the arrest of cell proliferation and engagement in differentiation like in mammals and fly [2, 4, 5]. In these bilaterian models, the Hippo pathway regulates cell proliferation mainly by modulating the subcellular localisation of Yorkie. Intriguingly, the large majority of residues that are involved in Sd binding with nuclear Yorkie [58] are conserved in ctenophore Sd (data not shown). These molecular interactions were already established in a common ancestor of metazoans and *Capsaspora* [34]. A hypothesis that would be worse testing in future studies could be maintenance of a functional Hippo pathway regulating cell proliferation through Sd in ctenophores, despite replacement of Yki by an alternative co-activator (the Yki-like protein mentioned above, or another one to be uncovered). Ctenophores are highly derived metazoans at the anatomical level [59]. Lack of a typical Yorkie, whereas other Hippo pathway genes are present, adds to several emerging lines of evidence pointing to equally eccentric characteristics for this phylum at the level of molecular biology (e.g. very unusual features of the mitochondrial genome [60, 61]; high amino-acid replacement rate in nuclear proteins [38, 41]; presence of a paralogue of muscular myosin heavy chain II with non-muscular expression [50]).

### Diverging expression characteristics of Hpo, Sav and Sd in ctenophore versus cnidarian cellular conveyor belts

The tentacle root of the ctenophore *P. pileus* and the tentacle bulb of the hydrozoan cnidarian *C. hemisphaerica* are analogous systems both providing differentiating cells at an intense rate for the continuously growing tentacle, and both with localised stem cells furnishing daughter cells that proliferate and differentiate along ordered cellular conveyor belts. However, in ctenophore along conveyor belts of the tentacle root, transcripts of the Hpo, Sav and Sd genes are expressed in the proliferation zone and turned off in the differentiation zone (Figs. 3g–l, 5) (although Hpo is re-expressed in the epidermis of tentacle and tentillae), whereas these genes are expressed all along the nematogenic conveyor belt in the tentacle bulb of *C. hemisphaerica* (Figs. 4c, d, f, 5). They remain expressed in the medusa tentacles, where there is no cell division and only fully differentiated cells. This necessarily implies different regulatory logics for these genes in cellular lineages of ctenophore versus hydrozoan medusa. Nevertheless, ubiquitous expression of these genes along the *C. hemisphaerica* conveyor belt does not preclude a role for the Hippo pathway in this system in regulating the equilibrium between proliferating progenitors and cells exiting the cell cycle to undergo differentiation (and ultimately, size of the tentacle bulb and the rhythm of tentacle growth). Indeed, we know from functional studies in mammals and fly that the molecular functions of Hpo and Sd are principally regulated at the post-transcriptional level (through phosphorylation for the former and through binding to co-activator for the latter), so that these proteins can lead to drastically different functional outputs in different cell populations despite similar levels of transcription at the gene level.

### Nuclear versus cytoplasmic Yorkie in the medusa correlates with the proliferative capacity of tissues

This is furthermore well illustrated by the behaviour of *C. hemisphaerica* Yki at the transcript versus protein levels. In the medusa, in situ hybridisation revealed ubiquitous distribution of Yki transcripts (Additional file 8). In bilaterians, Yki expression is instead restricted to stem cells and proliferating progenitors in some systems (e.g. in the mammalian intestinal crypts [1] and the developing mouse forebrain [62]), but in other systems, Yki expression is maintained in post-mitotic cells, with several reported instances of important functions of cytoplasmic Yki (e.g. regulation of cell fate in fly retinal photoreceptors [63]; see below for additional examples of Yki cytoplasmic functions). Likewise in the planarian *S. mediterranea*, Yki expression is ubiquitous rather than restricted to the totipotent neoblasts [36]. When turning to the subcellular distribution of the Yki protein in the *C. hemiphaerica* medusa, it appears that some cells have Yki in the nucleus, others in the cytoplasm, or in both the nucleus and cytoplasm, and this is reminiscent of the behaviour of Yki in mammals and fly, where post-transcriptional regulation affecting the localisation of Yki is critical to the control of proliferating activity by this protein.

Previous studies using heterologous expression in Drosophila of transgenic Yki and Sd from various organisms (including *Capsaspora* and *Monosiga*) have revealed the ancient origin of a mechanism whereby Yki regulates cell proliferation and organ size through binding with Sd [19, 34]. The present contribution provides the first direct evidence in a non-bilaterian animal of a link between nuclear versus cytoplasmic localisation of Yki, and the propensity of a tissue to undergo cellular proliferation. This represents a powerful additional line of evidence that promotion of cell proliferation by nuclear Yki is a fundamental and ancient mechanism in animal biology, which predated the origin of the Bilateria. Incidentally, a corollary is that negative regulation of proliferation by Yki as reported in the flatworm *S. mediterranea* [36] (unlike the other flatworm *M. lignano* [35]) is certainly a derived situation reflecting changes in the molecular mechanisms of stem cell and proliferation control in this particular lineage of the Platyhelminthes.

The correlation between Yki subcellular localisation and levels of cell proliferation in tissues is clear in the *C. hemisphaerica* tentacle bulb, with Yki-positive nuclei restricted to the proliferation zone in the basal half, whereas in the distal bulb portion and in the tentacle, Yki immunoreactivity is exclusively seen in cell cytoplasms (Fig. 6). A similar situation is observed in the medusa exumbrellar epidermis, with high Yki level in almost all nuclei of the peripheral zone (where EdU was found incorporated in many nuclei following a 2-h pulse) but no cytoplasmic signal, whereas in the rest of the exumbrella (no EdU incorporation), strong cytoplasmic staining was observed in most cells with only very low level in nuclei (Figs. 7, 8). Thus, the behaviour of Yki in the tentacle bulb and exumbrella of *C. hemisphaerica* closely resembles that previously characterised for Yki with respect to EdU staining in a bilaterian cellular conveyor belt (vertebrate embryonic lens [63]). Additional experimental work is required to confirm whether, as suggested by these observations, Yki in cnidarians like its bilaterian counterparts plays a role in promoting cell division, possibly through binding to Sd, and whether transfer of Yki to the cytoplasm is mediated by phosphorylation when the Hippo pathway is activated, thereby promoting cell cycle exit.

Rather intriguingly, unlike the situation in the exumbrella and tentacle bulb, nuclear Yki was observed throughout the subumbrellar epidermis (no Yki immunoreactivity in cytoplasms), whereas cell proliferation (monitored by EdU incorporation) seems restricted to the vicinity of the radial canals (Additional file 9A). Thus, the control of epithelial growth in the two faces (oral and aboral) of the medusa seems to obey different regulatory mechanisms. This is not so surprising since in bilaterians, it is known that the Hippo pathway can have different outputs depending on the cellular context [2]. For example, the mammalian Yki paralogue YAP does not promote proliferation of progenitors in the hematopoietic system and in the heart, unlike in most other embryonic and adult cell renewal systems.

A rather unexpected additional finding was that in nematoblasts and nematocytes, the Yki protein localises to nematocysts (the capsules housing the coiled tubule of nematocytes) (Fig. 6a–b, j). This observation is suggestive of a specific role of cytoplasmic Yki in nematocytes. There are substantial indications in bilaterians that Yki activity is not limited to its transcriptional co-activator role in the nucleus but that there are also cytoplasmic functions of Yki [12]. In developing and adult epithelial airways, a nuclear to cytoplasmic shift in Yap localisation is crucial for Sox2 expression and the specification and differentiation of epithelial progenitors [30]. Moreover, mammalian cytoplasmic YAP can have an important role in cell adhesion and polarity [31]. For example, YAP forms a complex with polarity complex proteins Crb, Pals1 and Patj at the apical side of cells [64]. Accumulation of cytoplasmic YAP was observed at the apical extremity of differentiated lens cells [63], and it was proposed that YAP could structurally stabilise assembled apical polarity complex proteins at epithelial cell apical junctions. In addition, the other mammalian Yki paralogue TAZ is known to interact with cilia-related proteins and double mutants of this gene develop polycystic kidneys [12]. Cytoplasmic YAP and TAZ also play an important role in regulating β-catenin [65]. We therefore hypothesise that cytoplasmic Yki acquired a novel role during cnidarian evolution in the context of the specialised capsule (nematocyst) of nematocytes.

## Conclusions

In this study, we obtained the first indirect evidence for a conserved role of the Hippo pathway in regulation of cell proliferation and growth in a ctenophore and a cnidarian. The co-activator Yki has been secondarily lost or uniquely modified through protein domain recombination in ctenophores, implying substantial changes in the protein interactions that mediate Hippo inputs in this particular non-bilaterian lineage. In the medusa of the cnidarian *C. hemisphaerica*, nuclear localisation of Yki strongly correlates with areas of intense cellular proliferation, at least in the tentacle bulb and in the exumbrellar epidermis. This finding strengthens the hypothesis of an ancient role of Yki switches from the nucleus to the cytoplasm in the arrest of cell proliferation and initiation of differentiation.

The perspectives for further experimental work are manifold. It would be desirable to describe the distribution of Yki in nuclei versus cytoplasm in other life stages of *C. hemisphaerica* (embryonic development, planula larva, polyp colony) using the anti-CheYki antibody designed in this study. In addition to monitoring areas of cell division with EdU as in the present work, it would also be of interest to look for a link between Yki localisation and apoptosis, since in bilaterians nuclear Yki not only promotes cell division but also inhibits apoptosis [66]. Drugs affecting the Hpo pathway would be extremely useful to gain a better understanding of its functions (notably with respect to organ size), especially since tools to inactivate or upregulate specific genes in *P. pileus* or in the *C. hemisphaerica* adults (polyps and medusae) are not yet established. In fly and mammals systems, involvement of the Hippo pathway and Yki in regulation of organ size is under control of the membrane proteins Fat and Ds [2, 4], with polarised gradient of Ds expression along the organ axis being thought to be a particularly important cue [6]. It would therefore be interesting to investigate the expression and function of their homologues in *C. hemisphaerica* (and *P. pileus*) and correlatively the potential links between the Hippo pathway and planar cell polarity. Possible crosstalks between the Hpo pathway and other signalling pathways such as the insulin/IGF, Wnt and notch pathways (abundantly documented in bilaterians [12, 67]) would also be much worse investigating in cnidarians and ctenophores.

## Additional files

10.1186/s13227-016-0041-y **Alignments used to build the trees**. This file contains the alignments used for phylogenetic analyses, given in FASTA format: Yorkie WW domains (page 2), Yorkie combined (page 5), cyclins (page 7), Salvador WW domains (page 11), Salvador combined (page 13), Hippo (page 15), Warts (page 18), Mats (page 23), Scalloped (page 27).
10.1186/s13227-016-0041-y **Validation of the anti-CheYorkie antibody by Western blot**. Total protein extracts from *C. hemisphaerica* medusae were loaded on three lanes of an electrophoresis gel and then transferred onto a nitrocellulose membranes. After Ponceau red staining, the three lanes were separated for incubation with (lane 1) anti-CheYki antiserum; (lane 2) anti-CheYki antiserum that was previously incubated with the immunising peptide; and (lane 3) pre-immune serum. The results of these Western blot experiments suggest that the anti-CheYki antibody detects the CheYki protein with a satisfactory level of specificity.
10.1186/s13227-016-0041-y **Negative controls of whole-mount immunolocalisation experiments.** *C. hemisphaerica* medusae incubated with pre-immune serum and with secondary antibody are compared with medusae incubated with the anti-CheYki antibody (in the same experiment). The negative controls show background signal in the endoderm of the tentacle bulb but no signal in the ectoderm.
10.1186/s13227-016-0041-y **Phylogenetic analyses of Cyclins and core Hippo pathway genes** **(other than Yorkie)**. Maximum likelihood trees are shown for cyclins (page 2; with comment on page 3), Sav WW domains (page 4), Sav combined WW and SARAH domains (page 5), Hippo (page 6), Warts (page 7), Mats (page 8) and Sd (page 9).
10.1186/s13227-016-0041-y **Results of PCR amplification and sequencing of a part of the** ***Mle Yki*** **-** ***like*** **genomic locus.** Graphical representation of our PCR amplification and sequencing of the 5′ part of the *Mle Yki*-*like* genomic locus from *Mnemiopsis leidyi* genomic DNA. The results confirm the domain structure of the gene as predicted from the genome sequence (with one TBD and two WW domains).
10.1186/s13227-016-0041-y **Negative controls of ISH experiments.** The pictures show absence of colorimetric signal in *C. hemisphaerica* and *P. pileus* specimens when the hybridisation step of the ISH protocol was performed with either no probe or with a sense RNA probe.
10.1186/s13227-016-0041-y **Details of** ***PpiHpo*** **expression in the tentacular apparatus.** The pictures show that although *PpiHpo* is turned off in the differentiation zone of the colloblasts in the lateral cellular conveyor belts of the tentacle root (cf. Figure 3h), it is later re-expressed at a strong level in the epithelium of the tentacle axis and tentillae, starting from a point at a short distance from tentacle basis.
10.1186/s13227-016-0041-y **In situ hybridisation for** ***CheHpo, CheYk, CheSav*** **and** ***CheSd*** **in whole medusae.** These pictures show ubiquitous expression of *CheHpo, CheYk, CheSav* and *CheSd* in the *C. hemisphaerica* medusa. Scale bars: 100 µm.
10.1186/s13227-016-0041-y **Anti-Yorkie immunoreactivity in the** ***C. hemisphaerica*** **subumbrellar epidermis (A) and in the manubrium (B)**. In (9A), the three pictures are focused on the subumbrella. *rad can*: radial canal; *tent*: tentacle (superimposed). (1) and (2) are conventional epifluorescence microscopy views; (3) is a confocal section. (9B) shows a confocal section in the manubrium epidermis, with graphs of Dapi and anti-Yorkie immunofluorescence along the four coloured arrows on the picture.
10.1186/s13227-016-0041-y **Additional details about anti-Yki-stained capsules of nematoblasts and nematocysts.** The upper picture is a confocal section of a tentacle bulb similar to that of Fig. 6b, but with red decreased and blue increased in order to de-saturate the Yki signal in nematoblast capsules and to allow visualisation of associated nuclei. The lower panel shows the aspect of capsules stained with an anti-glutamate antibody.
10.1186/s13227-016-0041-y **Movie showing confocal Z-sections of a** ***C. hemisphaerica*** **medusa tentacle bulb**. The preparation was stained with anti-Yorkie antibody (red), EdU (2-hour pulse; green) and Dapi (blue). The movie shows successive longitudinal sections of the same tentacle bulb.
10.1186/s13227-016-0041-y **Counts of anti-Yki cellular profiles in the exumbrellar epidermis.** In this table are given the numbers that were used to build the graph of Fig. 8b.

## Abbreviations

BSA: bovine serum albumin
Dapi: 4′,6-diamidino-2-phenylindole
DIC: differential interference contrast
EDTA: ethylenediaminetetraacetic acid
EdU: 5-ethynyl-2-deoxyuridine
EST: expressed sequence tag
Hpo: Hippo
ISH: in situ hybridisation
ML: maximum likelihood
PBS: phosphate-buffered saline
PBT: phosphate-buffered saline with Tween
PFA: paraformaldehyde
RNAseq: RNA sequencing
Sav: Salvador
Sd: Scalloped
SDS: sodium dodecyl sulphate
TBD: TEAD-binding domain
TBS: tris-buffered saline
Yki: Yorkie
