# Supplementary material for: Comparative study of Hippo pathway genes in cellular conveyor belts of a ctenophore and a cnidarian
Source: EvoDevo. 2016 Feb 19;7:4. doi: 10.1186/s13227-016-0041-y (PMC4761220; doi:10.1186/s13227-016-0041-y)
Supplement: Supplementary file 1 — 10.1186/s13227-016-0041-y Alignments used to build the trees. This file contains the alignments used for phylogenetic analyses, given in FASTA format: Yorkie WW domains (page 2), Yorkie combined (page 5), cyclins (page 7), Salvador WW domains (page 11), Salvador combined (page 13), Hippo (page 15), Warts (page 18), Mats (page 23), Scalloped (page 27). [file 13227_2016_41_MOESM1_ESM.doc]

**Comparative study of Hippo pathway genes in cellular conveyor belts of a ctenophore and a cnidarian**

A. Coste, M. Jager, J.-P. Chambon and M. Manuel

**Additional file 1**

Alignments used to build the trees

All alignments given in Fasta format

page

**Yorkie (WW domain only) 2**

**Yorkie (TBD domain + WW domains) 5**

**Cyclins 7**

**Salvador (WW domain only) 11**

**Salvador (WW domains + SARAH domain) 13**

**Hippo 15**

**Warts 18**

**Mats 23**

**Scalloped 27**

**Yorkie (WW domain only)**

>CowNedda

QPLPEGWEVREDPTGRTYYVDNVSRSVVFEKPTL

>Cin_Ykiia

MPLPHGWSAAKTADGQQYYMNHNDRSTTWEDPRI

>Dme_Ykiia

GALPPGWEQAKTNDGQIYYLNHTTKSTQWEDPRI

>HmaYkiia

NYLPSGCEMRTTASGQKYYINHQNQSTSWQDPRK

>SciYkii

GQLPPGVQAAVTATGQRYFIDHINKTTSWEDPRQ

>CowYkiib

NPLPAGWEMAMHSDGIPYFINHRKRTTTWIDPR-

>Spu_Ykiia

PNLPSGWEMAVTPTGQKYFLDHSNQQTTWEDPRK

>Che_Ykiia

NQLPPGWESRTTPQGQSYFIDHFNRRTTWDDPRK

>Nve_Ykiia

NQLPPGWEMRTSPTGQPYFMNHYEQITTWQDPRK

>Dre_TAZ

LPLPPGWEMAFTPNGQKYFLNHIEKITTWHDPRK

>Xtr_TAZ

LPLPPGWEMAFTHTGQRYFLNHIEKITTWQDPRK

>Hsa_TAZ

LPLPPGWEMTFTATGQRYFLNHIEKITTWQDPRK

>BflYkiia

GPLPPGWEMAHTASGQRYYLNHNNQTTTWEDPRN

>Xtr_YAPa

VPLPPGWEMAKTPSGQRYFLNHMEQTTTWQDPRK

>Dre_YAPa

MPLPPGWEMAKTPSGQRYFLNHNDQTTTWQDPRK

>Hsa_YAPa

VPLPAGWEMAKTSSGQRYFLNHIDQTTTWQDPRK

>Lgi_YAP

VPLPPGWERAYTPEGEVYYIHHVERTTSWFHPSV

>Mbr2

VSLPHGWEMALTPDGLPYFIDHVNKRTSWEDPRS

>HmaYkiib

DDLPEGWERAVTAEGEVYFINHQTKTTSWFDPRL

>Dme_Ykiib

GPLPDGWEQAVTESGDLYFINHIDRTTSWNDPRM

>Cin_Ykiib

IPLPSGWEQAATPQGEIYFINHQTKSTSWVDPRF

>Spu_Ykiib

GPLPINWEQAVTPEGEVYFINHVERTTTWLDPRI

>BflYkiib

IPLPEGWEQATTPEGEIYFINHRTQTTTWLDPRL

>Tad_Ykii

AKLPDGWEKAFTPEGQVYFVNHITRTTSWNDPRR

>Nve_Ykiib

NSLPDGWEQAITPEGEVYFINHITRTTSWIDPRN

>Che_Ykiib

GPLPDGWEQAHTPEGEVYFINHKKQTTSWSDPRL

>Xtr_YAPb

GPLPDGWEQALTPEGETYFINHKNKTTSWLDPRL

>Hsa_YAPb

GPLPDGWEQAMTQDGEIYYINHKNKTTSWLDPRL

>Dre_YAPb

GPLPDGWEQAITSEGEIYYINHKNKTTSWLDPRL

>Ppi3a

EPLPEGWEERVDFSNRVVYVDHVNRVVTLDRPTA

>Mbr1b

EPLPPGWEAAYDPHGHKYYIDHNTKTTHRSLPPA

>AquYkii

IPLPPGWEIGKTPDSHTYFINHNERRTQWEDPRL

>Mbr1a

GSLPPGWSAHRHRDGRVFYANHHTKQTTWADPRL

>Ppi_4

WPLPPNWEARYDGNSRYFYINHVTKQTQWKDPRQ

>SciNeddd

KPLPNGWEERLTAEGLRYFVDHNTKTTTFNDPRL

>Dme_Neddb

EPLPPRWSMQVAPNGRTFFIDHASRRTTWIDPRN

>NveNedd4b

DPLPPGWAVQRAPNGRLFFIDHNSRTTTWHDPR-

>Ppi3e

GPLPDGWEMRY-KNGRPFFIQHRTKQTTWHDPRQ

>Mle1a

-PLPEGIEMRYTRSGRPFFINHRTKETTWHDPRQ

>Mle5

GPLPPGWELRMAPNGRPYFVDHNTKTTQFKDPRV

>CowNeddb

GPLPDGWEMKSTASGRPFFVDHNTQKTTWDDPRL

>CowYkiia

SPLPPGWEKGI-KDGLPFFIDHNNKTTTWVDPRT

>DreNedd4c

GLMPTGWEVRSAPSGRPFFIDHNTKTTTWDDPRL

>HsaNedd4b

SFLPPGWEMRIAPNGRPFFIDHNTKTTTWEDPRL

>CowNedd4c

QPLPPGWEMRRNKEGRAFFVDHASKLTQWTDPRL

>Ppi_3f

NALPDGWEMRIHADGRVFYVDHSTKSNT------

>HsaNedd4c

GPLPPGWEERIHLDGRTFYIDHNSKITQWEDPRL

>Mle1b

APLPDGWEMRVHSDGRVFYVDHATKSTQWEDPR-

>Dme_Nedd4c

GPLPEGWEERVHTDGRVFYIDHNTRTTQWEDPRL

>NveNedd4c

GPLPDGWEERIHSDGRVFYIDHETRSTQWEDPR-

>SciNeddc

EPLPEGWEKR-DVNGRSYFVNHLSRTTQWEDPRL

>Ppi_1c

ESLPPSWEKRVMDNGRTYFVNHESRTTQWEDPRI

>DreNedd4b

AGLPPGWEEKQDSKGRIYFVNHNSRTTTWTRPLI

>NveNedd4a

PPLPEGWEERQDANGRTFYIDHTTRTTTWVRPSR

>Dme_Nedd4a

DALPAGWEERQDANGRTYYVNHTARTTQWDRPTV

>HsaNedd4a

PPLPPGWEEKVDNLGRTYYVNHNNRTTQWHRPSL

>DreNedd4a

PALPPGWEERQDNLGRTYYVNHESRTTQWHRPTI

>Ppi_3d

SPLPAGWAKGKDQNGRTFYIDHNSRTTSWEKPKT

>Mle3a

EPLPQGWELKFDQNGRRYYVDHNSRTTSWSRPKP

>Mle4a

EPLPQGWELKFDQNGRRYYVDHNSRTTSWSRPKP

>Ppi_2a

DSLPTGWEVKFDASGRKYYVDHNTRITTWVRPQP

>Ppi_1a

DSLPTGWEVKFDASGRKYYVDHNTRITTWVRPQP

>SciNedd4a

PPLPGNWEERRDPQGRTYFVDHASRTTTWTRPLP

>SciNeddb

LPLPTGWEQRMDTRGRVYYVDHNTRTTTWQRPTV

>Ppi_2b

QPLPRNWEQRRDPRGRPYYVDHNTRTTTWQRPTT

>Ppi_1b

QPLPRNWEQRRDPRGRPYYVDHNTRTTTWQRPTT

>Mle3b

KPLPAGWEQRLDPRGRPYYVDHNTRTTTWQRP--

>Mle4b

KPLPAGWEQRLDPRGRPYYVDHNTRTTTWQR---

>MleYkii-like a

IPLPSGWEMRHAASGMPYFLDHNTKTTTWDDPRK

>MleYkii-like b

DPLPVGWKQ-YHEHGKTMFHNEQTGQRTFVDPRL

**Yorkie (TBD domain + WW domains)**

>Cin_Yki

QIIHVRQDSASELEALFNTVMNPNFKPMKARNLPKSFFTQPDKPNHSRSSSSDSMPLPHG

WSAAKTADGQQYYMNHNDRSTTWEDPRIIPLPSGWEQAATPQGEIYFINHQTKSTSWVDP

RF

>Dme_Yki

LVVRVNQDTDDNLQALFDSVLNPGDAPLRMRKLPNSFFTPPAP-SHSRANSAD-GALPPG

WEQAKTNDGQIYYLNHTTKSTQWEDPRIGPLPDGWEQAVTESGDLYFINHIDRTTSWNDP

RM

>HmaYki

FVLHVRQDSDTDLEQLFKNSVSTNKDPFRDRKLPASFFRPPP------SLETDQNYLPSG

CEMRTTASGQKYYINHQNQSTSWQDPRKDDLPEGWERAVTAEGEVYFINHQTKTTSWFDP

RL

>SciYki

AIVHERRDSEKDLNAMFERLANPSSAPMRERKLPASFFSPSATNHHSREGSADSGQLPPG

VQAAVTATGQRYFIDHINKTTSWEDPRQ--------------------------------

--

>Spu_Yki

AVVHVRGDSGAELDDLFRNVLNTPEAPWRKRNMPASFFQEPRI-SHSRESSADSPNLPSG

WEMAVTPTGQKYFLDHSNQQTTWEDPRKGPLPINWEQAVTPEGEVYFINHVERTTTWLDP

RI

>Che_Yki

STLHVRSDSKDALDALFMVAESKGSNPFRERNLPPSFFKQPTDGVHRKTQSLPINQLPPG

WESRTTPQGQSYFIDHFNRRTTWDDPRKGPLPDGWEQAHTPEGEVYFINHKKQTTSWSDP

RL

>Nve_Yki

VVVHVRSDSGKELDALFHVIQSASAAPMKLRKLPPSFFKQPTILDPSKLAPDDSNQLPPG

WEMRTSPTGQPYFMNHYEQITTWQDPRKNSLPDGWEQAITPEGEVYFINHITRTTSWIDP

RN

>Dre_TAZ

QVIHVAKDLDTDLEALFNSVMNPKPSSWRNKDMPQSFFQEPDSGSHSRQSSADSLPLPPG

WEMAFTPNGQKYFLNHIEKITTWHDPRK--------------------------------

--

>Xtr_TAZ

QVIHVTQDLDTDLEALFNSVMNPKPSSWRKKILPESFFKEPDSGSHSRQSSTDSLPLPPG

WEMAFTHTGQRYFLNHIEKITTWQDPRK--------------------------------

--

>Hsa_TAZ

QVIHVTQDLDTDLEALFNSVMNPKPSSWRKKILPESFFKEPDSGSHSRQSSTDSLPLPPG

WEMTFTATGQRYFLNHIEKITTWQDPRK--------------------------------

--

>BflYki

------------------------------------------------------GPLPPG

WEMAHTASGQRYYLNHNNQTTTWEDPRNIPLPEGWEQATTPEGEIYFINHRTQTTTWLDP

RL

>Xtr_YAP

QIVHVRSDSETDLEALFNAVMNPKNAPMRMRKLPDSFFKQPEPKSHSRQASTDGVPLPPG

WEMAKTPSGQRYFLNHMEQTTTWQDPRKGPLPDGWEQALTPEGETYFINHKNKTTSWLDP

R-

>Dre_YAP

QIVHVRGDSETDLEALFNAVMNPKNTPMRLRKLPDSFFTPPEPKSHSRQASTDAMPLPPG

WEMAKTPSGQRYFLNHNDQTTTWQDPRKGPLPDGWEQAITSEGEIYYINHKNKTTSWLDP

RL

>Hsa_YAP

QIVHVRGDSETDLEALFNAVMNPKTAPMRLRKLPDSFFKPPEPKSHSRQASTDAVPLPAG

WEMAKTSSGQRYFLNHIDQTTTWQDPRKGPLPDGWEQAMTQDGEIYYINHKNKTTSWLDP

RL

>Lgi_YAP

QIVHVRENSDSDMDALFSYGLNPTPDPLRMRNLPASFFQPPEPHQAGKEGSTDS------

----------------------------VPLPPGWERAYTPEGEVYYIHHVERTTSWFHP

SV

>Tad_Yki

PVVHERHDSKEELERLFNVLNSQNNPPMRDRRLPYSFFQGPTRPNTTATLSTD-------

----------------------------AKLPDGWEKAFTPEGQVYFVNHITRTTSWNDP

RR

>AquYki

VILKVSENSDAQLEDLFK-VNAGDSQSARKRGLPPSIYEEPNKDEQQKNTLGPSIPLPPG

WEIGKTPDSHTYFINHNERRTQWEDPRL--------------------------------

--

>CowYki

SSMHVRKSSGSTVDPLYAPVLPHNRSPLRDRNLPASFFR-----SHSRQSSAGSSPLPPG

WEKGI-KDGLPFFIDHNNKTTTWVDPRTNPLPAGWEMAMHSDGIPYFINHRKRTTTWIDP

R-

>Mbr2

------------------------------------------------------VSLPHG

WEMALTPDGLPYFIDHVNKRTSWEDPRSVSLPHGWEMALTPDGLPYFIDHVNKRTSWEDP

RS

>Mle1a

-------------------------------------------------------PLPEG

IEMRYTRSGRPFFINHRTKETTWHDPRQ-PLPEGIEMRYTRSGRPFFINHRTKETTWHDP

RQ

>Ppi3e

------------------------------------------------------GPLPDG

WEMRY-KNGRPFFIQHRTKQTTWHDPRQGPLPDGWEMRY-KNGRPFFIQHRTKQTTWHDP

RQ

>CowNeddb

------------------------------------------------------GPLPDG

WEMKSTASGRPFFVDHNTQKTTWDDPRLGPLPDGWEMKSTASGRPFFVDHNTQKTTWDDP

RL

>Mbr1a

------------------------------------------------------GSLPPG

WSAHRHRDGRVFYANHHTKQTTWADPRLGSLPPGWSAHRHRDGRVFYANHHTKQTTWADP

RL

>Dme_Nedd4c

------------------------------------------------------GPLPEG

WEERVHTDGRVFYIDHNTRTTQWEDPRLGPLPEGWEERVHTDGRVFYIDHNTRTTQWEDP

RL

>NveNedd4c

------------------------------------------------------GPLPDG

WEERIHSDGRVFYIDHETRSTQWEDPR-GPLPDGWEERIHSDGRVFYIDHETRSTQWEDP

R-

>SciNeddd

------------------------------------------------------KPLPNG

WEERLTAEGLRYFVDHNTKTTTFNDPRLKPLPNGWEERLTAEGLRYFVDHNTKTTTFNDP

RL

>MleYki-like

YSIHIRGNSENELEKLLNCAHIP--GNMKERNFPPSFWEQPAEHKRARSLDSHQIPLPSG

WEMRHAASGMPYFLDHNTKTTTWDDPRKDPLPVGWKQ-YHEHGKTMFHNEQTGQRTFVDP

RL

**Cyclins**

>SciCycF

MRTILVDWVSCEKAYSPLVVHLAMNLVDRWIRTQLQLLGITSLLLASRYFPITDSSYTVH

MLGEM

>CheCycF

MRCILVDWVAYMKDMSHEVLHAAVSYVDRYLRTKLQLLGISCLLLAAQVHILTDQTYQVK

MIGAF

>NveF

MRYILVDWVALMKDFSSQIVHIAVHCVDQYLRSELQLLGITCILIAAGKDIVTDDTYSVR

MMSCL

>TadF

MRYILIDWVAEMKEFSSEMLCNAIDLVDRYLRSNLQLLGISCMVIASCVDIMTDNTYKIIVR

MFAAV

>BflF

MRFILVDWVASMKDFSTQVLHAAVRCVDRYLRSKLQLVGVASMVLVTAKDILTDNTYKIIVR

MTATL

>HsaF

MRYILIDWVATMKDFTSLCLHLTVECVDRYLRYRLQLLGIACMVICTSKEILTDNTYKIIVR

MVSAL

>XlaF

MRYILIDWVATMKDFSSLCLHMTVGLVDRYLRAKLQLVGIACMVICTEIL--TDNTYKIIVR

MISAL

>PpiCycD1_1

WRQKVTHWIFNHCKCEEGVLPHAVNLFDRYCVEELQVLGATCLFIASETASIGSFAYSRK

WLNTL

>PpiCycD1_2

MRKQVTEWISDI--CEVGVLPHAVNLLDRYLGDELQLIGAVCLFVASENEPIENGRFTRL

WLNSL

>MleCycD

MRSQLTEWISDI--CEDGVLPHAVNLLDRYLAEELQVLGATSLFIASEATPLGGYSYSRS

WLNVL

>DmeD

MRKIVAEWVCAEENCQEEVVLLALNYMDRFLKTQLQILAAACLLLASEPSCRTDNSIYIK

WLSRL

>SpuD

MRKLVVDWVCEEQQREEDVFPLSVNYLDRFLRDKFQLLGATCMFLASETIPLTDNSITLK

FLTKL

>HmaD

MRKLVVSWVCEEQRCEDDVFPLSVNLLDRYLKTQLQLLGTTCMFLASETIPLTDNSVTLD

WLNSL

>CheCycD

MRKLVVTWVCEESKCEDDVFPLAVNLLDRFLKTQLQLLGTSCMFIASETIPLTDNSIKLD

WLNKL

>HsaCycD3

MRKMLAYWVCEEQRCEEEVFPLAMNYLDRYLKAQLQLLGAVCMLLASETTPLTDHAVSRD

WLGKL

>NveD

MRKLVATWVCEEERCEEEVFALSMNYLDRILKFQLQLLGAVCMFIASETSPLTDNSITLD

WLGKL

>BflD

MRQMVATWVCEEQRCEDEVFPLAMNYLDRFLKNHLQLLGAVCMFIASETIPLTDNSIRMD

WLMRL

>HsaD

MRKIVATWVCEEQKCEEEVFPLAMNYLDRFLKSRLQLLGATCMFVASETIPLTDNSIRLQ

MVNKL

>HsaD1

MRKIVATWVCEEQKCEEEVFPLAMNYLDRFLKSRLQLLGATCMFVASETIPLTDNSIRLQ

MVNKL

>XlaD2

MRKIVATWVCEEQKCEEEVFPLAMNYLDRFLKSWLQLLGATCMFLASETIPLTDNSIRLI

MLNKL

>HsaD2

MRRMVATWVCEEQKCEEEVFPLAMNYLDRFLKSHLQLLGAVCMFLASETSPLTDNSIKLE

WLGKL

>XlaD1

MRRMVATWVCEEQRCEEEVFPMAMNYLDRFLKCHLQLLGAVCMFLASETIPLTDNSIKLE

WLGKL

>CowCycE

MRAILIDWVCEEYGMHRETFHLAAEFVDRYLKNNLQLIGTTCMLIASEVRPPTDSACTMK

VLMTL

>Sci_CycE

MRRILFDWVCEVMVLRRETFHLAVNLFDRYMKESLQLIGVTCLFVAGEIYPPTDHLHE-D

EIRAM

>AquCycE

MRTILLDWVCEEYRIHRETYYLSLELFDRFMKEQLQLIGVTCLFIASEIYPPTDGACNFE

FIRVM

>MleCycE

ARAILLDWVSQLYCLKRETFYLSMDYIDRFIKEQLQLVGITALHMAAEIYPPTDNSCSLE

MMKAL

>TadE

MRGILLDWVCESFKMQRETFYMAMDYLDRYLKQKLQLIGTTCLFIAAEIQPPTDSACSIK

LLQTL

>HsaE2

MRSILLDWVCEVYTLHRETFYLAQDFFDRFMKNMLQLIGITSLFIASEIYAPTDGACSLR

MLKAL

>XlaE2

MRSILLDWVSEVYTLHRETFYLAQDFFDRFMKSMLQLIGVTALFIASEIYPPTDGACTLQ

MLKAL

>BflE

MRAILLDWVCEVYRLHRETFYLAQDFIDRFLKHRLQHIGITALFIAAEIYPPTDGACTLD

MLKAL

>HsaE1

MRAILLDWVCEVYKIILHRETFYLAQDFFDRYMKTLLQLIGISSLFIAAEIYPPTDGACSLT

MMKAL

>XlaE1

MRAILLDWVCEVYKIILHRETFYLAQDFFDRFMKSRLQLIGITSLFIAAEIYPPTDGACTTR

MMKDL

>NveE

MRAILLDWVCEVYRLHRETYFLAVDFVDRYLKQRLQLVGTTALFIAAEIYPPTDGACKLQ

QLQDL

>HmaE

MRTVLLDWVCEVYRLHRETFYLAVDYVDRFLKTRLQLVGVTAIFVASEIYPPTDGACTLQ

QLSAL

>CheCycA35

MRSVLLDWVCEVYRLHRETFYLAVDYVDRYLKTRLQLVGVTALFIAAEIYPPTDGACTLQ

QLQCL

>DmeE

MRAILLDWVCEVYKIILHRETFYLAVDYLDRYLKTHLQLIGITCLFVAAEIYPPTDGACTLN

HLQAL

>SpuE

MRAILLDWVCEVYRLHRESFYLAADFVDRYLKTKLQLIGITSLFVAAEIYPPTDGACTLD

QLMTL

>Sci_CycB3

MRAILVDWIQENFELVHETLYLTVAYVDRFLREELQLVGATAMFVASEFSPPCDDAYTRE

ELIQM

>HsaB3

MRAILVDWVQVSFEMTHETLYLAVKLVDLYLKDKLQLLGATAFMIAAEHNSPCDDNYQLS

MLNVL

>TadB

MRMILIDWVQQNFELFHETLYLAVKIVDRFLRDALQLIGATAMLMSSERYPPCDDAYSLD

MCYAL

>PpiCycB3.1

MRAILIDWVQESFELFHETLYLAVRMVDRYLKSQLQLLGVCAIFLASERNPPCGEAYTLK

MCAEL

>MleCycB3

MRAILIDWVQETFELFHETLYLAVRMVDRYLKLHLQLLGVTAIFLASERYPPCDRAYTLK

MCAEL

>PpiCycB3.2

MRAILIDWVQETFELFHETLYLAVRMVDRYLKLNLQLLGVTALFLASGRYPPCDAAYT--

-----

>NveB2

MRAILVDWVQESFELYHETLYLGVRVLDNYLRENLQLVGAVSLYIACERHPPCDDAYQVA

MLNSL

>DmeB2

MRTLLVDWVQETFELNHETLYLAVKIVDLYLKEKLQLLGAAAFFIACERQPPCDGAYNVR

MLRVI

>CowX

MRAILVDWVRLELHLSNETFYLAVNILDRFLRDTLQLVGLTAMFVAAETVIPCDGQFQQE

HLLHM

>SpuB2

MRSVLVDWVQENFELNHETLYLAVKLTDMYLKDLLQLLGATSLFIACERIPPCDDAYSTD

MLKMV

>HmaB2

MRAILVDWVQENFELYHETLYLAVKLVDNFLKEQLQLVGATALLIACEHHPPCDDAYTIN

MFKAL

>BflB

MRAILVDWVQENFELNHETLYQAVKIVDHYLKEKLQLVGATALFMSCERCPPCDDAYRIL

MLIKL

>CheCycB3

MRAILVDWVQENFELYHETLYLAVKITDFYLKELLQLVGATALLLSCERSPPCDDAYTVD

MLTAI

>DmeB1

MRAVLIDWVHLQFHLAAETFQLAVAIIDRYLRTYLQLVGVTALFIATELFPPTDDTYTRQ

MFKAI

>SciCycBd

MRSVLVDWVQQRFKLLQETLYVTVSIIDRFLRTKLQLVGVTSMLIASETYAPTDRAYSQV

QIRAM

>PpiCycB

MRSILVDWVHQKFKLLHETLYLSILILDRYLRDKLQLVGVTSILIASEIYAPTNNAYTLA

MLNEL

>MleCycB

MRGILVDWVHLKFKLLQETLYLTVAILDRYLRDKLQLTGVTCMLIASEIYAPTDNAYTLD

MLNTL

>SciCycBb

MRAILVDWVHSRFQLLQETLYVAVAILDRFLRNQLQLVGVTAMLLASEIYAPTDNTYTAS

QIRAM

>HsaB1

MRAILVDWVHSKFRLLQETLYMCVGIMDRFLRKKLQLVGITALLLASEMFSPTDNAYTRE

MLKEL

>XlaB1

MRAILVDWVHSRFQLLQETLYMGVAIMDRFLRSKLQLVGVTSLLIASEMYTPTDNAYTRE

MLRLL

>NveB1

MRAILLDWVHLKFRLLQETLYITMSIIDRFLKRELQLVGVGAMLLASEMFAPTDHAYTRQ

MFRKL

>HsaB2

MRAILIDWVQMKFRLLQETMYMTVSIIDRFMKKMLQLVGVTAMFIASEMYPPTDNTYTRQ

MLRAL

>CowCycB

MRSILVGWVHQTWPFKQETLYLAVHVLDRFLRTRLQLIGLTSFIIAAEIYIPTHNLFSSQ

DVLVA

>XlaB2

MRAILIDWVQMKFRLLQETMFMTVGIIDRFLKNQLQLVGVTAMFLAAEMYPPTDHTYTRD

MLRVL

>SpuB

MRHILVDWVHLRFHLLQETLFLTVQLIDRFLKGKLQLVGVTAMFIASEMYPPTDQAYTRQ

MLKGL

>HmaB1

MRSILVDWVQSRFNLLQETLYLTIYIIDRYLRAELQLVGVTAMLIASEMYAPTDNAYSRQ

MLKTC

>ChecycB

----------------------QIYTIDRFLRSELQLVGVTAMLIASEMYAPTDNAYSRA

MLKAC

>Sci_CycA

MRTILIDWVAEEYKIILHSQTLHLAVGYVDRFLRGKLQLVGATAVLVASEITVPTDNTYSLE

QIRM-

>DmeA

MRSILIDWVSEEYKIILDTETLYLSVFYLDRFLRSKLQLVGTAAMYIAAEIYPPTDDSYTLR

MLKIL

>TadA

MRSILVDWVSEEYKIILRERTLYLAISYIDRFLRSKLQLVGTAALFIAAEIYPPTDDTYNLK

MLKVL

>HmaA

MRAILVDWVSEEYKIILIPQTLYLSVSYIDRFLRGKLQLVGAACMLVAAEIYPPTDDTYTLR

MLKTL

>SpuA

MRCILVDWVSEEYRLHNETLYLAAAFIDRFLRAKLQLVGTASMFVASEIYPPTDDTYSLR

MLKVL

>ChecycA

MRSVLIDWVAEEYKIILNPQTLFLTVNYIDRFLRGKLQLVGTACMLVASEIYPPTDDTYTLR

MLKTL

>MleCycA

MRSILVDWACTKFKLTDETQYYTIQYIDRFLRSDLQLVGIGAIFVACEICVPTEDTYTER

MLKAL

>BflA

MRCILVDWVAEEYKIILHNETLYLAVSYIDRFLRSKLQLVGTAAMFLASEIYPPTDDTYTLR

MLKVL

>AquCycA

MRCILVDWVCDEFHLLPETLFAAVAYVDRYLRSKLQLVGVTCLYLSAEIHPPTDDTYTKK

QIKM-

>NveA

MRAILVDWVAEEYKIILLPQTLYLTVNYIDRFLRGKLQLVGTACMLLASEIYPPTDDTYTLK

MLKVL

>CowCycA

MRAVLVDWVALEYRLKPETLYLAIGYIDRFLRSKLQLLGIACMFVAAEIFPPADRTYEVE

QIRM-

>HsaA1

MRAILVDWVGEEYKIILQNETLHLAVNYIDRFLRGKLQLVGTAAMLLASEIYPPTDDTYTLR

MLKVL

>XlaA2

MRAILVDWVGEEYKIILQNETLYLAVNYIDRFLRGKLQLVGTAAMLLASEIYPPTDDTYTLK

MLKVL

>XlaA1

MRTILVDWVGEEYKIILRTETLYLAVNYLDRFLRGKLQLVGTAAILLASEIYPPTDDTYSLR

MLKVL

>HsaA2

MRTILVDWVGEEYKIILRAETLYLAVNFLDRFLRGKLQLVGTAAMLLASEIYPPTDDTYTLK

MLKVL

**Salvador (WW domain only)**

>Mbr_2b

HDLPPGWENIEDGDGNIVYYNHKDRVTTRFAPDV

>Ppi1a

SDADPDWEVRVQGTGRVYYRHILTDTCHWNHPNE

>Mlea_00851

KELPPGWELRFLSTGRIYYCHLPTDTQHWNHPHE

>Mbr_2a

IPLPTGWQVGKAPNGKPFFLNHNDFSTHWCHPLL

>Sci1

STLPPGWEAAYADNGRIYYIDHNQGDTHWLHPAV

>Nve1a

CPLPPGWEIAYAPDNKIYYIDHNTQTTHWKHPLE

>Dme_Sava

LPLPPGWATQYTLHGRKYYIDHNAHTTHWNHPLE

>Spu_Sava

LPLPPGWTVDRTMRGRKFFIDHNTQTTHWSHPLE

>Lgi_1a

LPLPVGWSVDWTLRGKKYYIDHNTQTTHWSHPLE

>Lgi_2a

LPLPVGWSVDWTLRGKKYYIDHNTQTTHWSHPLE

>lgi_3

LPLPVGWSVDWTLRGKKYYIDHNTQTTHWSHPLE

>Bfl_1a

LPLPTGWTADYTLRGRKYYIDHNTQTTHWSHPLE

>Xtr_Sava

MPLPPGWTVDWTIRGRKYYIDHNTNTTHWSHPLE

>Aae_Sava

LPLPPGWSVDYTLRGRKYYIDHNTKTTHWSHPLE

>Hsa_sava

LPLPPGWSVDWTMRGRKYYIDHNTNTTHWSHPLE

>Tad4a

GPLPEEWIVKYASSNQPYYIDTSTQTVHWLDPRL

>Tad6

LPLPPGWEAKFDNTGKYFFIDHSTKTTSWQDPRI

>Cow1a

KPLPFGWEMRHTDDDRTYFVNHNDMSTTWLDPRL

>Hma1a

DPLPINWEIAYTKDGEMYFVDHNTGRTQWEDPRK

>Che1a

EPLPNNWEVAFTRDGEQYFVDHNSGRTQWDDPRK

>Spu_MAGIa

GPLPTEWEIAFTETGDMYYIDHRDERTQWLDPRL

>Sci2a

GPLPRNWEIAYTEGGDKYYIDHTAGTTQWLDPRV

>Dme_MAGIa

GPLPPKWETAYTERGELYFIDHNTGTSHWLDPRL

>Aae_MAGIa

GPLPPKWEKAYTDSGEVYFIDHNTGTSHWLDPRL

>Aqu_1a

GPLPSNWEIAYTENNEKYFIDHNTGTTHWLDPRL

>Bfl_MAGIa

GPLPDNWEVAYTDNNEMYFIDHNTGTTHWDDPRL

>Nve2a

GPLPDNWEVAYTETNEKYFIDHKTGTTQWVDPRL

>Lgi_MAGIa

GPLPDNWEMAFTDEGQPYFIDHDTETTHWLDPRL

>Hsa_MAGIa

EPLPKNWEMAYTDTGMIYFIDHNTKTTTWLDPRL

>Xtr_MAGIa

EPLPKNWEMAYTEAGMIYFIDHNTKTTTWLDPRL

>Tad5a

RSLPLGWKALTLENGRVYYVNYVTRKSQWYPPPA

>Nve1b

LESPDGWEQVESPQYGIYYVNHATGSSQREHPAK

>Tad5b

ENLPYGWEKAQDQYGSTYYISHLLQITTSEKPLL

>Tad4b

DELPFQWEVINDPQAGPYYIDHVHRRTQFENPIA

>Sci2b

DELPHGWDRADDVKYGVYYIDYINGRTTLENPVA

>Spu_MAGIb

EELPDGWEKIDDPQYGTYYIDHVNRKTQFNSPSK

>Che1b

NDLPPGWEKVTDPKYGTYYIDHINRRTQYEKPEV

>Hma1b

ESLPPGWEKVDDPKYGTYYIDHVNRKTQYEKPDF

>Bfl_MAGIb

NLLPYGWERIDDPHYGTYYVDHVNRRTQYEKPTM

>Hsa_MAGIb

GELPYGWEKIEDPQYGTYYVDHLNQKTQFENPVE

>Xtr_MAGIb

GELPYGWEKIEDPQYGTYYVDHINQKTQFDNPVL

>Aqu_1b

DELPYGWEKVNDPMFGVYYIDHINRTTQYENPVV

>Nve2b

DELPYGWEKVDDPKYGTYYIDHINKKTQFENPVL

>Lgi_MAGIb

DELPYGWEKVEDPHFGTYYIDHVNRRTQYEYPVI

>DmE_MAGIb

DELPYGWEKIEDSMYGMYFIDHVNRRTQYENPVL

>Aae_MAGIb

DELPYGWEKICDPHYGTYYIDHVNRKTQYENPVL

>Cow1b

NELPFGWEAVTSEE-GTYYVDHVRRQTSWSHPRL

>Ppi1b

SDLPPGWERVFSSEYGEYFVNHFSKTAQLHHPGR

>Mleb_00851

ADLPPGWERVYNNEYGEYFVNHITKTAQFKHPGS

>Lgi_1b

ESLPMGWERIESKEHGVFYVNHILKTAQTHHPCS

>lgi_2b

ESLPMGWERIESKEHGVFYVNHILKTAQTHHPCS

>Spu_Savb

EGLPPGWEKVESREHGTYYVNHVSRTAQYRHPNA

>Bfl_1b

EGLPAGWEKVESAEYGVYYVDHNTKRAQYRHPCA

>Hsa_Savb

EGLPPGWERVESSEFGTYYVDHTNKKAQYRHPCA

>Xtr_Savb

EGLPPGWERVESAEFGVYYVDHINKTAQYKHPCA

>Dme_Savb

EGLPVGWRRVVSKMHGTYYENQYTGQSQRQHPCL

>Aae_Savb

EGLPTGWERHESAQHGTFYYNCITGQAQDSHPYL

**Salvador (WW domains + SARAH domain)**

>Dme_Sav

LPLPPGWATQYTLHGRKYYIDHNAHTTHWNHPLEEGLPVGWRRVVSKMHGTYYENQYTGQ

SQRQHPCLLLQFNMFSLPELEGFDSMLVRLFKQELGTIVGFYERYRRALILEKNRR

>Dme_MAGI

GPLPPKWETAYTERGELYFIDHNTGTSHWLDPRLDELPYGWEKIEDSMYGMYFIDHVNRR

TQYENPVL-

>Hsa_MAGI

EPLPKNWEMAYTDTGMIYFIDHNTKTTTWLDPRLGELPYGWEKIEDPQYGTYYVDHLNQK

TQFENPVE-

>Hsa_sav

LPLPPGWSVDWTMRGRKYYIDHNTNTTHWSHPLEEGLPPGWERVESSEFGTYYVDHTNKK

AQYRHPCAILKWELFQLADLDTYQGMLKLLFMKELEQIVKMYEAYRQALLTELENR

>Aqu_1

GPLPSNWEIAYTENNEKYFIDHNTGTTHWLDPRLDELPYGWEKVNDPMFGVYYIDHINRT

TQYENPVV--

>Mbr_2

IPLPTGWQVGKAPNGKPFFLNHNDFSTHWCHPLLHDLPPGWENIEDGDGNIVYYNHKDRV

TTRFAPDV--

>Lgi_1

LPLPVGWSVDWTLRGKKYYIDHNTQTTHWSHPLEESLPMGWERIESKEHGVFYVNHILKT

AQTHHPCSKLKWDLFRLNELEYFDALIRRIYKQELEELVMSYERYRSALNREKDRQ

>Lgi_2

LPLPVGWSVDWTLRGKKYYIDHNTQTTHWSHPLEESLPMGWERIESKEHGVFYVNHILKT

AQTHHPCSKLKWDLFRLNELEYFDALIRRIYKQELEELVMSYERYRSALNREKDRQ

>lgi_3

LPLPVGWSVDWTLRGKKYYIDHNTQTTHWSHPLE--------------------------

--------KLKWDLFRLNELEYFDALIRRIYKQELEELVMSYERYRSALNREKDRQ

>Lgi_MAGI

GPLPDNWEMAFTDEGQPYFIDHDTETTHWLDPRLDELPYGWEKVEDPHFGTYYIDHVNRR

TQYEYPVI--

>Bfl_1

LPLPTGWTADYTLRGRKYYIDHNTQTTHWSHPLEEGLPAGWEKVESAEYGVYYVDHNTKR

AQYRHPCAKLKWDLFKLPELDCYQEMLMRLYRKELEVVVMSYERYRQTLQQEMERR

>Bfl_MAGI

GPLPDNWEVAYTDNNEMYFIDHNTGTTHWDDPRLNLLPYGWERIDDPHYGTYYVDHVNRR

TQYEKPTM--

>Xtr_Sav

MPLPPGWTVDWTIRGRKYYIDHNTNTTHWSHPLEEGLPPGWERVESAEFGVYYVDHINKT

AQYKHPCAILKWELFQLADLDTYQGMLKLLFMKELERIVKLYEAYRQALITEVETR

>Xtr_MAGI

EPLPKNWEMAYTEAGMIYFIDHNTKTTTWLDPRLGELPYGWEKIEDPQYGTYYVDHINQK

TQFDNPVL--

>Nve1

CPLPPGWEIAYAPDNKIYYIDHNTQTTHWKHPLELESPDGWEQVESPQYGIYYVNHATGS

SQREHPAKLLKWELFRYNELDCWQTILKRMYKKEVEQIVMRYEELRQALQRELERR

>Nve2a

GPLPDNWEVAYTETNEKYFIDHKTGTTQWVDPRLDELPYGWEKVDDPKYGTYYIDHINKK

TQFENPVL------------------------------------------------

>HmaMAGI

DPLPINWEIAYTKDGEMYFVDHNTGRTQWEDPRKESLPPGWEKVDDPKYGTYYIDHVNRK

TQYEKPDF--

>Spu_Sav

LPLPPGWTVDRTMRGRKFFIDHNTQTTHWSHPLEEGLPPGWEKVESREHGTYYVNHVSRT

AQYRHPNA--

>Spu_MAGI

GPLPTEWEIAFTETGDMYYIDHRDERTQWLDPRLEELPDGWEKIDDPQYGTYYIDHVNRK

TQFNSPSK--

>Aae_Sav

LPLPPGWSVDYTLRGRKYYIDHNTKTTHWSHPLEEGLPTGWERHESAQHGTFYYNCITGQ

AQDSHPYL--

>Aae_MAGI

GPLPPKWEKAYTDSGEVYFIDHNTGTSHWLDPRLDELPYGWEKICDPHYGTYYIDHVNRK

TQYENPVL--

>CheMAGI

EPLPNNWEVAFTRDGEQYFVDHNSGRTQWDDPRKNDLPPGWEKVTDPKYGTYYIDHINRR

TQYEKPEV--

>PpiSav

SDADPDWEVRVQPGTgRVYYRHILTDTCHWNHPNSDLPPGWERVFSSEYGEYFVNHFSKT

AQLHHPGR-

>Tad4

GPLPEEWIVKYASSSNQPYYIDTSTQTVHWLDPRDELPFQWEVINDPQAGPYYIDHVHRR

TQFENPIA-

>Tad5

RSLPLGWKALTLENGRVYYVNYVTRKSQWYPPPAENLPYGWEKAQDQYGSTYYISHLLQI

TTSEKPLL--

>Tad6

----------------------------------LPLPPGWEAKFDRNTGKYFIDHSTKT

TSWQDPRI

>Mlea_Sav

KELPPGWELRFLSTGRIYYCHLPTDTQHWNHPHEADLPPGWERVYNNEYGEYFVNHITKT

AQFKHPGS--

>Cow

KPLPFGWEMRHTDDDRTYFVNHNDMSTTWLDPRLNELPFGWEAVTSEE-GTYYVDHVRRQ

TSWSHPRL

>SciSav

STLPPGWEAAYADNGRIYYIDHNQGDTHWLHPAV--------------------------

--------QLEWEGRDMPNLEGWLVMVKRLFLEDVKGIVMKYENYRHALRQEMLTR

>SciMAGI

GPLPRNWEIAYTEGGDKYYIDHTAGTTQWLDPRVDELPHGWDRADDVKYGVYYIDYINGR

TTLENPVA

>CheSav

------------------------------------------------------------

--------YLKWTMFRYTQLDCWQTMLKRLYKKELEQVVLWFEEYRSALSDELDRQ

>HmaSav

------------------------------------------------------------

--------YLKWEMFRYPELDCWQTMLKRLYRKEVEQVVLWYEEYRIALQQEVERR

**Hippo**

>Bflg

LGAGNGGVVTKVKHKPSGLIMARKLIHL-RNQIIRELKVLHECNSPHIVGFYGAFYSDGE

ISICMEYMDGGSLDLILK-KAIHEKILGKISIAVLKGLTYLREKHQIHRDVKPSNILVNS

RGEIKLCDFGVSGQLIDSMA--NSFVGTRSYMSPERLQGTHYSVQSDIWSMGLSLVEMAV

GRYPIPPPEPMAAIFDV--PPESLDP------DYAASSSSPSQAGRPAGRPL-NTF

>Bflh

LQQGGGGISVARSNSLRKDSPPPRRVPR-LQDPVREVSIMRDYHHNNIVEMYDSFLVEDE

LWVIMEFLEGGSLTDIVT-HTMNEEQIATVCKAVLEALVFLHSWGIIHRDIKSDSILLAH

DGKVKLSDFGFCAQVTPDLPKRKSLVGTPYWMAPEVISRLPYGPEVDLWSLAIMVMEMVD

GEPPFFNEPPLQAMRRIRDPPKLKNTVSSRLQGFLEKMLMRDPSQRASAIDLLHPF

>Bflf

IGSGATSVVQVASCKPRNEPCAIKRINL-MEELHKEIQAMRQCNHPNVVNFYTSFVVKEE

LWLVLKLLSGGSLLDIIKHRVLDETSIATVLREVLKGLEYLHNNGQIHRDLKAGNILLGN

DGTVQLADFGVSGWLAGDMARRRTFVGTPCWMAPEVMEEGGYDFKADIWSFGIVAIELAT

GTAPYHKYPPMKVLMLTLQPPSLDTGYGKSFRKLVSACLQKDPAQRPNATELLSPF

>Bfli

LGKGAYGKVCCGL-TSRGELIAVKQVEL-YQRLRDEVDLLQTLRHRNIVRFLGTSLEGNV

VNIFMQFIPGGTLASLLA-RVLEEGVVSRYTRQILIGVEYLHNNNIIHRDLKGNNIMLMP

NGVIKLIDFGCARRVCERLSVSNSQVGTPYWMAPEVVSESGYGVKSDVWSVGCTVFEMLT

GKPPWADMAPMAAIFHIGSVPELP--ASPPAHNFVHACLTRNPAQRPSATQLLHSF

>Bflb

IGSGTYGDVYKARNMQTDELAAIKVIKL-FAIIQQEIIMMKECKHINIVAYFGSYLRRDK

LWICMEFCGGGSMQDIYHITPLKETQIAYVCRETLQGLAYLHSRGKMHRDIKGANILLTD

EGNVKLADFGVSAQITATMAKRKSFIGTPYWMAPEVAAKGGYNQQCDVWAVGITAIEFAE

LQPPMFDLHPMRVLFLMSKPPKLKDKWTPNFHNFVKLALTKNPKKRPTAEKMIHPF

>Bfle

IGHGSFGAVYYARNTRTCEVVAIKKMSY-WQDIIKEVKFLRELRHTNTIEYKGCYLREHT

AWLVMEYC-LGSASDIIEVHPLQEIEIAAICHDGMQGLTYLHNNNKIHRDVKAGNILLTE

DGTVKLADFGSASIVSPA----NSFVGTPYWMAPEVILEGQYDGKVDVWSLGITCIELAE

RKPPLFNMNAMSALYHIAQAPSLSSIWSEEFRNFVDACLQKNPCDRPTAQELLKSY

>Bflc

LGDGAFGKVYKVKNKENGNLAAAKIIEI-LEDYTVEIDILSECSHRHVVALDDAYFHDGK

LWMMIEFCAGGALDDIMLDLPLTEPQIRVICRQMLEALDYLHTHHIIHRDLKAGNVLLTP

EGDIKLADFGVSAKNSNTRQKRDTFIGTPYWMAPEVVLDTPYDCKADIWSLGITLIEFAQ

MEPPNHEMHPMRVLIKISKPPQLEDPWSREFADFLRQCLQKIPEGRPSARELLHPF

>Bfld

----------QGRHTKTGQLAAIKVMDK-EEEIKLEINMLKKYHHRNIATYYGAFIKDDQ

LWLVMEYCGAGSVTDLVKATSLKEEWIAYICREILRGLAHLHSCKVIHRDIKGQNVLLTD

NAEVKLVDFGVSAQLDRTIGRRNTFIGTPYWMAPEVIADATYDYRSDLWSLGITALEMAE

SQPPLCDLHPMRALFLIPRPPRLKS-WSKKFVSFVENCLVKNYHQRPSTEQLLHPF

>Bfla

IGKGSFGEVFKGIDNRTKEVVAIKIIDL-IEDIQQEITVLSQCDSPYVTKYFGSFLKGTK

LWIIMEYLGGGSALDLMK--TFEEHFIATILREILKGLDYLHSEGKLHRDIKAANVLLSE

NGDVKLADFGVAGQLTDTQIKRNTFVGTPFWMAPEVIKQSAYDSKADIWSLGITAIELAK

GEPPNSDLHPMRVLFLIPKPPQLE--YSKSFKDFVESCLNKDPKFRPTAKDLLHKF

>Ppi_Hpo

LGEGSYGCVYKAVYKVSNNPVAIKQIPL-LQDILKEINMMRQCESDFVVKYYGSYFKHND

LWIVMEYCAARSVSDIMRLVPFEEDKIQTILFYTLKGLEYLHGEKKIHRDVKAGNILLTA

DGFAKLADFGVAGQLSDTLTKRNTIIGTPFWMAPEVIQEMGYDCMADIWSLGITTIEMAE

GKPPYADIHPMRAIFMIPTPPSFKQPWSDLFKDFLKNCLKKVPEDRRTAEQLLHDF

>MleHpo

LGEGSYGSVYKAVHKASNSLVAIKKVPL-LQDILHEISMMRQCESDFVVKYYGSYFKNSD

LWIVMEYCAARSVSDMMRLKPFDEDKIQTVLYYTLKGLEYLHGKRKIHRDVKAGNILLTA

DGAAKLADFGVAGQLSDTMTKRNTIIGTPFWMAPEVIQEVGYDCMADVWSLGITSLEMAE

GKPPYADIHPMRAIFMIPTPPSFRQPWGELFKDFLRCCLKKLPEDRLTAEQLLHDF

>Mbr_Hpo

------------------------------------------------------------

------------------------------------------------------------

------------------MARRNTVIGTPYWMAPEVIQEIGYDVKADIWSLGIAAIEMAE

GKPPHAHVHPMRAIFMIPTAPRLRRPWSDQFNDFLAHCLQKNPDLRASAQDLLHPF

>Cow Hpo

LGEGSYGSVFKARHKDTQSILAVKQVPLDLQDIIKEISMIKECDSPFIVKYYGSYFKDTD

LWIIMEFCGAGSVADVMRRKVLEEPEIACILQHALKGLSYLHSKLKIHRDIKAGNILLNH

EGVAKLADFGVAGQLSDAMAKRNTVIGTPFWMAPEVIQEIGYDVKADIWSLGITAIEMAE

GRPPYAEIHPMRAIFMIPTPPTLSEKFSESFNDFLAKCLKKNPAERPTAAELLHPF

>Tad_Hpo

LGEGSYGTVYKAVHKETGQLLAVKKVPV-LQDIIREISIMQQCDCSFVVKYFGSYFENSY

LWIVMEFCGGGSVLDIMRLRTLEESKIATIVRCTLKGLEYLHILRKIHRDVKAGNILLDL

EGNAKLADFGVAGQLTDTMAKRNTVIGTPFWMAPEVIQAIGYDCAADIWSLGITAIEMAE

GRPPYADIHPMRAIFMIPTPPTFQEPWSTDFIDFVSKCLVKVPEQRPSASALLHNF

>Aqu_Hpo

IGEGAYGAVYKALHKESGQLLAIKQVPV-LQDIIKEISIMQQCDSQYVVKYYGSYFKNTD

LWIVMEYCGAGSVSDIMRIIPLNEKEISVIVQYALKGLEYLHFKRKIHRDIKAGNILLNL

DGHAKLADFGVAGQLTDTMAKRNTVIGTPFWMAPEVIQEVGYDCLADIWSMGITAIEMAE

GRPPYAEVHPMRAIFMIPTPPTLKQAFSNEFSDFISRCLVKSPEERPSATSLLHRF

>SciHpo

------------------------------------ISIMQQCDSPYVVRYYGSYFKDQD

LWIVMEYCGAGSVSDIMKRKTLTEPETATILQATLKGLEYLHFKRKIHRDIKAGNILLNS

SGHAKLADFGVAGQLSDTMAKRNTVIGTPFWMAPEVIQEVGYDCRADIWSVGITAIEMVE

GKPPYADIHPMRAIFMIPSPPKFKDEWTPGFRDFLEQCVVKNPEERATATSLLHDF

>Bfl_Hpo

LGEGSYGSVFKAMHKESGQVLAIKQVPV-LQEIIKEISIMQQCDRKLLNNGCGTLFYIHV

LVIVMEYCGAGSVSDIMKLRTLTEDEIAAITSMVLKGLEYLHFMRKIHRDIKAGNILLNT

EGDAKLADFGVAGQLTDTMAKRNTVIGTPFWMAPEVIQEIGYDCLADIWSLGITALEMAE

GKPPYADIHPMRAIFMIPTPPTFRNPWSQEFIDFVSQCLVKNPQQRPSATQLLHPF

>Lgi_Hpo

LGEGSYGSVFKAHDVDGDQYLAIKMVPI-LQEIIKEISIMQQCDSPFIVKYYGSYFKNTD

LWIVMEYCGAGSLSDIMRIRTLNETEIATVLSYTLKGLDYLHSRRKIHRDIKAGNILLNS

DGHAKLADFGVAGQLTDTMAKRNTVIGTPYWMAPEVIQEIGYDCVADIWSLGITALEMAE

GKPPYGDIHPMRAIFMIPTPPSFRKPWSPEFIDFVSKCLIKNPEQRATAQELLHEF

>Hma_Hpo

LGEGAYGSVYKAMHKESGEVLAIKQVPV-LQEIIKEISIMQQCDSPYVVKYFGSYFKNTD

LWIVMEYCGAGSVSDCMRLRTLTEDEIACICRDTLKGLEYLHLRRKIHRDIKAGNILLNT

EGHSKLADFGVAGQLTDTMAKRNTVIGTPFWMAPEVIQEIGYDCKADIWSLGITLLEMAE

GKPPHADIHPMRAIFMIPTPPTFKHPWSKDMIDFVSKCLVKNPDDRLSATALLHPF

>Che_Hpo

LGEGAYGSVYKALHKESGEVLAIKQVPV-LQEIIKEISIMQQCDSPYVVKYYGSYFKNTD

LWIVMEYCGAGSVSDCMRLRTLTEDEIACICRDTLNGLEYLHFRRKIHRDVKAGNILLNT

EGHAKLADFGVAGQLTDTMAKRNTVIGTPFWMAPEVIQEIGYDCKADIWSLGITTLEMAE

GKPPHADIHPMRAIFMIPTPPTFKHPWSTDMKDFVSKCLVKNPEERLSATSLLHQF

>Cin_Hpo

LGEGAYGCVFKAIYKEAGQVVAIKQVPV-LQEIIKEIAIMQQCDSPYVVKYYGSYFKNTD

LWIVMEYCGAGSVSDIIRLRTLTEDEVATILNDTLKGLEYLHFMRKIHRDVKAGNILLNT

NGNSKLADFGVAGQLTDTMAKRNTVIGTPFWMAPEVIQEIGYDCVADIWSLGITAIEMAE

GKPPYSDIHPMRAIFMIPQPPTFRDPWSETFINFVSQCLVKSPAQRATATTLLQDF

>Dme_Hpo

LGEGSYGSVYKAVHKESSSIVAIKLVPV-LHEIIKEISIMQQCDSPYVVRYYGSYFKQYD

LWICMEYCGAGSVSDIMRLRTLTEDEIATILSDTLQGLVYLHLRRKIHRDIKAANILLNT

EGYAKLADFGVAGQLTDTMAKRNTVIGTPFWMAPEVIEEIGYDCVADIWSLGITALEMAE

GKPPYGEIHPMRAIFMIPQPPSFREPWSTEFIDFVSKCLVKEPDDRATATELLHEF

>Aae_Hpo

LGEGSYGSVYKALHKESEQVLAIKQVPV-LQEIIKEISIMQQCDSPYVVKYYGSYFKNTD

LWIVMEYCGAGSVSDIMRLRTLSEDEIATILIDTLKGLEYLHLRRKIHRDIKAGNILLNS

EGHAKLADFGVAGQLTDTMAKRNTVIGTPFWMAPEVIEEIGYDCVADIWSLGITALEMAE

GKPPYGDIHPMRAIFMIPTPPSFRDPWSPEFIDFVSLCLVKNPEERATATDLLHEF

>Nve_Hpo

LGEGSYGSVFKAMHKESGQVVAIKQVPV-LQEIIKEISMMQQCDSPYVVKYYGSYFKNTD

LWIVMEYCGAGSVSDLMKIRTLTEEEIKCVLKYTLKGLEYLHLRRKIHRDIKAGNILLNS

EGHAKLADFGVAGQLTDTMAKRNTVIGTPFWMAPEVIQEVGYDCLADIWSLGITAMEMAE

GKPPYADIHPMRAIFMIPTPPTFRDPWSEDFKDFVSKCLVKNPEERATATELLHSF

>Xtr_Mst2

LGEGSYGSVYKASHKETSQIVAIKQIPV-LQEIIKEIAIMQQCDSLHVVKYYGSYFKNTD

LWIVMEFCGGGSISDIIRLRTLKEDETATILQSTLKGLEYLHFMRKIHRDIKAGNILLNS

EGTAKLADFGVAGQLTDTMAKRNTVIGTPFWMAPEVIQEIGYNCVADIWSLGITAIEMAE

GKPPYAEIHPMRAIFMIPSPPTFRKPWSKDFVDFINLCLVKNPELRSSATELLHPF

>Spu_Hpo

LGEGSYGSVFKAIHKESGQVLAIKQVPV-LQEIIKEISIMQQCDSTYVVKYYGSYFKNTD

LWIVMEYCGAGSVSDIMRRRTLNEAEIATILYSTLKGLEYLHFMRKIHRDIKAGNILLNN

EGNAKLADFGVAGQLTDTMAKRNTVIGTPFWMAPEVIQEIGYDCKADIWSLGITALEMAE

GKPPYAEIHPMRAIFMIPTPPTFRDPWSQDFIDFTSKCLIKSPEDRATATDLLHPF

>Hsa_Mst2

LGEGSYGSVYKAIHKETGQIVAIKQVPV-LQEIIKEISIMQQCDSPHVVKYYGSYFKNTD

LWIVMEYCGAGSVSDIIRLRTLTEDEIATILQSTLKGLEYLHFMRKIHRDIKAGNILLNT

EGHAKLADFGVAGQLTDTMAKRNTVIGTPFWMAPEVIQEIGYNCVADIWSLGITAIEMAE

GKPPYADIHPMRAIFMIPTPPTFRKPWSDNFTDFVKQCLVKSPEQRATATQLLHPF

>Dre_Mst

LGEGSYGSVFKAIHKESGQVVAIKQVPV-LQEIIKEISIMQQCDSPYVVKYYGSYFKNTD

LWIVMEYCGAGSVSDIIRLRTLTEDEIATVLKSTLKGLEYLHFMRKIHRDIKAGNILLNT

EGHAKLADFGVAGQLTDTMAKRNTVIGTPFWMAPEVIQEIGYNCVADIWSLGITSIEMAE

GKPPYADIHPMRAIFMIPTPPTFRKPWSDDFTDFVKKCLVKNPEQRATATQLLHPF

>Hsa_Mst1

LGEGSYGSVFKAIHKESGQVVAIKQVPV-LQEIIKEISIMQQCDSPYVVKYYGSYFKNTD

LWIVMEYCGAGSVSDIIRLRTLIEDEIATILKSTLKGLEYLHFMRKIHRDIKAGNILLNT

EGHAKLADFGVAGQLTDTMAKRNTVIGTPFWMAPEVIQEIGYNCVADIWSLGITSIEMAE

GKPPYADIHPMRAIFMIPTPPTFRKPWSDDFTDFVKKCLVKNPEQRATATQLLHPF

>Xtr_Mst1

LGEGSYGSVFKAIHKESGQVVAIKQVPV-LQEIIKEISIMQQCDSHYVVKYYGSYFKNTD

LWIVMEYCGAGSVSDIIRLRTLTEEEIATILRSTLKGLEYLHFMRKIHRDIKAGNILLNT

EGHAKLADFGVAGQLTDTMAKRNTVIGTPFWMAPEVIQEIGYNCVADIWSLGITSIEMAE

GKPPYADIHPMRAIFMIPTPPTFRKPWSDEFTDFVKKCLVKNPEQRATATQLLHSF

**Warts**

>Uma_St38

RAAELEDRLHLSDERKARQLAQLGRRESNFLRLRRTRLGLDDFRTVKVIGKGAFGEVRLV

QKTDTGKIYAMKTLRKSEMFKKDQLAHVRAERDVLAESNSPWVVQ-YSFQDTAYLYLLME

FLPGGDLMTMLIKYDTFSEDVTRFYMAECVLALEGIHKLGFIHRDIKPDNILIDAKGHIK

LSDFGLSTGFHKQHRKLAYSTVGTPDYIAPEIFLQQGYGNECDWWSLGAIMFECLCGYPP

FCSENAHDTYRKILAWRETLQFPDDSPEAEDMIRRLISAPENRLGSASEIKGHAFFVDWI

RDAPFIPQLKSITDTSYFPFLGYTYRRY

>Mbr_St38

RKKALEDRIDISEEDKNAIRREFNARETEFLRLRRSKITIQNFEFLKTIGRGAFGEVKLA

QKKDNGQIYAIKILRKADMLEKDQVAHVRAERDILVVASSDWVVK-YAFQDRINLYLIME

FLPGGDMMTMLIRYETFSEETTRFYIGEAVAAINSIHDLGFIHRDIKPDNLLLTADGHIK

LSDFGLCTGLKKAHRALAYSTVGTPDYIAPEVFSHHGYTKTCDWWSLGIIMFEMLVGYPP

FCSETPQETYRKVMNWQETLVFPPESEEAEAMIRRFCSDASVRIGGVEEIKTHPFLVNWL

RKPPIDPQVERIDDTKNFDFSGYTFKRF

>CowSt38

RRTKLEADLI---DIRSEKRRQLMTKETEFLRLKRCRLGSEDFATLKVIGRGAFGEVRLA

QKIDTGHIYAMKVLRKEDMLKKEQVAHVRAERDILAEADNPWVVQFYSFQDASSLYLVME

FLSGGDMMTMLMRYDTFSEDVTRFYVAESVAAINSIHKLNFIHRDIKPDNLLLDPKGHIK

LSDFGLCTGLKKSHRALAYSTVGTPDYIAPEVFLQTGYTKSCDWWSLGVIMFEMLIGYPP

FCSETAQETYRKIMSWRTSLIFPPESREAQDLITRLCTDADRRIEDVAEIMAHPFFVNWD

SPAPIDPGVRSIADTSNFDFLNYTFKRF

>Aqu_St38

RWKKLEQSMELADEEREERRKQHAQKETQFLRLRRSRLGKKDFKRLKIIGRGAFGEVVLV

QKIDTGHVYAMKVLRKSDMVEKEQIAHARAERDILVEADNPWVVK-YSFQDAINLYFIME

FLPGGDMMTLLMKKDILSEDVTRFYIAESILAVDSIHKMSFIHRDIKPDNLLLDSRGHIK

LSDFGLCTGLKIAHRDRAFSTVGTPDYIAPEVFMQTGYTHLCDYWSLGVIMYEMLMGFPP

FCSEKPQDTYRKIMNWRQHLIFSPESKDAESLIRSLLCDSQHRLGDVEEIKRQPFFTDWI

RPASIPVHVKHMADTSNFDFQNFTFKRF

>SciSt38

RLRNLDRQLAITEDERTSRRLQQAVRETEFLRLKRARLGPNDFESLKVIGRGAFGEVRLV

QKNDTGHVYAMKTLRKSDMLAKEQVAHVRAERDVLVEADNPWVVKYFSFQDQVNLYLIME

FLPGGDMMTLLIKQDTLSEPVTRFYAAEAALAIDSIHRLGVIHRDIKPDNLLLDSRGHLK

LSDFGLCTGLKKAHRALVNSTVGTPDYIAPEVFMQQDYSKTCDWWSLGVVMYEMLIGYPP

FCSEDAKETYRKVMHHRTTLVFPVESPDAESLIRGLICDADNRLGGLHELQAHPFFIDWE

RPAALGVEVKSIDDTSNFDFSNYTFKRF

>Ppi_St38

RLRGLEKKMRLNEEEREDQRRRQALKETDFLRLKRSRLGVGDFESLKVIGRGAFGEVRLV

QKVDTGQMYAMKIMRKVDMLEKEQVAHVRAERDVLAEADNPWVVK-YSFQDSTNLYLIME

FLPGGDMMTLLMKRDTLSEDESRFYMAECVLAIDSIHRLGFIHRDIKPDNLLLDAGGHIK

LSDFGLATGLKKAHRQLAYSTVGTPDYIAPEVFLHKGYTGMCDYWSLGVIMYEMLIGYPP

FCSENAQDTYRKVMTWKENLIYPPESNNARSLISRLVCNVDDRLGGVHEIKEHAFLADFI

RPPAISIRVRAIDDTSNFDFMNYTFKRF

>MleSt38

RLKSLEKKMRLEEDEREDQRRRQATKETDFLRLKRSRLGVDDFESLKVIGRGAFGEVRLV

QKIDTGHIYAMKIMRKVDMLEKEQVAHVRAERDVLAEADNPWVVK-YSFQDSTNLYLIME

YLPGGDMMTLLMKRDTLSEDETRFYMAECVLAIESIHRLGFIHRDIKPDNLLLDAGGHIK

LSDFGLATGLKKAHRQLAYSTVGTPDYIAPEVFLHKGYTGMCDYWSLGVIMYEMLIGYPP

FCSENAQDTYRKVMTWKENLIFPPESNNARSLISRLVCNVDDRLGGVEEIKEHQFLHDWI

RPPAIIIRVKSIDDTSNFD-----FKRF

>Tad_St38

RQETLEKAMQLPIEEREDKRRQLAAKETEFLRLKRARLSTEDFEPLKIIGRGAFGEVRLV

QKKDTGHIYAMKILRKADMLEKEQVAHVRAERDVLVEADHAWVVK-YSFQDAENLYLIME

FLAGGDLMTLLMKRDTLSENEARFLIAESVLAINSVHELGFIHRDIKPDNLLLDSKGHIK

LSDFGLCTGLKNSHRAMAYSTVGTPDYIAPEVFLQTGYTKSCDWWSLGVIMYEMLIGYPP

FCSETPKETYHKILNWRSALIFPPESDKARDLVKRLCCDAEFRIGGVGEIKQHSFFIDWI

RPSAIRVNIKSFDDTSNFDFINYTFKRF

>Hma_St38-2

QVKQLEECMGLTEKEKESRRTVYALRETEFLRLKRTRIGREDFESLKVIGRGAFGEVRLV

QKVDTGHVYAMKILHKKDMLEKEQKGVVRASF-IFCPVCKQWVTR-REIENEIQINSVEN

VECVGDLMTLLIRKDTFTESQTQFYMTESILAINFIHSLGFIHRDIKPDNLLLDARGHIK

LSDFGLCTGLKKAHRQMAYSTVGTPDYIAPEVFTQGGYEKSCDWWSLGVIMFEMLIGYPP

FCSETPQETYRKVLNWRETLVFPIETNTAKNLILKYFSFWKN--EGIVEIQSHPFFVDWI

RPAAIPVEVKSIDDTSNFDFLNYTFKRF

>Che_St38

RVKQLEEAMGLDDKDKETRRALYALRETEFLRLKRTRIGREDFESLKVIGRGAFGEVRLV

QKKDTGHVYAMKILRKKEMLAKEQVAHVRAERDILAESDNPWITK-YSFQDQTCLYLVME

FLPGGDLMTLLIRKDTFTEAQTQFYMAESILAINFIHSLGFIHRDIKPDNLLLDARGHIK

LSDFGLCTGLKKAHRQMAYSTVGTPDYIAPEVFTQSGYEKSCDWWSLGVIMFEMLIGYPP

FCSETPQETYRKVMNWRETLVFPVETNTSKNLILSFCTDADRRLGSIDDIQTHPFFVDWT

RPAAIPIEVKSIDDTSNFD---------

>Hma_St38-1

QVKQLEECMGLTEKEKESRRTVYALRETEFLRLKRTRIGREDFESLKVIGRGAFGEVRLV

QKVDTGHVYAMKILHKKDMLEKEQVAHVRAERDILAESDNSWVVK-YSFQDVSCLYLVME

FLPGGDLMTLLIRKDTFTESQTQFYMTESILAINFIHSLGFIHRDIKPDNLLLDARGHIK

LSDFGLCTGLKKAHRQMAYSTVGTPDYIAPEVFTQGGYEKSCDWWSLGVIMFEMLIGYPP

FCSETPQETYRKVLNWRETLVFPIETNTAKNLILNFCTDADHRLGGIVEIQSHPFFVDWI

RPAAIPVEVKSIDDTSNFDFLNYTFKRF

>CelSt38

-----------------------------------------DFESLKVIGRGAFGEVRLV

QKHDTGHIYAMKILRKSEMVEKEQTAHVRAERDILSEADCDWVVK-MSFQDYSNLYLVME

FLPGGDMMTLLIKKDTLTEEATQFYIAEAALAIQFIHSLGFIHRDIKPDNLLLDARGHVK

LSDFGLCTGLKKFHRAYAYSTVGTPDYIAPEVFQPNGYTKSCDWWSLGVIMYEMLIGYPP

FCSELPQETYRKVINWQQTLVFPSDSIEAKATIKRFCCEAERRLGGLDEIKQCPFVKDWI

RPPPIRVTVKSIDDTSNFDF-----KRF

>Nve_St38

RLKLLEQSMGLSEAEKEERRKLHAQKETEFLRLKRSRIGKEDFDSLKVIGRGAFGEVRLV

QKQDTGHVYAMKILRKADMLEKEQVAHARAERDILAEAENQWVVK-YSFQDDYYLFLVME

FLPGGDLMTLLMKKDTFTEEETRFYIAEALLAIDSIHQLGFIHRDIKPDNLLLDSRGHIK

LADFGLCTGLKKAHRQLAYSTVGTPDYIAPEVFIQQGYTKSCDFWSLGVIMYEMLIGYPP

FCSESPQETYKKVMNWRETLVFPPESPNARDLISR------QRLSNIEDIRAQPFFVDWL

RPAALPIQVKSIDDTSNFDFLNYTFKRF

>Cin_St38

RMQVLEKSMGLNEDQCEKKRMQHAQKETEFLRLKRTRLGLGDFEMLKVIGRGAFGEVRLA

QKKDTGHIYAMKMLRKKDMMEKEQVAHVRAERDILVEAENPWVVK-YSFQDLYNLYLIME

FLPGGDMMTLLMNKDTLTEEQTQFYIAEAVLAINSIHELGFIHRDIKPDNLLIDARGHIK

LSDFGLCTGLKKAHRKLAYSTVGTPDYIAPEVFQQSGYNLSCDWWSLGVIMYESLIGYPP

FCSESPQETYRKVMNWRNTLVFPDESDIARNLILSLCTDPEKRLGEVDSLKEHKFFVDWI

RPAAITMSIKSLTDTSNFDFINYTYKRF

>Spu_St38

RQKKLEDAIGLPEEDKREKRTQHAQKETEFLRLKRSRLGCDDFESIKVIGRGAFGEVRLV

QKKDTGHIYAMKILRKCDMHEKEQVAHVRAERDILVEADNPWVVK-YSFQDPYNLYLIME

FLPGGDMMTLLMKRETLSEEVTLFYIAETIMAINSIHKLNFIHRDIKPDNLLLDARGHIK

LSDFGLCTGLKKSHRALAYSTVGTPDYIAPEVFLQTGYSHVCDWWSLGVVMYEMLIGYPP

FCSETPQETYRKVMHWRETLQFPAESNEAKGMIQRFCCEADRRVGGVDEIKSHAFFVDWI

RPAAIPTNIKSFEDTSNFDFINYTFKRF

>Dme_St38

RLAKLEAQLSLSEAQRQEKRLQHAQKETEYLRLKRLRLGVEDFEALKVIGRGAFGEVRLV

QKKDTGHVYAMKVLRKADMLEKEQVAHVRAERDVLVEADHQWVVK-YSFQDPVNLYLIME

FLPGGDMMTLLMKKDTLSEEGTQFYISETALAIDSIHKLGFIHRDIKPDNLLLDARGHLK

LSDFGLCTGLKKSHRALAYSTVGTPDYIAPEVFLQTGYGPACDWWSLGVIMYEMLMGYPP

FCSDNPQDTYRKVMNWRETLIFPPESEEAKETIINFCCEADRRLGGLEDLKSVPFFVDWI

RPAAIPVEVRSIDDTSNFDFINYTYKRF

>Bfl_St38

--------------------------------------------------------VRLV

QKKDTGHIFAMKVLRKADMLEKEQVAHVRAERDILVEADNPWVVR-YSFQDPINLYLIME

FLPGGDMMTLLMKKDTLSEEATQFYITETALAIDSIHQLGFIHRDIKPDNLLLDSRGHTR

QSDAGMCSSLTSSHRQLAYSTVGTPDYIAPEVFMQTGYTKTCDWWSLGVIMYEMLIGYPP

FCSENPQETYRKVMNWKEMLVFPPESFNSRNLIER-------------------------

----------------------------

>Dre_St38-2

RQQKLEKVMGLADEEKRLRRSEHARKETEFLRLKRTRLGLEDFESLKVIGRGAFGEVRLV

QKKDTGHVYAMKILRKADMLEKEQVGHIRAERDILVEADSLWVVK-YSFQDKMNLYLIME

FLPGGDMMTLLMKKDTLTEEATQFYIAETVLAIDSIHQLGFIHRDIKPDNLLLDSRGHVK

LSDFGLCTGLKKAHRQLAFSTVGTPDYIAPEVFMQNGYNKLCDWWSLGVIMYEMLIGYPP

FCSETPQETYRKVMNWRETLIFPPESEKAKDLILRFCCEGEHRIGGVEEIKTNAFFVDYI

RPAAIPIEIKSIDDTSNFDFINYTYKRF

>Hsa_St38-2

RQKKLEKVMGLKDEEKRLRRSAHARKETEFLRLKRTRLGLEDFESLKVIGRGAFGEVRLV

QKKDTGHVYAMKILRKADMLEKEQVGHIRAERDILVEADSLWVVK-YSFQDKLNLYLIME

FLPGGDMMTLLMKKDTLTEEETQFYIAETVLAIDSIHQLGFIHRDIKPDNLLLDSKGHVK

LSDFGLCTGLKKAHRQLAFSTVGTPDYIAPEVFMQTGYNKLCDWWSLGVIMYEMLIGYPP

FCSETPQETYKKVMNWKETLTFPPESEKAKDLILRFCCEWEHRIGGVEEIKSNSFFVDWI

RPAAISIEIKSIDDTSNFDFINYTYKRF

>Xtr_St38-2

RQKRLEKAMGLRDEEKRMRRSAHARKETEFLRLKRTRLGLEDFESLKVIGRGAFGEVRLV

QKKDTGHVYAMKILRKTDMLEKEQVGHIRAERDILVEADSLWVVK-YSFQDKLNLYLIME

FLPGGDMMTLLMKKDTLTEEETQFYIAETVLAIDSIHQLGFIHRDIKPDNLLLDSKGHVK

LSDFGLCTGLKKAHRQLAFSTVGTPDYIAPEVFLQTGYNKLCDWWSLGVIMYEMLIGYPP

FCSETPQETYKKVMNWKETLIFPPESEKAKDLILRFCCEWEQRVGGVEEIKTNHFFVDWI

RPAAIPIEIKSIDDTSNFDFINYTYKRF

>Dre_St38-1

RQKKLEKVMGLPDEEKSMRRSLHARKETEFLRLKRTRLGLDDFESLKVIGRGAFGEVRLV

QKKDTGHIYAMKILRKADMLEKEQVAHIRAERDILVEADGAWVVK-YSFQDKRNLYLIME

FLPGGDMMTLLMKKDTLSEEATQFYIAETVLAIDSIHQLGFIHRDIKPDNLLLDSRGHVK

LSDFGLCTGLKKAHRQLAYSTVGTPDYIAPEVFMQTGYNKLCDWWSLGVIMYEMLIGYPP

FCSETPQETYRKVMNWRETLTFPPESERAKELILRYCTDAENRIGSVDEIKSHPFFVDWI

RPAAISIDIKSIDDTSNFDFLNYTYKRF

>Hsa_St38-1

RQKKLEVAMGLADEEKKLRRSQHARKETEFLRLKRTRLGLDDFESLKVIGRGAFGEVRLV

QKKDTGHIYAMKILRKSDMLEKEQVAHIRAERDILVEADGAWVVK-YSFQDKRNLYLIME

FLPGGDMMTLLMKKDTLTEEETQFYISETVLAIDAIHQLGFIHRDIKPDNLLLDAKGHVK

LSDFGLCTGLKKAHRQLAYSTVGTPDYIAPEVFMQTGYNKLCDWWSLGVIMYEMLIGYPP

FCSETPQETYRKVMNWKETLVFPPESEKAKDLILRFCIDSENRIGGVEEIKGHPFFVDWI

RPAAIPIEIKSIDDTSNFDFLNYTYKRF

>Xtr_St38-1

RQKKLEVAMGLGDDEKKMRRSQHARKETEFLRLKRTRLGLEDFESLKVIGRGAFGEVRLV

QKKDTGHIYAMKILRKADMLEKEQVAHIRAERDILVEADGAWVVK-YSFQDKRNLYLIME

FLPGGDMMTLLMKKDTLTEEATQFYIAETVLAIDAIHQLGFIHRDIKPDNLLLDAKGHVK

LSDFGLCTGLKKAHRQLAYSTVGTPDYIAPEVFMQTGYNKLCDWWSLGVIMYEMLIGYPP

FCSETPQETYRKVMNWKETLVFPPESEKSKDLILRFCADSENRVGGVEEIKSHPFFVDWI

RPAAIPIEIKSIDDTSNFDFLNYTYKRF

>CelWts

RMKQLEKEMQLPDIMRNKMLGLLQQKESKYTRLRRQKMSKSHFTVISHIGVGAFGKVSLV

RKNDTRKVYAMKSLEKADVIMKQQAAHVKAERDILAEADSPWIVR-FSFQDDACLYFIME

YVPGGDMMTLLIQKGIFEEDLARFYIAELACAIEYVHNVGFIHRDLKPDNILIDQHGHIK

LTDFGLCTGLRWTHRITAHSLVGTGNYMAPEVIAKTGHNQSCDWWSTGVILYEMVFGRVP

FHDDTPGGTQHRIKNWRNFLDFTYCSKECLMMIQQLICDASSRLGGKDQVKNHPWFRDWL

RRADYIPRVTHDEDTSNFE-----FRHF

>Cow2

RRDQLEIEMALTDVQKTQLRRILRMKESEYIRLKRVKLDKSMFTTVKKIGVGAFGEVTLV

RKVDDAHVYAMKTLRKADVLRKHQLAHVKAERDILAEADNEWVVKFYSFQDETKLYLVME

YVPGGDMMSLLMKLNTFSEDMSRFYIAEMVMAIDSVHKMGFSHRDIKPDNILIDRRGHIK

LTDFGLSTGFRSTHRSQAHSLVGTPNYIAPEILQRTGYGKECDWWSMGIILFEMLMGYAP

FCSQTSAETKRKVLNWRATLQIPPRSRESKDLISRLCCESSDRLETVEHIKMHPFFIDWT

LPAPFVPDVRTEEDTSNFDFVEFTFRRF

>Ppi_Wts

RREDFEAGIDYPEHEKDKLRHVFAQKESAFLRLKRTKINKDMFNKIKTIGVGSFGEVSLV

EKTDTGQLYAMKTLRKAEVWRRNQTAHVKAERDILSEADNEWVVK-FSFQDKVNLYFVME

YVSGGDFMNLLIKSGRIPEWASRFYIAELVCAVESVHKLGFIHRDIKPDNILIDERGHLK

LTDFGLCTGFRWTHRCLAHSLVGTPNYIAPEVLLREWYQHECDWWSVGVILYEMILGKPP

FLARTESDINYRIINWRDHLYIDKESDDAISLIFGLLQDSHMRLGGACEIKDHP------

----------------------------

>MleWts

RRDEFEASIDYPDNEKERLRMLFAQKESDFLRLKRSKINKDMFTKIKTIGIGSFGEVSLV

QKTDTGTLYAMKTLRKAEVWKRNQTAHVKAERDILSEADNKWVVK-FSFQDKGNLYFVMD

YVPGGDMMSLLIKYGRFPEWAACFYIAELVCAVDSVHKLGFIHRDIKPDNILIDERGHLK

LTDFGLCTGFHWTHRCLAHSLVGTPNYIAPEVLLREWYQHECDWWSVGVILYEMIIGKPP

FYANTAADIQMSIINWRCCLSIPTASEHSINLIFDLLQDSDKRLGGAEEIKEHPFFATWL

HRAPMIPVLIFITLQPEFD------TRG

>SciWts

RRMQLEREMGLGEDTREQMLGILHVKESNYLRVKRSKMDRSQFEKLSTLGVGSFGEVALV

RKVDQRKLYAMKTLKKKVVLKRNQAAHVKAERDLLAEADNEWVVKFYSFQDRENLYFVME

YIPGGDMMSLLIKLGMFPEDLARFYIAELVLAVDSVHSVGFVHRDIKPDNILIGADGHIK

LTDFGLCTGFRWTHRCMAHSLVGTPNYIAPEILGRRPYGKLVDWWSVGVILYEMIVGRPP

FLASSPMETQLKVMDYKRTLKVPRSSSAADDLLRQLLCDPVKRLGKVDDLKHHPFFINWA

YAAPYIPTIRHSTDTSNFDFYEFTFRRF

>Cin_Wts

RRWQLEKEMNLPEETKTGMRQMLFKKESNYIRLKRAKMDLSMYDMIKSIGVGAFGKVMLA

RNKTSDKLNAIKFLKKKDVLRRNQVAHVKAERDILSEADNDWVVK-YTFQDKENLYFVMD

YIPGGDLMSLLIKKEIFDQTLARFYTAELTLALESVHKMGFIHRDIKPDNILIDRDGHIK

LTDFGLCTGFRWTHRHVARSLVGTPNYIAPEVLLREGYTQMCDWWSVGVILYEMVVGSPP

FHSDTPSETQLKVINYKKSLRIPHHAPQTEDIIRRFCCDQGARIGGAEEVKKHEFFITWL

RKAPYIPEIKFSEDTSNFDFFEFTFRRF

>Aqu_Wts

RRMKLEVEMGLDEATCEQMRKLLAQKESNFLRMKRAKMHRDQFDIVKPIGVGAFGVVSLV

RKKDTRRLYAMKSLRKADVVRRNQVAHVKAERDILAEADNEWVVK-YSFQDAECLYFVME

YIPGGDMMSLLIKLGTFPEHLTLFYIAELVCAIESVHKMGFIHRDIKPDNILIEADGHIK

LTDFGLCTGFRWTHRCVAHSLVGTPNYIAPEILRRVGYTQLCDWWSVGVIMYEMLVGQPP

FMASAPADTQLKIVNWPEYLRIPHHSTAARDLVLRFLSDPQDRIGGADEIKHHPFFIDWI

RVAPYKPKIMYEGDTSNFDFYEFTFIRF

>Hma_Wts

RRKQLEDEMGLPLQDQEQMRKLLRQKESNYIRLRRAKMDKTMFTKIKIIGIGAFGEVSLV

RKNGTEAYYAMKTLRKSEVVRRNQVAHVKAERDILAEADNEWVVK-YSFQDTNNLYFVMD

YVPGGDLMALLIKRGIFEDNLARFYIGELVLAIESVHKLGFIHRDIKPDNVLIDRYGHIK

LTDFGLCTGFHWTHRCQAHSLVGTPNYIAPEVLMRIPYSQQCDWWSVGVILYEMLIGHPP

FMARTPAEIQLKIINWKETLTIRPR--HSENLILQLCSAPENRIGGAHEIKNHPYFFNFI

HKAPFVPIINHPTDTSNFDFYEFTFRHF

>Che_Wts

RRQQLEAEMRLPIQAQEQMRKLLNQKESNYIRLRRAKMDKSMFTKIKVVGVGAFGEVSLV

RKKETNAYYAMKTLRKSEVLRRNQVAHVKAERDILAEADNEWVVK-YSFQNQQYLYFVMD

YVPGGDLMALLIKFGIFNEPLARFYISELVLAIESVHKLGFIHRDIKPDN----------

------CTGFRWTHRCQAHSLVGTPNYIAPEVLMRIPYGQQCDWWSVGVIMYEMLIGQPP

FLAQSPAETQFKVINWRQILNIPRE-PEAEDLIRKLCSDPNERIGDAEQIKLHPYFVDFL

HKAPHVPTIKNATDTSNFDFYEFTFRHF

>Lgi_Wts

RRFQLENEMGLSDEAATQMRRMLHQKESNYIRLKRAKMDKLMFDKIITLGVGAFGEVSLV

RKRDPKTLYAMKTLRKSDVIKRNQVAHVKAERDILAEADNEWVVK-YSFQDRDHLYFVMD

YVPGGDLMGLLIKMEILDETLARFYIAELVLAIESVHKMGFIHRDIKPDNILIDHDGHIK

LTDFGLCTGFRWTHRCLAHSLVGTPNYIAPEVLLRQGYSSVCDWWSVGVILYEMLLGHPP

FYASTPIETQTKVIHWKQTLKIPSDSHESKDLILRLLCSAEDRLGGAQEVKNHAFFIHLL

RAAPFIPTIRYPTDTSNFDFYEFTFRRF

>Nve_Wts

--------MGLSEEAQSQMRQLLKQKETNYIRLKRTKMDISMFEKIRTIGIGAFGEVWLV

RKTDTSMLYAMKILRKSEVFRRNQAAHVKAERDILAEADNEWVVK-YSFQDQENLYFVMD

YVPGGDLMSLLIKFGIFPEDYAKFYIAELVLAIDSVHRMGFVHRDIKPDNILIDKDGHIK

LTDFGLCTGFRWTHRCQAHSLVGTPNYIAPEVLLRIGYRQSCDWWSVGVILYEMLVGQPP

FLAPTPAETQLKIINWETELRIPHQSREAKDLILRLCTNADMRLGGASEIKAHPFFVDWL

AQAPYTPVIRFPTDTANFDFFEFTFRRF

>Spu_Wts

RRMQLEQEMDLTSGDRDQMRRMLFQKESNYLRLKRAKMNKSMFKKIKTLGIGAFGEVALA

RKVDTNALYAVKKLRKLDVIQRNQAGHVKAERDILAEADNEWVVK-YSFQDKDNLYFVMD

YIPGGDLMSLLIRLEIFEEDLARFYIAELVLAIESVHKMDFIHRDIKPDNILVDRDGHIK

LTDFGLCTGFRWTHRCKAHSLVGTPNYIAPEVLTRTGYTQLCDWWSVGVILYEMLVGQPP

FHADSPAETQWKVINWKQCLHIPSQSRDASDFILRLCTGPEERLGGVLDIKSHPFFIDLI

RKAPYIPTVRHPTDTSNFDFFEFTFRRF

>Dme_Wts

RKNQLEKEMGLPDQTQIEMRKMLNQKESNYIRLKRAKMDKSMFVKLKPIGVGAFGEVTLV

SKIDTNHLYAMKTLRKADVLKRNQVAHVKAERDILAEADNNWVVK-YSFQDKDNLYFVMD

YIPGGDLMSLLIKLGIFEEELARFYIAEVTCAVDSVHKMGFIHRDIKPDNILIDRDGHIK

LTDFGLCTGFRWTHRVLAHSLVGTPNYIAPEVLERSGYTQLCDYWSVGVILYEMLVGQPP

FLANSPLETQQKVINWEKTLHIPPQSREATDLIRRLCASADKRLG-VDEVKSHDFFIDFM

RKAPYIPEIKHPTDTSNFDFFEFTFRRF

>Bfl_Wts

--------MGLSEEAQSQMRKMLCQKESNYIRLKRAKLDKSMFVKIKTLGVGAFGEVALA

RKVDTQTLHAMKTLRKSDVLKRNQVAHVKAERDILSEADNEWVVR-YSFQDDENLYFVMD

YIPGGDLMSLLIKKGIFEQGLAQFYTAELVCAIESVHKMGFIHRDIKPDNILIDRDGHIK

LTDFGLCTGFRWTHRCQAHSLVGTPNYIAPEVLLRRGYTQLCDWWSVGVILYEMLVGQPP

FLATTPAETQMKVINWDHYLAIPAQSRAASDIILKFCCDAENRLGGVQEIKAHPFFIPWL

RKAPYAPYIRYPTDTSNFDFYEFTFRRF

>Dre_Wts

RKKQLESEMGLSGDAQEQMRMMLSQKESNYIRLKRAKMDKCMFEKIKTLGIGAFGEVCLA

RKVDTGALYAMKTLRKKDVLLRNQVAHVKAERDILAEADNEWVVR-YSFQDKDNLYFVMD

YIPGGDMMSLLIRMGIFQEDLAQFYIAELICAVESVHKMGFIHRDIKPDNILIDRDGHIK

LTDFGLCTGFRWTHRCLAHSLVGTPNYIAPEVLLRTGYTQLCDWWSVGVILYEMVVGQPP

FLATTPLETQMKVIRWQTSLHIPLQSPEATDLILKLCRGPDDRLGGADEIKAQPFFIDLL

RQAPYIPKITHSTDTSNFDFYEFTFRRF

>Hsa_Wts

RKKQLENEMGLSQDAQDQMRKMLCQKESNYIRLKRAKMDKSMFVKIKTLGIGAFGEVCLA

RKVDTKALYATKTLRKKDVLLRNQVAHVKAERDILAEADNEWVVR-YSFQDKDNLYFVMD

YIPGGDMMSLLIRMGIFPESLARFYIAELTCAVESVHKMGFIHRDIKPDNILIDRDGHIK

LTDFGLCTGFRWTHRCLAHSLVGTPNYIAPEVLLRTGYTQLCDWWSVGVILFEMLVGQPP

FLAQTPLETQMKVINWQTSLHIPPQSPEASDLIIKLCRGPEDRLGGADEIKAHPFFIDFL

RSASYIPKITHPTDTSNFDFYEFTFRRF

>Xtr_Wts

RKKQLENEMGLSQEAQDQMRKMLCQKESNYIRLKRAKMEKSMFVKIKTLGIGAFGEVCLA

RKTDTNALYAMKTLRKKDVLLRNQVAHVKAERDILAEADNEWVVR-YSFQDKDNLYFVMD

YIPGGDMMSLLIRMGVFPEDLARFYIAELTCAVESVHKMDFIHRDIKPDNILIDRDGHIK

LTDFGLCTGFRWTHRCLAHSLVGTPNYIAPEVLLRTGYTQLCDWWSVGVILYEMLVGQPP

FLAQSPVETQMGI---------PHA------------------FEG-------PFF----

----------------------------

>Dre_Wts2

RRMQLEQEMGLSEAEQEQMRKMLYQKESNYNRLRRAKMDKSMFVKIKTLGIGAFGEVCLT

RKVDTGALYAMKTLRKKDVLNRNQVAHVKAERDILAEADNEWVVR-YSFQDRDNLYFVMD

YIPGGDMMSLLIRMGVFPEVLARFYVAELTLAIESVHKMGFIHRDIKPDNILIDLDGHIK

LTDFGLCTGFRWTHRCLAHSLVGTPNYIAPEVLLRKGYTQLCDWWSVGVILFEMLVGQPP

FLAPTPTEAQLKVINWENTLQVPPQSPEAVDIIGQLCCSAEERLGGAGEIKAHPFFVDFL

RPAPYRPKIAHPMDTSNFDFYEFTFRRF

>Hsa_Wts2

RRLQLEQEMGLCEAEQEQMRKILYQKESNYNRLKRAKMDKSMFVKIKTLGIGAFGEVCLA

CKVDTHALYAMKTLRKKDVLNRNQVAHVKAERDILAEADNEWVVK-YSFQDKDSLYFVMD

YIPGGDMMSLLIRMEVFPEHLARFYIAELTLAIESVHKMGFIHRDIKPDNILIDLDGHIK

LTDFGLCTGFRWTHRCLAHSLVGTPNYIAPEVLLRKGYTQLCDWWSVGVILFEMLVGQPP

FLAPTPTETQLKVINWENTLHIPAQSPEARDLITKLCCSADHRLGGADDLKAHPFFIDFI

RPAPYVPTISHPMDTSNFDFYEFTFRRF

**Mats**

>Che_Mats1

TFRPKKSAPSGSKGAQLRKHIDATLGSGLREAVKLPTGEDLNEWLAVVDFFNQVNLLFGT

LTEFCTPENCPTMTAGPKYEYRWADGKKPIEVSAPKYVEYLMDWIESQLDDESIFPQKLG

APFPPNFREVVKTIFKRLFRVYAHIYHSHFQKIVSLKEEAHLNTCFKHFILFTCEFALID

KKELAPL

>Ppi_Mats

TFKPKKNLPDGQH----LKDAEATLGSGLSQAVKLPPGEDLCEWVAVVDFYNQINMLYGT

IQDYCTEESCPIMTAGPKYEYHWSDG-KPIKCSAPKYIDELMTWVQDQLEDDTLFPDKVG

VAFPKNFMTMCKNILKKLFRVYAHIYHLHFKIIIELGEEPHLNTSLKHFVYFCNEFNLIE

RKQYAPL

>Mle_Mats

TFKPKKNLPDGQH----LKDAEATLGSGLGLAVKLPDGEDLCEWVAVVDFYNQINMLYGT

IQDYCTEESCSIMTAGPKYEYHWSDG-KPIKCSAPKYIDLLMTWVQEQLEDEKVFPDKIG

VAFPKNFLSICKNILKKLFRVYAHIYHCHFSNVIDLGEEPHLNTSLKHFVYFCNEFHLIE

EKQYAPL

>SciMats2

----------------------------------------------------QINMLYGS

VSEHCTAESCPIMSAGPRFEYHWADGKKPIKCSAPKYMDYLMSWVQEQLDDESIFPSKVG

VPFPKNFLSTAKTILKRLFRVYAHVYHQHFQSVIHLGEEAHLNTSFKHFNFFIAEFNLVE

KRELQPL

>Mbr_Mats

TFKPKKNIPEGTKQHDLKKYVDATLGAGLRAAVRLPEGEDLDEWIAVVDFFNQINILYGT

ITEFCTRESCPIMSAGPKYEYQWADGKRPIRCSAPEYIDFLMTWVQAQLDDESIFPSKIG

APFPKDFLVLVKAILKRLYRVYAHMYHSHFNKIVALGEEPHLNTSFKHFIYFVKEFNLVE

PRELAPM

>Aqu_Mats

TVKQKR-LPDGSAGHDLLKHAAQTLGSGIKEAVKLPDGEDLNEWIAVVDFFNQINMLYGT

ITEKCTSESCPVMSAGPKFEYHWADGKKPIKCSAPKYIDYLMTWVQEQLDDEAIFPSRTG

VDFPRNFITVAKTILKRLFRVYAHVYHAHFEDILSLKEEAHLNTSFKHLMYFVHEFNLID

KRELTPM

>SciMats3

TFRPKKNIPEGTHQYELMQHAAATLGSGLREAVALPDGEDVNEWVATVDFFNQINMLYGT

IAEHCTPESCPVMSAGPRYEYHWADGKKPVKCSAPKYMDYLMSWIQEQLDDESIFPSKVG

VAFPKSFMNVAKTIFKRLFRVYGHIYHQHFQSVISLGEEAHLNTSFKHYILFVQEFNLID

KRELQPL

>Cin_Mats

TFKPHKSIPEGSHQHELIRHAAATLGSGLQLAVALPEGEDLNEWIAVVDFFNQINMLYGT

ISEFCTATKCEVMSAGPKYEYHWADGKKPIKCSAPRYIDYLMTWVQCQLDNEEIFPSAVG

VPFSKNFMSIAKTILKRLFRVYAHIYHQHFSDVMGLGEEAHLNTSFKHFIYFVQEFNLID

RRELAPL

>Tad_Mats

TFKPKKNIPEGTHQHDLMKHAAATLGSGLRLAVVLPEGEDLNEWVAVVDFFNQINMLYGT

ITEFCTEITCPVMSAGPKYEYHWADGKKPIKCSAPRYIDYLMSWVQEQMDDEVIFPSKIG

VPFPKNFIMVAKTILKRLFRVYAHIYYNHFNQIMGLGEEAHLNTSFKHFVYFVQEFSLVD

RRELAPL

>Cow1

TFKPKRNIPEGTKQYQLKKYAEATLGSGLRLAVTLPEGEDLNEWVATVDFFNQINMLYGT

ITEFCTAEECPVMSAGPKYEYHWADGKKPIKCSAPEYIDFLMTWVQGQLDDETIFPSKIG

VPFPKSFQATAKNILKRLFRVYAHIYHSHFNKIVSLGEEAHLNTSFKHFIFFVQEFNLIE

KKELAPL

>Che_Mats2

TFRPRRSIPEGTHQYDLLKHAEATLGSGLRNAVMLPEGEDLNEWVAVVDFFNQINMLYGT

ITEECTPESCPVMSAGPKFEYLWADGKKPVKCSAPQYIDYLMQWVQDQLDDETLFPSKIG

VPFPKNFLSIAKTMLKRLFRVYAHI-----------------------------------

-------

>Spu_Mats

TFRPKKNIPEGFHQYELMKHAEATLGSGLRQAVSLPDGEDINEWVAVVDFFNQINMLYGT

ITEFCTTDKCPVMSAGPKYEYHWADGKKPIKCSAPKYIDYLMTWVQDQLDDETIFPSKIG

VPFPKNFMTIAKTILKRLFRVYAHIYHQHFKEIVILAEEAHLNTSFKHFIYFVQEFNLID

RKELAPL

>Hma_Mats

-------------------------------------GEDLNEWIAVVDFFNQINMLFGT

ITDHCTSESCPVMSAGPKFEYLWADGKKPIKCSAPKYIDYLMTWVQDQLDNEALFPSKIG

VPFPKNFVSIAKTILKRLFRVYAHIYHQHFPQVVSLGEEAHLNTSFKHFIYFVQEFGLID

KRELAPL

>Xtr_MOB1A

TFKPKKNIPEGSHQYELLKHAEATLGSGLRQAVMLPEGEDLNEWIAVVDFFNQINMLYGT

ITEFCTESTCSVMSAGPRYEYHWADGKKPIKCSAPKYIDYLMTWVQDQLDDETLFPSKIG

VPFPKNFMSVAKTILKRLFRVYAHIYHQHFDAVMQLQEEAHLNTSFKHFIFFVQEFNLID

RRELAPL

>Gga_MOB1A

TFKPKKNIPEGSHQYELLKHAEATLGSGLRQAVMLPEGEDLNEWIAVVDFFNQINMLYGT

ITEFCTEASCPVMFAGPRYEYHWADGKKPIKCSAPKYIDYLMTWVQDQLDDETLFPSKIG

VPFPKNFMSVAKTILKRLFRVYAHIYHQHFDSVMRLQEEAHLNTSFKHFIFFVQEFNLID

RRELAPL

>Hsa_MOB1A

TFKPKKNIPEGSHQYELLKHAEATLGSGLRQAVMLPEGEDLNEWIAVVDFFNQINMLYGT

ITEFCTEASCPVMSAGPRYEYHWADGKKPIKCSAPKYIDYLMTWVQDQLDDETLFPSKIG

VPFPKNFMSVAKTILKRLFRVYAHIYHQHFDSVMQLQEEAHLNTSFKHFIFFVQEFNLID

RRELAPL

>Xtr_MOB1B

TFKPKKSLPEGSHQYELLKHAEATLGSGLRMAVMLPEGEDLNEWVAVVDFFNQINMLYGT

ITDFCTEESCPVMSAGPKYEYHWADGKKPIKCSAPKYIDYLMTWVQDQLDDETLFPSKIG

VPFPKNFMSVAKTILKRLFRVYAHIYHQHFDSVIQLQEEAHLNTSFKHFIFFVQEFNLID

RREQAPL

>Hsa_MOB1B

TFKPKKNIPEGSHQYELLKHAEATLGSGLRMAVMLPEGEDLNEWVAVVDFFNQINMLYGT

ITDFCTEESCPVMSAGPKYEYHWADGKKPIKCSAPKYIDYLMTWVQDQLDDETLFPSKIG

VPFPKNFMSVAKTILKRLFRVYAHIYHQHFDPVIQLQEEAHLNTSFKHFIFFVQEFNLID

RRELAPL

>Gga_MOB1B

TFKPKKNIPEGSHQYELLKHAEATLGSGLRMAVMLPEGEDLNEWVAVVDFFNQINMLYGT

ITDFCTEESCPVMSAGPKYEYHWADGKKPIKCSAPKYIDYLMTWVQDQLDDETLFPSKIG

VPFPKNFMSVAKTILKRLFRVYAHIYHQHFDPVIQLQEEAHLNTSFKHFIFFVQEFNLID

RRELAPL

>Dme_Mats

TFKPKKNIPEGTHQYDLMKHAAATLGSGLRNAVALPDGEDLNEWVAVVDFFNQINMLYGT

ITEFCTEETCGIMSAGPKYEYHWADGKKPIKCSAPKYIDYLMTWVQDQLDDETLFPSKIG

VPFPKNFHSSAKTILKRLFRVYAHIYHQHFTEVVTLGEEAHLNTSFKHFIFFVQEFNLIE

RRELAPL

>Bfl_Mats

TFKPKKHIPEGTHQYDLMKHAEATLGSGLRLAVVLPEGEDLNEWVAVVDFFNQINMLYGT

ITEYCTEERCPVMSAGPKYEYHWADGKKPIKCSAPKYIDYLMTWVQDQLDDETLFPSKIG

VPFPKNFASIAKTILKRLFRVYAHIYHQHFQEVVQLGEEAHLNTSFKHFVFFVQEFNLIE

RRELAPL

>Sci_Mats1

TFKPKKNIPEGTHQYDLMKHAAQTLGSGLRLAVMLPEGEDLNEWVATVDFFNQINMLYGT

ITEFCTAENCPVMSAGPRYEYHWADGKKPIKCSAPRYTDYLMSWVQDQLDDETLFPSKIG

VPFPKSFLNSCKTILKRLFRVYAHIYHQHFREVVSLGEEAHLNTSFKHFIFFVQEFTLID

KRELQPL

>Lgi_Mats

TFKPKKNIPEGTHQYDLMKHAAVTLGSGLRLAVMLPEGEDLNEWVAVVDFFNQINMLYGT

ITEFCSEETCPVMSAGPKYEYHWADGKKPIKCSAPKYIDYLMTWVQDQLDDETIFPSKIG

VPFPKNFLTIAKTILKRLFRVYAHIYHQHFKEVVQLSEEAHLNTSFKHFIFFVQEFSLID

RKELAPL

>Nve_Mats

TFKPKKNIPEGTHQYDLMRHAAATLGSGLRLAVMLPEGEDLNEWVAVVDFFNQINMLYGT

ITEFCTLESCPVMSAGPKYEYHWADGKKPIKCSAPKYIDYLMTWVQDQLDDETLFPSKIG

VPFPKNFLAIAKTILKRLFRVYAHIYHQHFKHVVSLGEEAHLNTSFKHFIFFVQEFSLID

KRELAPL

>Mbr_2

DFSAKKNFKEGSLRHELHKKAVATLNSGLRQAVKLPEGEDMNEWLAVVEFFNRVNLVYGA

VCDFCTEESCPMMRAGPAYEYQWKDDKRPTYVSAPKYISLLMDWIEQIISDESKFPSNPE

VPFPKDFQKIIQQMFRRLFRVFAHVYYEHFNQLSEIGAEAHINTCYKHFYYFATEFNLIP

TKELEPL

>Cow3

TFRPKKKFDKGSLRYDLYKKAKASLGTGLKVAVQLPPTENLNEWLAVVDFFNRVNLIYGT

IGDQC---DCPTMSAGRQYEYSWADGKTPTSLPAAQYVALLMEWIEQQINDEAIFPSKID

TPFPKTFQQVCKNIFKRLFRVFAHVYYHHFETVQAIGAEAHVNTCFKHFYYFVKEFSLIE

PKELEPL

>Dme_MOB3

TFRPKKPFASGTIRYSLHKQAQASLQSGLRQVVRLPQGENLNDWLAVVDFFNRINLIYGT

VSEFCNETTCPTMSGGSRYEYLWADGKKPTALSAQKYIEHLMDWIETQINNEAVFPVSTD

VPFPKNFIAISRKILTRLFRVFVHVYIHHFDRIVSIGAEAHVNACYKHFYYFVQEFDMIS

AKELEPL

>Bfl_MOB3

TFRPKKKFQQGTMRYQLHKHAQATLSGGLREAVRLPHREDLNDWVAVVDFFNRINLIYGT

ISDFCTDESCPTMSGGPRYEYRWADDKKPTMVPANKYISLLMDWIESNINNENIFPLDTS

TPFPKNFLPLCKKMLTRLFRVFVHVYIHHFDRVVAIGGEPHVNTCYKHFYFFVTENNLVE

KRELEPL

>Hma_MOB3A

-----------------------------------------------VDFYNRINLIYGT

ISDVCTEASCPVMSGGPKYEYYWADGKKPTALSAPMYISTLMDWVDHQVNDENIFPPNSD

DPFPKNFLSIVKNILKRLFRVFVHVYIHHFEKIVSLGAEAHINQCYKHFYHFVTEFDLIE

KRELNPL

>Hma_MOB3B

-----------------------------------------------VDFYNRINLIYGT

ISDVCTEASCPVMSGGPKYEYYWADGKKPTALSAPMYISTLMDWVDHQVNDENIFPPNSG

MVFISSFLTM--------------------------------------------------

-------

>Nve_MOB3

------------------------------------------------------------

------------------------------------YVELLMEWTENLINNEDIFPVDKD

VPFPKQFLPVAKKILTRLFRVFVHVYIHHFDKIQSLGA----------------------

-------

>Cin_MOB3

TFRPKKHFEPGTIKFDLHKKAQASLRSGLKAIVVLPSGEDENDWIAVVDFFNRINLIYGT

VSDFCTNESCPMMSGGPKYEYRWQDNKKPTNLSASMYVAELMQWIEHLINDEAIFPTKVG

TPFPKSFKTTCKKILTRLHRVFVHVYIHHFDRVHSMGAEAHINACYKHFFYFVKCFGLVD

KKELEPL

>Spu_MOB3

TFRPKKKFESGTMRFNLHKKAQASLNSGLKVVVKLPAEEDFNDWLAVVDFFNRINLIYGT

VCEYCSADTCPIMSGGPRYEYMWMDSKKPTALPASDYINKLMDWVEQLINNENIFPINTD

VSFPKSFVSTCKKILTRLHRVFIHVYIHHFDKLVEIGAEAHINTCYKHFYFFVHEFNLVD

SKELEPL

>Xtr_MOB3B

TFRPKRKFDPGTQRFELHKRAQASLTSGLKATVQLPTGEDLNDWVAVVDFFNRINLIYGT

VCDFCTERTCPIMSGGPKYEYRWQDDKKPTALPAPQYMNLLMDWIEVQINNEDIFPTSVG

VPFPKNFLQI-----------------------------------YKHFYYFVTELNLVD

RKELEPL

>Hsa_MOB3B

TFRPKRKFEPGTQRFELHKRAQASLNSGLKAAVQLPSGEDQNDWVAVVDFFNRINLIYGT

ICEFCTERTCPVMSGGPKYEYRWQDDKKPTALPAPQYMNLLMDWIEVQINNEEIFPTCVG

VPFPKNFLQICKKILCRLFRVFVHVYIHHFDRVIVMGAEAHVNTCYKHFYYFVTEMNLID

RKELEPL

>Gga_Mats4

TFRPKRKFEPGTQRFELHKRAQATLHSGLKAAVQLPRGEDQNDWVAVVDFFNRINLIYGT

ICEFCTERTCPVMSGGPKYEYRWQDDKKPTALPAPQYMNLLMDWIEMQINNEDIFPTSVG

VPFPKNFLQICKKILCRLFRVFVHVYIHHFDRIILIGAEAHVNTCYKHFYYFVTELNLID

RKELEPL

>Hsa_MOB3A

TFRPKRKFEPGTQRFELHKKAQASLNAGLRLAVQLPPGEDLNDWVAVVDFFNRVNLIYGT

ISDGCTEQSCPVMSGGPKYEYRWQDERKPTALSAPRYMDLLMDWIEAQINNEDLFPTNVG

TPFPKNFLQTVRKILSRLFRVFVHVYIHHFDRIAQMGSEAHVNTCYKHFYYFVKEFGLID

TKELEPL

>Xtr_MOB3A

TFRPKRKFEPGTQRFELHKKAQASLNAGLKLAVQLPHGEDLNDWVAVVDFFNRINLIYGT

VSDSCTEQSCPVMSGGPKYEYRWQDDRKPTALSAPKYMNLLMDWIEVQINNEGIFPTNVG

TPFPKNFLQVVKKILSRLFRVFVHVYIHHFERIIQMGAEAHVNTCYKHFYYFVTEFNLID

TKELEPL

>Gga_Mats3

TFRPKRKFEPGTQRFELHKKAQASLNAGLKVAVQLPPGEEQNDWVAVVDFFNRINLIYGT

ISDYCTEQSCPVMSGGPRYEYRWQDERKPTALSAPQYMNLLMDWIEVQINNEDIFPTNVG

TPFPRNFLPVVKKILSRLFRVFVHVYIHHFDRITQMGSEAHVNTCYKHFYYFVKEFNLID

TKELEPL

>Hsa_MOB3C

TFRPRKRFEPGTQRFELYKKAQASLKSGLRSVVRLPPGENIDDWIAVVDFFNRINLIYGT

MAERCSETSCPVMAGGPRYEYRWQDERRPAKLSAPRYMALLMDWIEGLINDEEVFPTRVG

VPFPKNFQQVCTKILTRLFRVFVHVYIHHFDSILSMGAEAHVNTCYKHFYYFIREFSLVD

QRELEPL

>Xtr_MOB3C

TFRPRKKFEPGTQRFELYKKAQASLKSGLKTVVQLPPGENINDWIAVVDFFNRINLIYGT

MSEFCTERSCPIMCGGLKYEYRWQDDKRPTKVSAPLYMNMLMEWIETLINNEDIFPTRMG

VPFPKNFQQVCNKILTRLFRVFVHVYIHHFDAIISVGAEAHVNTCYKHFYYFITEFSLVD

HRELEPL

>Gga_Mats5

TFRPRKKFEPGTQRFELYKKAQASLKSGLKAVVQLPPGESINDWIAVVDFFNRINLIYGT

MSEYCTEKSCPIMSGGLKYEYRWQDDKKPTKLSAPQYMCMLMDWIEMLINNEDIFPTRIG

VPFPKQFQQVCTKILTRLFRVFVHVYIHHFDSIINMGAEAHVNTCYKHFYYFIREFSLVD

HRELEPL

>lgi_MOB3

TFRPKKRFDVGTMKYSLHKQANASLNAGLKEVVKLPPGEDVNDWIAVVDFFNRINLLYGT

VCENCTEQSCPTMSGGPRYEYHWCDGKRPTALPAPQYISLLMEWAESQINNENIFPVTVG

VPFPKNYLQSCKKILTRLFRVFVHVYIHHFDKLVAIGAEAHINTCYKHFYYFVTEYNLVD

KKELEPL

>Sci_MOB3

TFRPKKKFEPGTLRHDLHKKAKASLHAGLKQSVDLPPGEDPSDWIAVVDFFNRINLIYGT

VCEFCTESTCPVMSGGPKYEYMWADGKKPTKLPAPRYIHFLMEWIERQINDTDLFPIEVG

TPFPKNFLPTSKKILTRLFRVFVHVYIHHFDKLQMIGAEAHINTCFKHFVYFTRRFKLVD

EKELEPL

>Aqu_MOB3

TFRPKKKWERGTLKYELHKRAKASLNAGLKNAVALPADEDANDWIAVVDFFNRINLIYGT

VGEFCTESSCPVMSGGPKFEYYWADEKKPQKLPANQYVTKLMEWIEKQINDENIFPSQVG

TPFPKTFLATCKKILTRLYRVFVHVYIHHFDKLIAIGAEAHINTCYKHFYFFVTEFKLVD

PKEFEPL

**Scalloped**

>Cow_Sd

GRNELIARYIRMKTGKGRSRKQVSSHIQVIARKRQREIGERLK-----------------

--FVNIELNDPQMEIVEAKLIADKFP-----LRERFAQNPAGGFYLIKFWTELPGNYYWM

TARFESQEQITLEVTRSVVSFGK-------------------------PLCEYMQTFIEK

LRGLPSRELMNNVLENF---------------------FEVISDDTGSQHNVYRLV

>Sci Sd

GRNELIARYIKIRTGKIRTRKQVSSHIQVLARKRSRELQTKLKLKAELTAFKE-------

--FVHIPFHDPSLESIEIRQVCDKFP-----LSDLYEKGPPDVFFLVKFWADIDMPFY-V

STVFRTADQVKCKCTTDVCSFGQPVVQKVEVLNSTFENGAHMVRSMRSKMCDYMVKFIRK

LVALDSRAQMNEVLENFTIMQVVCNGDTGELLMCLAYAFEVSGSDSGTYHNIYRLI

>Mbr_Sd

GRNELIARYIKLKTGKTRSRKQVSSHIQVLARKKQRELQTKLKLRLYFHAFVDH------

-------FADPQMECIELSQIADKFPR----LHEAYLQGPPEAFFLVKFWVDMTFDFFGL

TCRFESLECMVVEISMCAIQLGKPVVEK-------HDQSRYVYAMNRSPLCEYMATFAQR

LR--------------------------------FACVFEVAKPGLGCGHHVYKLV

>Tad_Sd

GRNELIARYIKLRTGKHRSRKQVSSHIQVLARKKAREIKNNLK---EFSAFLTSKP---R

KHFVRIG-----IESIDIQQILDKFPDGQNGLKDLYSKGPPDAFYLVKFWGDMNITFYGV

SSCYESEKQMKILISSKVCSFGKQVVEKVEGEHGSYENGKYVYQATDSPWCDYLITFIGK

LRSLPEKYMMNSVLENFTVLQVVINQETKETLLCIAYIFEVSSGDHGTQHRVYRLV

>Dme_Sd

GRNELIARYIKLRTGKTRTRKQVSSHIQVLARRKLREIQAKIKFRLEFTAFMEIQDEIYH

RHFVQLGFSDPLLETVDIRQIFDKFPEKSGGLKDLYEKGPQNAFYLVKCWADLNTDFYGV

TSQYESNENVVLVCSTIVCSFGKQVVEKVESEYSRLENNRYVYRIQRSPMCEYMINFIQK

LKNLPERYMMNSVLENFTILQVMRARETQETLLCIAYVFEVAAQNSGTTHHIYRLI

>Aqu_Sd

GRNELIARYIKLRTGKQRTRKQVSSHIQVLARKKAREIQGKIKLRMEFYAFVDQLSEAGK

KHFVNIEFSDPSMEAVDIRHIADKFPEKNGGLKELFEKGPQDRFFLVKFWADINTPFYGV

TSQYESQENMTISCSTKVCSFGKQVVEKVETEYARFDNGRFIYRINRSPMCDYMVSFIHR

LKHLPEKYMMNSVLENFTVLQVVTNRETLETLLCLAYVFEICTSDDGPKHRVYRLV

>Aae_Sd

GRNELIARYIKLRTGKTRTRKQVSSHIQVLARRKLREFQAKMKFRLEYSAYLEMREDTYH

KHFVHIGPAHPLLESVEVKEIYDKFPQKAGGLKELYEKGPSNAFFLVKFWADLNTNFYGV

SSHYESNDNMVITCSTKVCSFGKQVVEKVETEYSRFENGRYVYRISRSPMCDYMINFINK

LKHLPEKYMMNSVLENFTILQVISNKETDETLLCVAFVFEVSTSEHGAQHHIYRLV

>Che_Sd

GRNELIARYIKLRTGKTRSRKQVSSHIQVLARRKARDIQDSQSLRLDFSAYIEIT-ESYH

KHFVNLNFEDPGIESIDIRQIYDKFPEKKGGLKELYEKGPQNLFFLVKFWADLNTN----

------------------------------------------------------------

--------------------------------------------------------

>Cta_Sd

-------------------------------------------LRIEFSAYLEQNPESYH

KHFVHIGPSDPLLEAVDIRQIYDKFPEKKGGLKELYDSGPSSAFFLVKFWADLNTSFYGV

NSHYESSENMTIQCSTKVCSFGKQVVEKVETEFGRFENGRYHFHISRSPMCEYMINFIHK

LKHLPEKHMMNSVLENFTVLQVVTNRDTQETLLCIAYVFEVSTSVHGTQHHIYRLV

>Hsa_TEAD4

GRNELIARYIKLRTGKTRTRKQVSSHIQVLARRKSREIQSKLKLQLEFSAFVEPPVDSYQ

RHFVHISPGAPPLESVDVRQIYDKFPEKKGGLRELYDRGPPHAFFLVKFWADLNGPFYGV

SSQYESLEHMTLTCSSKVCSFGKQVVEKVETERAQLEDGRFVYRLLRSPMCEYLVNFLHK

LRQLPERYMMNSVLENFTILQVVTNRDTQELLLCTAYVFEVSTSERGAQHHIYRLV

>Spu_Sd

-RNELIARYIKLRTGKTRTRKQVSSHIQVLARRKARELQALCKLRLEFQAFIERTPDSYN

KHFVHLGFYDPLLEAVDVRQIYDKFPEKKGGLKDLYEKGPAEAFFLVKFWADLNTNFYGV

TSHYESEEKMTITCSTKVCSFGKQVVEKVETEYGRYENGHFIYEISRSPMCEYMINFIHK

LKHLPEKYMMNSVLENFTILQVVTNRDTHETLLCIAFVFEVCTSEHGPQHHIYRLV

>Cin_Sd

GRNELIARYIKLRTGKTRTRKQVSSHIQVLARRKSREMQSQFKLRLDFSAFLEQQLENYS

KHFVHIDYNDPLLESVDIRQIYDKFPEPKGGLRELYEKGPKEAFFLVKFWADLNVNFYAV

TNQFESTENMTITCSTKVCSFGKQVVEKVETEFARFENDRFTYRINRSPMCEYMINFILK

LKHLPEKYMMNSVLENFTILQVISNRDTQETLLCLAFVFEVSTSEHGAQHHIYHLV

>Hma_Sd

GRNELIARYIKLRTGKTRSRKQVSSHIQVLARRK---------LRLDFSAYIDQQPETYH

KHFVHIGFEDPNLEAIDIRQIYDKFPEKKGGLKEFYDKGPQHLFFLVKFWADLNTSFYGV

STTYESSENMTVTCSTKVCSFGKQVVEKVETEYPRYENGRFVYRIQRSPMCEYMINFIRK

LKHLPEKFMMNSVLENFTILQVVSNRDTQETLLCLAFVFEISSSEHGAQHHIFKLV

>Hsa_TEAD5

GRNELIARYIKLRTGKTRTRKQVSSHIQVLARKKVREYQVGIKLRLEYSAFMEVQPDTYS

KHFVHIGFSDPPLEAVDVRQIYDKFPEKKGGLKELYEKGPPNAFFLVKFWADLNSTFYGV

SSQYSSADSMTISVSTKVCSFGKQVVEKVETEYARLENGRFVYRIHRSPMCEYMINFIHK

LKHLPEKYMMNSVLENFTILQVVTSRDSQETLLVIAFVFEVSTSEHGAQHHVYKLV

>Dre_TEAD5b

-RNELIARYIKLRTGKTRTRKQVSSHIQVLARKKMREYQAGIKLRMEYSAFMEVQPDTYS

KHFVHIAYTDPLLEAVDIRQIYDKFPEKKGGLKELYEKGPQNAFFLVKFWADLNSNFYGV

SSQYSSAENMTITVSTKVCSFGKQVVEKVETEYARVEGGRYVYRIHRSPMCEYMINFIHK

LKHLPEKYMMNSVLENFTILQVVTNRDTQETLLCIAFVFEVSTSDHGAQYHVYRLV

>Dre_TEAD5a

GRNELIARYIKLRTGKTRTRKQVSSHLQVLAWRKSREIQSKLKLRMEYSAFMELPQDSYS

KHFVHIGYSDPLLEAVDIRQIYDKFPEKKGGLKELYEKGPQNAFFLVKFWADLNSSFYGV

SSQYSSSENMTITVSTKVCSFGKQVVEKVETEYAHVDGGKCLYRIHRSPMCEYMINFIHK

LKHLPEKYMMNSVLENFTILQVVTNRETQETLLCIAFVFEVSTSEHGAQYHVYRLI

>Lgi_Sd

GRNELIARYIKLRTGKTRTRKQVSSHIQVLARRKSKEIQAQLKLRLEFSAFLEQQPDSYH

KHFVHI-YNDPLLESVDIRQIYDKFPEKKDGLKDLYDKGPQTAFFLVKFWADINTNFYGV

TSQYESSENMTIQVSTKVCSFGKQVVEKVETEYGRFENGRFMYRIHRSPMCEYMINFIHK

LKHLPEKYMMNSVLENFTILQVITNRDTRETLICIAFVFEVSTSEHGAQHHTYKLV

>Xtr_TEAD4

GRNELIARYIKLRTGKTRTRKQVSSHIQVLARRKSREIQSKLKLRLEFLAFMEQQPDAYN

KHYVHISYSDALLESVDVRQIYDKFPEKKGGLKELYEKGPPNAFFLVKFWADLNTSFYGV

SSQYESPENMTITCSSKVCSFGKQVVEKVETEYARFENGRFLYRIHRSPMCEYLINFIHK

LKQLPEKYMMNSVLENFTILQVVTNRDTQELLLCIAYVFEVSTSEHGAQHHIYRLV

>Nve_Sd

GRNELIARYIKLRTGKTRTRKQVSSHLQVLARKKAREIQGKIKLRLEFSAFLEQQPDTYH

KHFVHLGFSDPLLEAVDIRQIYDKFPEKKGGLKDMYDKGPQAVFFLVKFWADLNTNFYGV

SSTYESSENMTITCSTKVCSFGKQVVEKVETEYGHFENGRFVYRIHRSPMCEYMINFIHK

LKQLPEKYMMNSVLENFTILQVVSNRETQETLLCLAYVFEVSTSEHGAQHHIYRLV

>Bfl_Sd

GRNELIARYIKLRTGKTRTRKQVSSHIQVLARRKLRELQSSIKLRLEFSAFMEQQHDTFH

KHFVHIGYSDPLLEQVDIRQIYDKFPEKKGGLKELYDKGPQEAFFLVKFWADLNTNFYGV

TSQYESEQNMTITCSTKVCSFGKQVVEKVETEYARFENGRFVYNIHRSPMCEYMINFIHK

LKHLPEKYMMNSVLENFTILQVVTNRDTQETLLCIAYVFEVSTSEHGAQHHIYRLV

>Xtr_TEAD1

GRNELIARYIKLRTGKTRTRKQVSSHIQVLARKKVREIQAAIKLRLEFSAFLEQQPDAYN

KHFVHIGYSDPLLESVDIRQIYDKFPEKKGGLKELFGKGPQNAFFLVKFWV---------

--------------------RGSQVVGRV-------------------------------

----------------------VTNRDTQETLLCMACVFEVSNSEHGAQHHIYRLV

>DreTEAD1a

GRNELIARYIKLRTGKTRTRKQVSSHIQVLARRKSREFHSKLKLRLEFSAFLEHQPDLYN

KHFVHIGYSDALLETVDIRQIYDKFPEKKGGLKELFGKGPQNAFFLVKFWADLNCNFYGV

TSQYESSENMTITCSTKVCSFGKQVVEKVETEYARFENGRFVYKISRSPMCEYMINFIHK

LKHLPEKYMMDSVLENFTILLVVSNRETQETLLCMACVFEVSNSEHGAQHHIYRLV

>Hsa_TEAD1

GRNELIARYIKLRTGKTRTRKQVSSHIQVLARRKSRDFHSKLKLRLEFSAFLEQQPDSYN

KHFVHIGYSDPLLESVDIRQIYDKFPEKKGGLKELFGKGPQNAFFLVKFWADLNCNFYGV

TSQYESSENMTVTCSTKVCSFGKQVVEKVETEYARFENGRFVYRINRSPMCEYMINFIHK

LKHLPEKYMMNSVLENFTILLVVTNRDTQETLLCMACVFEVSNSEHGAQHHIYRLV

>Dre_TEAD1b

GRNELIARYIKLRTGKTRTRKQVSSHIQVLARRKSRELHSKLKLRLEFSAFLEHQPDSYN

KHFVHIGYNDALLESVDIRQIYDKFPEKKGGLKELFGKGPQNSFFLIKFWADLNCNFYGV

TSQYESPENMTITCSTKVCSFGKQVVEKVETEYARFENGRFVYRISRSPMCEYMINFIHK

LKHLPEKYMMNSVLENFTILLVVTNRDTQETLLCMACVFEVSNSEHGAQHHIYRLV

>Dre_TEAD1c

GRNELIARYIKLRTGKTRTRKQVSSHIQVLARRKSRELHSKLKLRLEFSAFLEHQPDSYN

KHFVHIGYNDALLESVDIRQIYDKFPEKKGGLKELFGKGPQNSFFLIKFWADLNCNFYGV

TSQYESPENMTITCSTKVCSFGKQVVEKVETEYARFENGRFVYRISRSPMCEYMINFIHK

LKHLPEKYMMNSVLENFTILLVVTNRDTQETLLCMACVFEVSNSEHGAQHHIYRLV

>Dre_TEAD3

-------------------------------------------LRLEFSAFLEQPPETVN

KHFVHIAYSDPYLEAVDIRQIYDKFPEKKGGLKELFEKGPTNAFFLVKFWADLSINFYGV

SSQYESSENMIITSSTKVCSFGKQVVEKVETEYARFESGRYVFRIHRSPLCEYMINFIHK

LKHLPEKYMMNSVLENFTILQVITNRDTLETLLCIAYVFEVSTSEHGAQHHIYRLV

>Hsa_TEAD3

GRNELIARYIKLRTGKTRTRKQVSSHIQVLARRKAREIQAKLKLWMEFSAFLEQQPDTYN

KHFVHIGYSDPYLEAVDIRQIYDKFPEKKGGLKDLFERGPSNAFFLVKFWADLNTNFYGV

SSQYESPENMIITCSTKVCSFGKQVVEKVETEYARYENGHYSYRIHRSPLCEYMINFIHK

LKHLPEKYMMNSVLENFTILQVVTNRDTQETLLCIAYVFEVSASEHGAQHHIYRLV

>Ppi_Sd

GRNELIARYIRDKTGKSRTRKQVSSHIQVLARKKSKEIQQHSKLKLD-------------

-------FTDPNLERIDIEKIHDKFPSGGGSLGELFHAGPRQAFFLIKFWVNLNPNFFGH

DNRFESTERLDLKVSTKACSFGKSVVEKI-------TDGRMIYELTKSPLCAYMVTFIYK

LKDLPVPKLMNNVLENFTILQHITDNETGETLLCIACMFEVSTNE------IYRLV

>MleSd

------------------------------------------------------------

-------FSDPNMERIDIEKIHDKFPSGEGSLKELFEAGPSSAFFLVKFWVDLNPNFYGH

DNKYESKEKLHIKVSTKACSFGKGVVEKLQSVSPVAVDGRLIYDVEKSPLCEYMVSFIYK

LKDLPDTKLMNNVLENFTVLQHVTDMETSETLLCVACVFEVSET-------IYRLV
