# Supplementary material for: Comparative study of Hippo pathway genes in cellular conveyor belts of a ctenophore and a cnidarian
Source: EvoDevo. 2016 Feb 19;7:4. doi: 10.1186/s13227-016-0041-y (PMC4761220; doi:10.1186/s13227-016-0041-y)
Supplement: Supplementary file 2 — 10.1186/s13227-016-0041-y Validation of the anti-CheYorkie antibody by Western blot. Total protein extracts from C. hemisphaerica medusae were loaded on three lanes of an electrophoresis gel and then transferred onto a nitrocellulose membranes. After Ponceau red staining, the three lanes were separated for incubation with (lane 1) anti-CheYki antiserum; (lane 2) anti-CheYki antiserum that was previously incubated with the immunising peptide; and (lane 3) pre-immune serum. The results of these Western blot experiments suggest that the anti-CheYki antibody detects the CheYki protein with a satisfactory level of specificity. [file 13227_2016_41_MOESM2_ESM.pdf]

Additional file 2

Validation of the anti-CheYorkie antibody by Western-blot

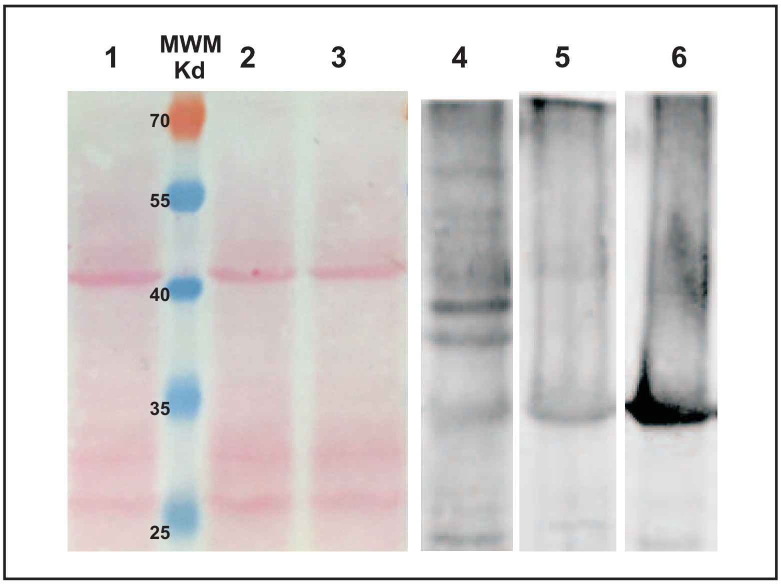

Lanes 1, 2 and 3: Total protein extract from *C. hemisphaerica* medusae (equivalent quantities in lanes 1-2-3), after electrophoresis, transfer onto nitrocellulose membrane, and Ponceau red staining.

The nitrocellulose membrane was then **cut in three pieces** corresponding respectively to lane 1, 2 and 3, for three different treatments:

Lane 4: the nitrocellulose membrane of lane 1 was incubated with **antiserum against CheYorkie** (see Methods for the revelation procedure). The two most intense bands are located around 38 kDa and might represent different isoforms of the CheYorkie protein, whose predicted size (based on the corresponding contig in the transcriptome assembly) is 351 amino-acids, i.e. about 351 x 110 = 38.61 kDa. Other visible bands are much weaker and can be expected in whole-mount immunostaining to generate background without preventing detection of the specific CheYki signal.

Lane 5: the nitrocellulose membrane of lane 2 was incubated with **antiserum against CheYorkie preliminarily incubated with the immunising peptide** (H–CFNRRTTWDDPRKAHS–NH2). The two bands around 38 kDa are absent.

Lane 6: the nitrocellulose membrane of lane 3 was incubated with rabbit **preimmune serum** (negative control). There is no band at 38 kDa. As expected, non specific signal (particularly, a band at 35 kDa) is much more intense with the preimmune serum than with the antiserum.

Conclusion: the antiserum contains antibody(ies) binding a protein that has the predicted size of CheYorkie. Immunoreactivity against this protein is absent with the preimmune serum and is lost when the antiserum is preincubated with the peptide that was used to produce the antiserum.
