# Supplementary material for: Comparative study of Hippo pathway genes in cellular conveyor belts of a ctenophore and a cnidarian
Source: EvoDevo. 2016 Feb 19;7:4. doi: 10.1186/s13227-016-0041-y (PMC4761220; doi:10.1186/s13227-016-0041-y)
Supplement: Supplementary file 3 — 10.1186/s13227-016-0041-y Negative controls of whole-mount immunolocalisation experiments. C. hemisphaerica medusae incubated with pre-immune serum and with secondary antibody are compared with medusae incubated with the anti-CheYki antibody (in the same experiment). The negative controls show background signal in the endoderm of the tentacle bulb but no signal in the ectoderm. [file 13227_2016_41_MOESM3_ESM.pdf]

Additional file 3

Negative controls of whole-mount immunofluorescent labelling using preimmune serum and secondary antibody

All pictures show a tentacle bulb with its tentacle, together with a portion of the medusa bell periphery (focus on subumbrellar epidermis).

All pictures shown below are from one and the same experiment, and the same exposure time was used for the three conditions.

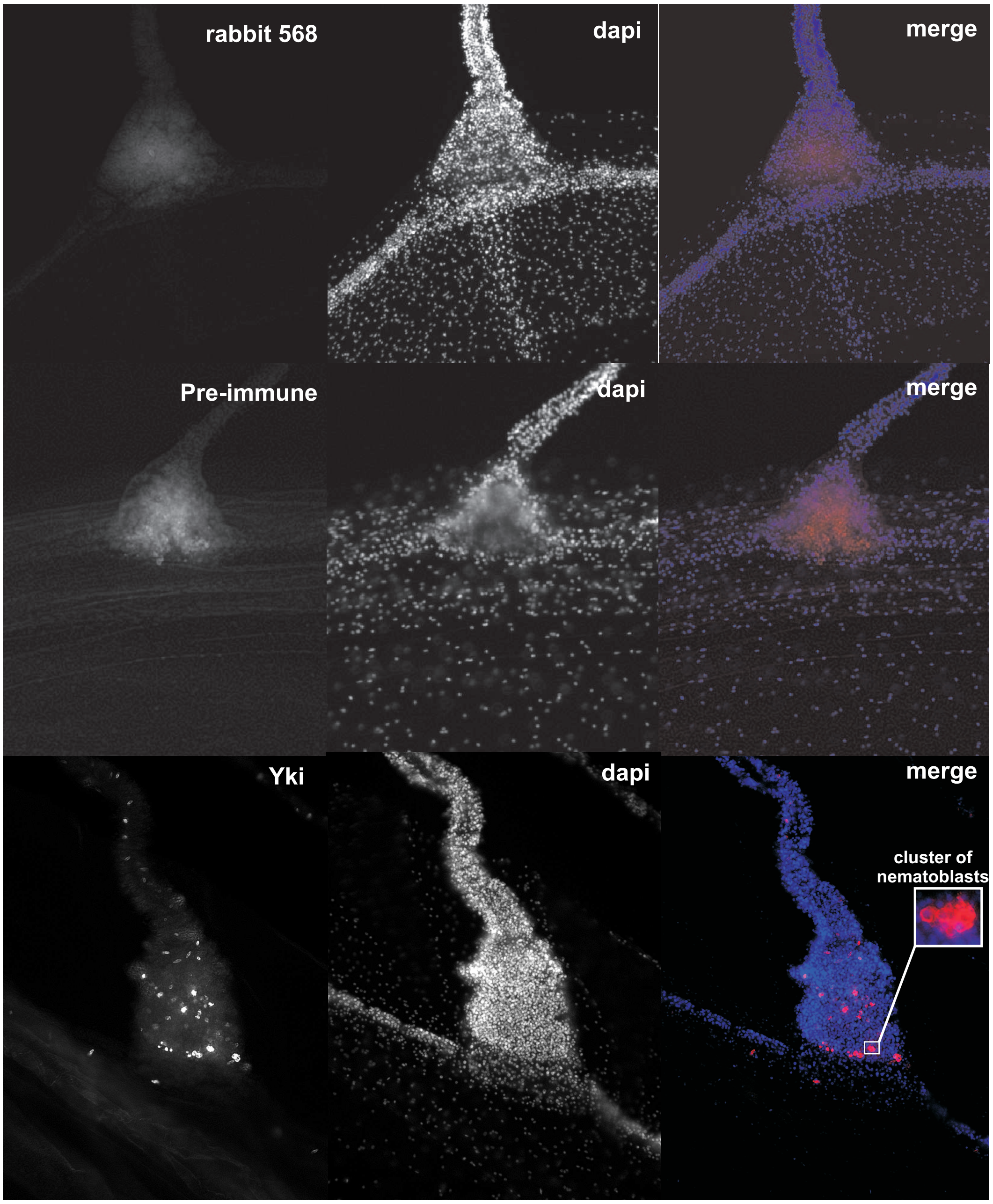

Conclusion

The secondary (anti-rabbit 568) antibody and the pre-immune serum give respectively weak and stronger non-specific staining in the tentacle bulb **endoderm** but **no signal in the ectoderm** of the bulb and of the mature tentacle, and neither in the umbrella.
