# Supplementary material for: Comparative study of Hippo pathway genes in cellular conveyor belts of a ctenophore and a cnidarian
Source: EvoDevo. 2016 Feb 19;7:4. doi: 10.1186/s13227-016-0041-y (PMC4761220; doi:10.1186/s13227-016-0041-y)

Comparative study of Hippo pathway genes in cellular conveyor belts of a ctenophore and a cnidarian

A. Coste, M. Jager, J.-P. Chambon and M. Manuel

Additional file 4

Phylogenetic analyses of core Hippo pathway genes (other than Yorkie)

page	Gene family
2	Cyclins
3	*comment on cyclins tree*
4	Salvador (WW domain only)
5	Salvador (WW + SARAH domains)
6	Hippo
7	Warts
8	Mats
9	Scalloped

Cyclins

(65 amino-acids)

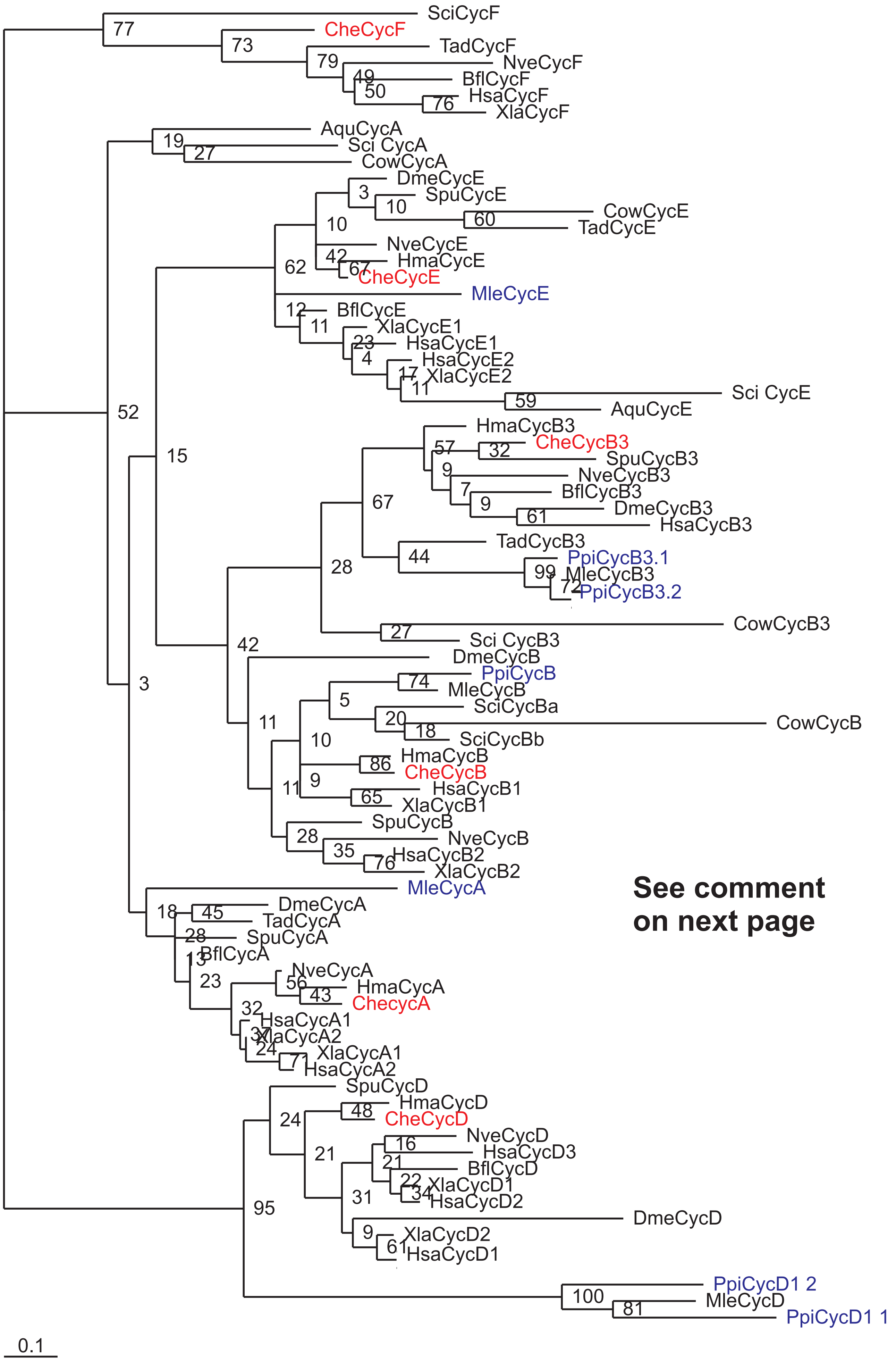

See comment on next page

Comment concerning the tree of cyclins

The cyclin subfamilies are strongly conserved at the metazoan level and unsurprisingly, we could detect orthologues of cyclin A, B, B3, D and E in all sampled metazoans including ctenophores. More interestingly, *P. pileus* has two paralogues of cyclin B3 and two paralogues of cyclin D, whereas the other ctenophore *M. leydii* has only one for both subfamilies. In *P. bachei* we found two paralogues of cyclin B3 but only one cyclin D (not shown). In both cases, the tree topology suggests secondary loss of one of the duplicated ctenophore cyclins in *M. leydii*.

Salvador

Alignment of WW domains only (34 amino-acids)

* proteins which contain a SARAH domain (the Hippo-interacting domain of Sav)

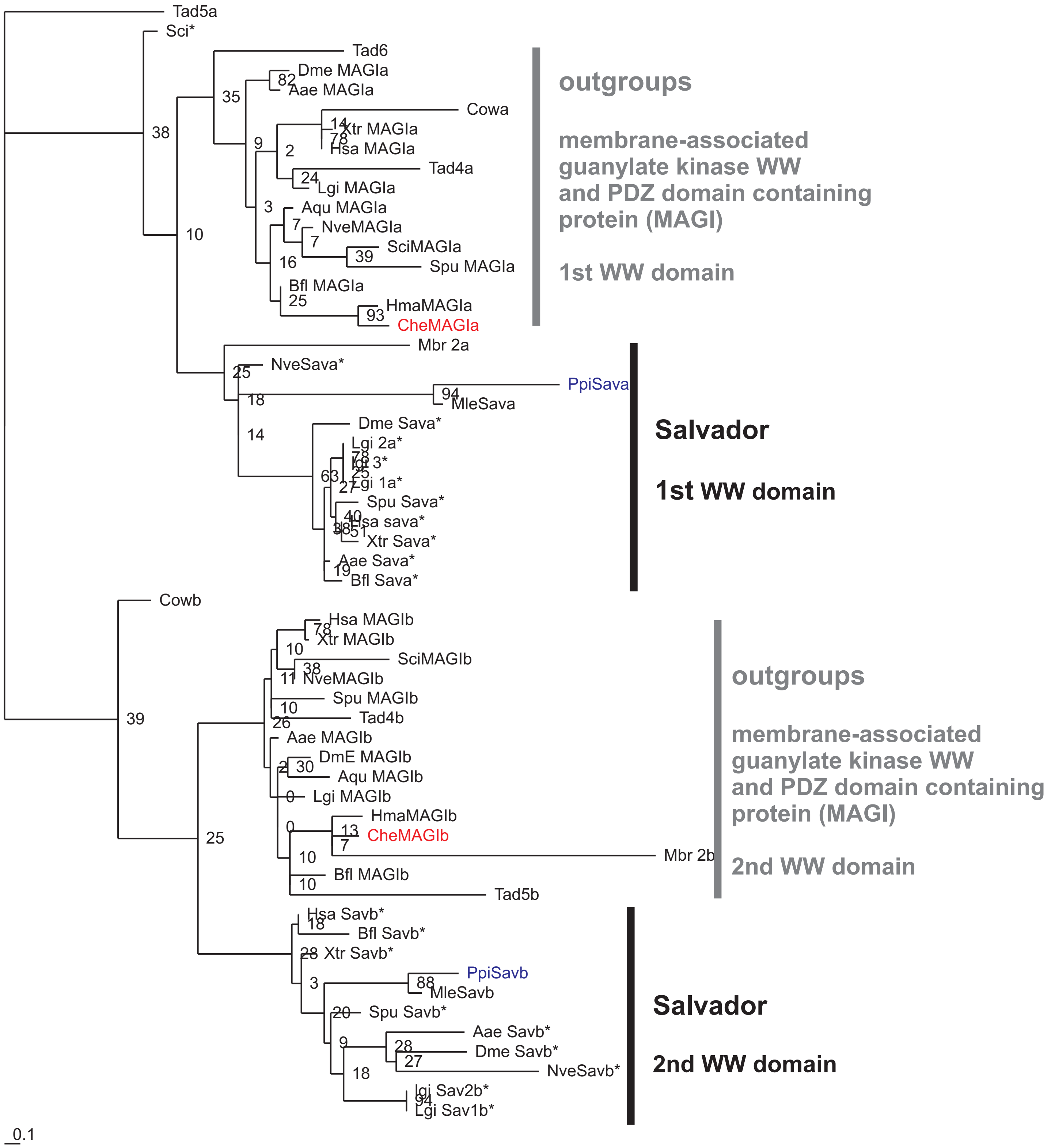

Note: WW domains are absent in the Salvador proteins of hydrozoans (*Clytia hemisphaerica* and *Hydra magnipapillata*).

Salvador

Combined analysis of the two WW domains and the SARAH domain
(116 amino-acids)

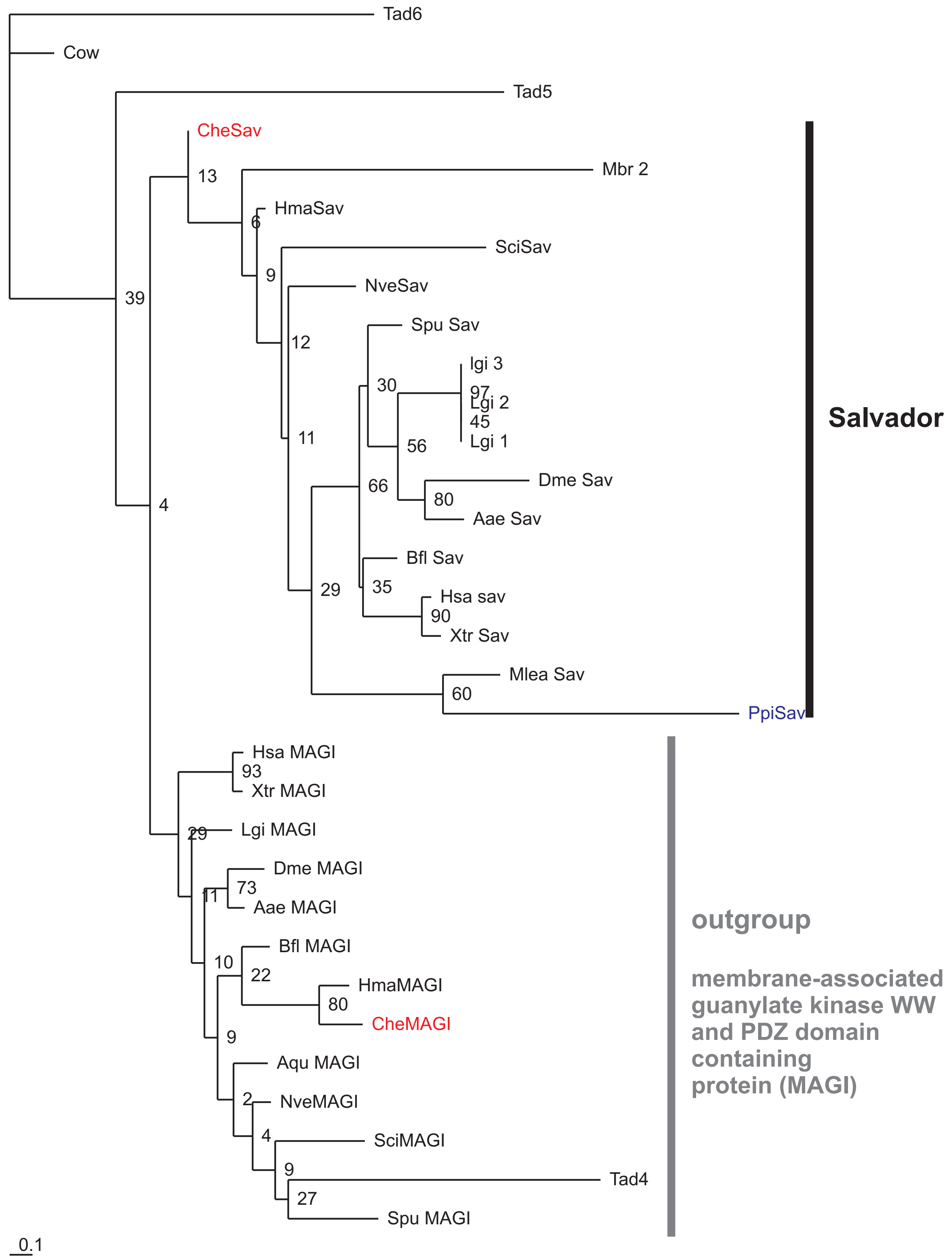

Note: WW domains are absent in the Salvador proteins of hydrozoans (*Clytia hemisphaerica* and *Hydra magnipapillata*). In this alignment they have been replaced by missing data.

Hippo

(234 amino-acids)

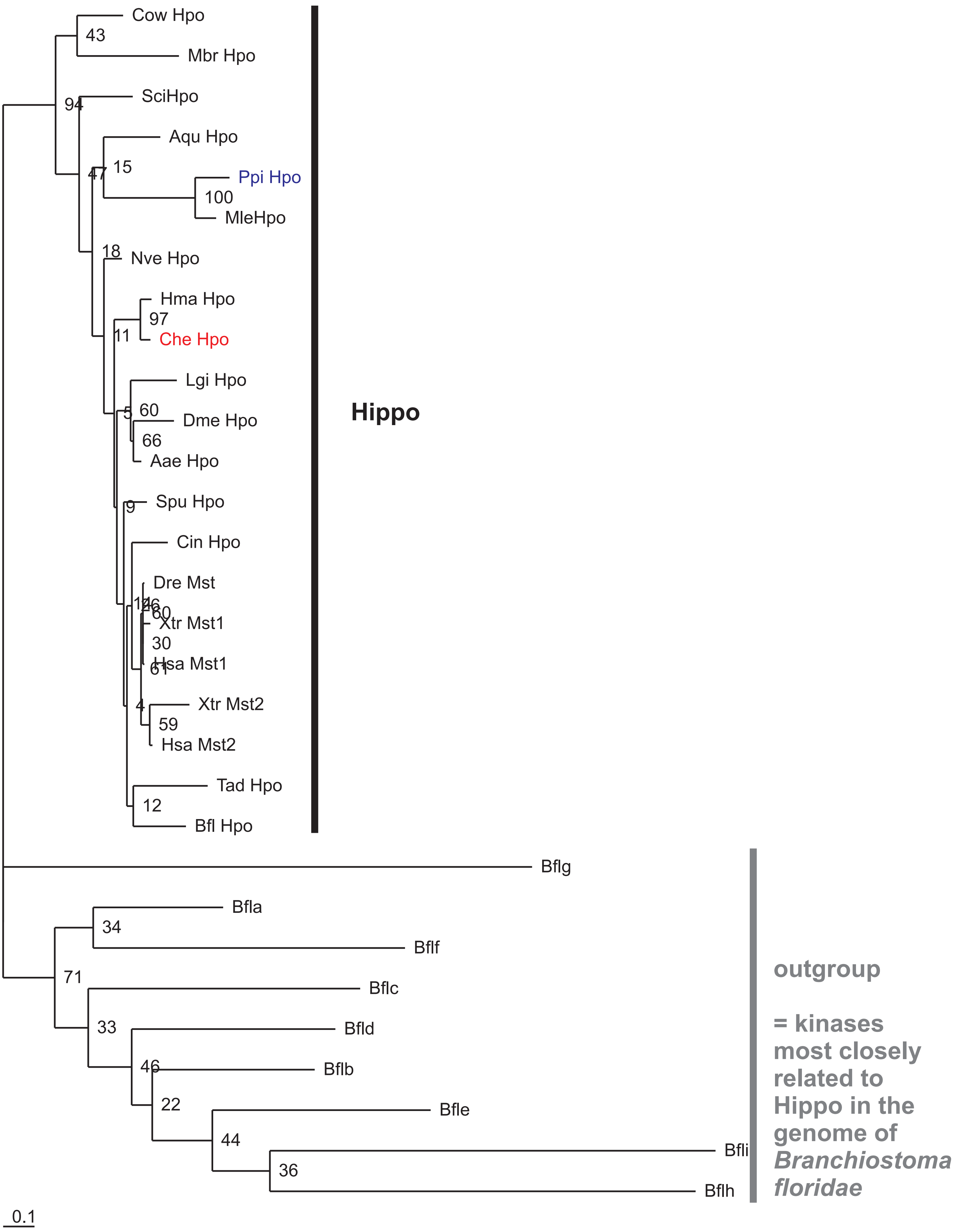

Warts

(325 amino-acids)

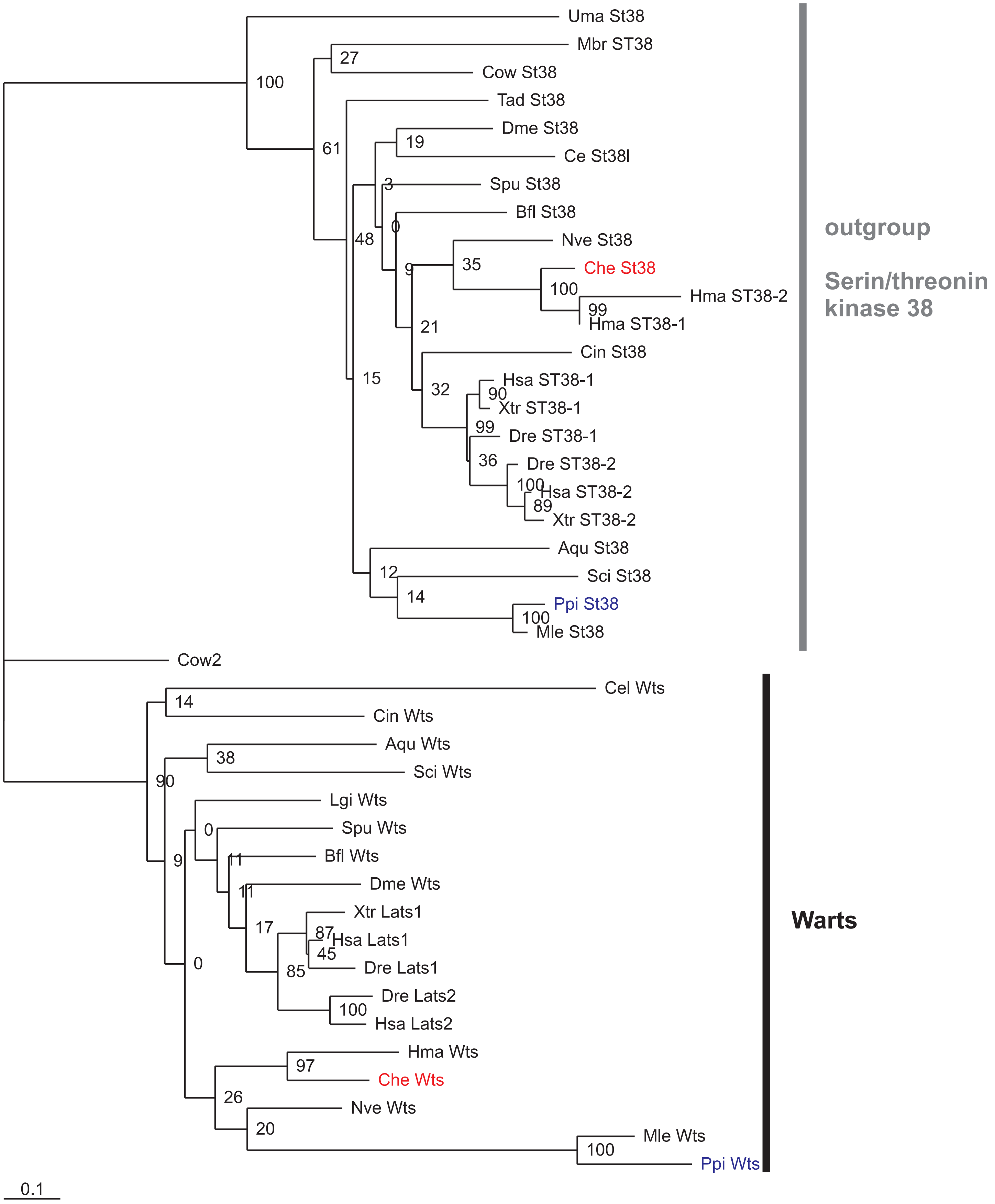

Mats

(182 amino-acids)

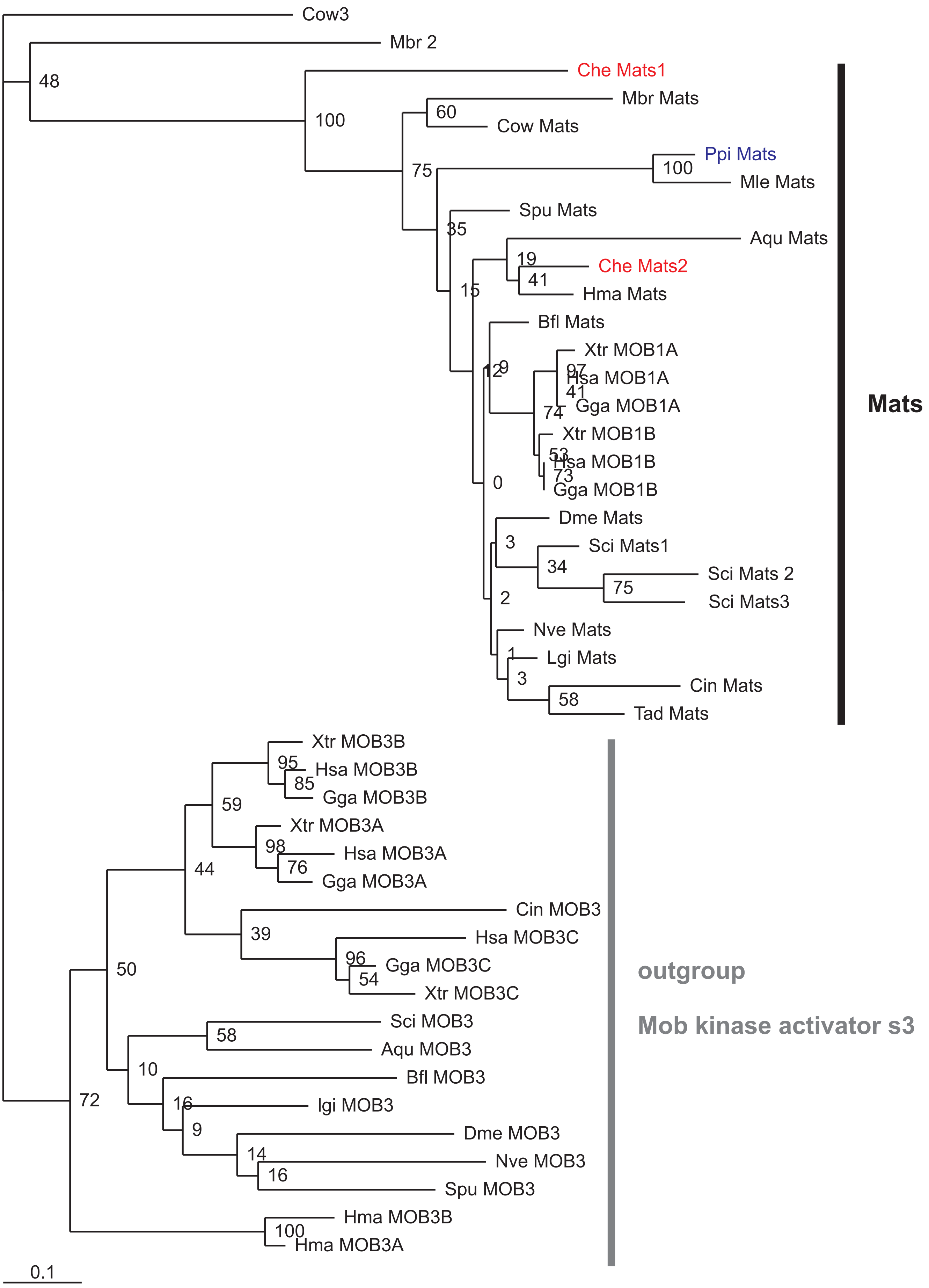

Scalloped

(240 amino-acids)

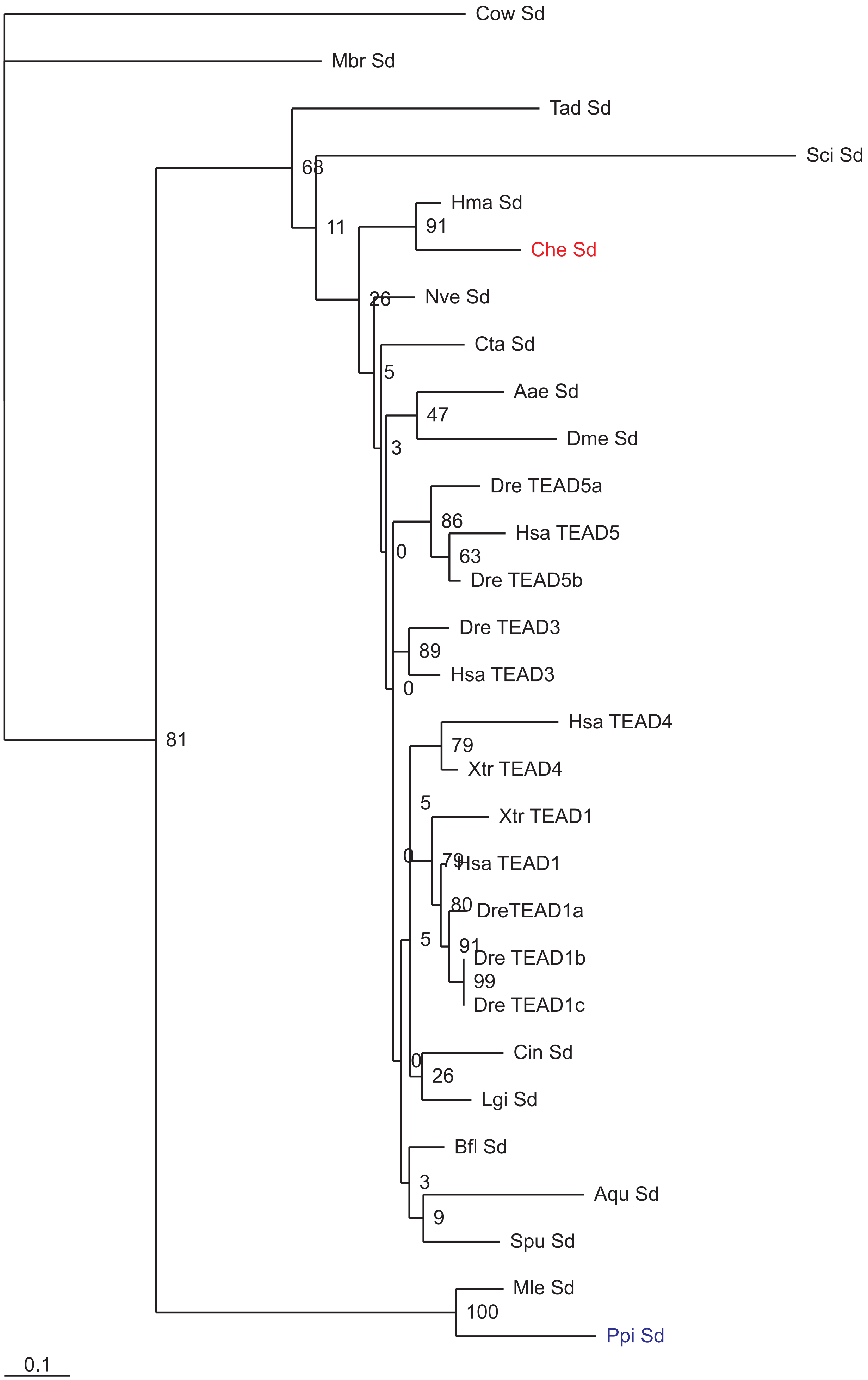

Supplement: Supplementary file 4 — 10.1186/s13227-016-0041-y Phylogenetic analyses of Cyclins and core Hippo pathway genes (other than Yorkie). Maximum likelihood trees are shown for cyclins (page 2; with comment on page 3), Sav WW domains (page 4), Sav combined WW and SARAH domains (page 5), Hippo (page 6), Warts (page 7), Mats (page 8) and Sd (page 9). [file 13227_2016_41_MOESM4_ESM.pdf]
