# Supplementary material for: Comparative study of Hippo pathway genes in cellular conveyor belts of a ctenophore and a cnidarian
Source: EvoDevo. 2016 Feb 19;7:4. doi: 10.1186/s13227-016-0041-y (PMC4761220; doi:10.1186/s13227-016-0041-y)
Supplement: Supplementary file 5 — 10.1186/s13227-016-0041-y Results of PCR amplification and sequencing of a part of the Mle Yki - like genomic locus. Graphical representation of our PCR amplification and sequencing of the 5′ part of the Mle Yki-like genomic locus from Mnemiopsis leidyi genomic DNA. The results confirm the domain structure of the gene as predicted from the genome sequence (with one TBD and two WW domains). [file 13227_2016_41_MOESM5_ESM.pdf]

Comparative study of Hippo pathway genes in cellular conveyor belts of a ctenophore and a cnidarian
A. Coste, M. Jager, J.-P. Chambon and M. Manuel

Additional file 5

Validation of the ***Yk-like*** *Mnemiopsis leidyi* gene model by PCR on genomic DNA and sequencing

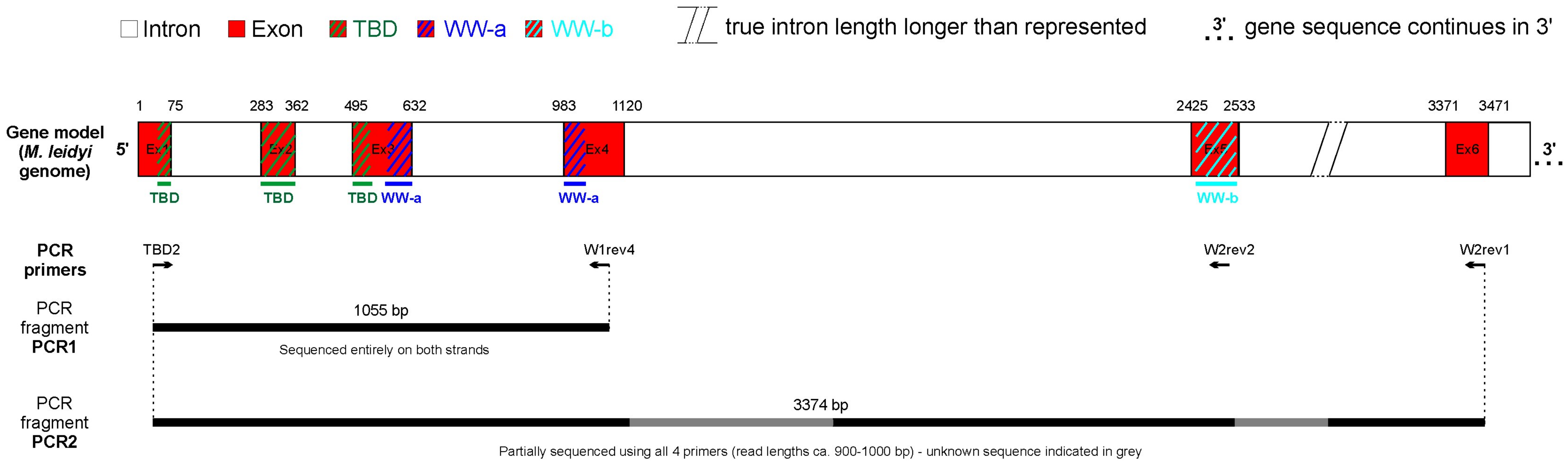

Comments

Genomic DNA was extracted from a specimen of *Mnemiopsis leidyi* collected in Villefranche-sur-Mer (France). Two PCR fragments were amplified as indicated on the figure (primers aligned with the gene model). Assembled sequences were found to align perfectly with the corresponding regions of the gene model from the *M. leidyi* genome. As expected, there are local polymorphisms between the nucleotidic sequences produced here and that of the published genome, but the translated exons are 100% identical in amino-acid sequences.
The presence in this gene of a TBD domain and two WW domains (in this order) is confirmed.

PCR primer sequences

TBD2	5' TACAGTATTCACATCAGAGGC 3'
W1rev4	5'GAAACAACGTAGTTGCTGTGC 3'
W2rev2	5'CTGCTTCCATCCCACAGGCAG 3'
W2rev1	5'GTTCTGCAGAGGAATTATCGG 3'
