# Supplementary material for: Comparative study of Hippo pathway genes in cellular conveyor belts of a ctenophore and a cnidarian
Source: EvoDevo. 2016 Feb 19;7:4. doi: 10.1186/s13227-016-0041-y (PMC4761220; doi:10.1186/s13227-016-0041-y)
Supplement: Supplementary file 6 — 10.1186/s13227-016-0041-y Negative controls of ISH experiments. The pictures show absence of colorimetric signal in C. hemisphaerica and P. pileus specimens when the hybridisation step of the ISH protocol was performed with either no probe or with a sense RNA probe. [file 13227_2016_41_MOESM6_ESM.pdf]

Comparative study of Hippo pathway genes in cellular conveyor belts of a ctenophore and a cnidarian

A. Coste, M. Jager, J.-P. Chambon and M. Manuel

Additional file 6

Negative controls of *in situ* hybridisation experiments

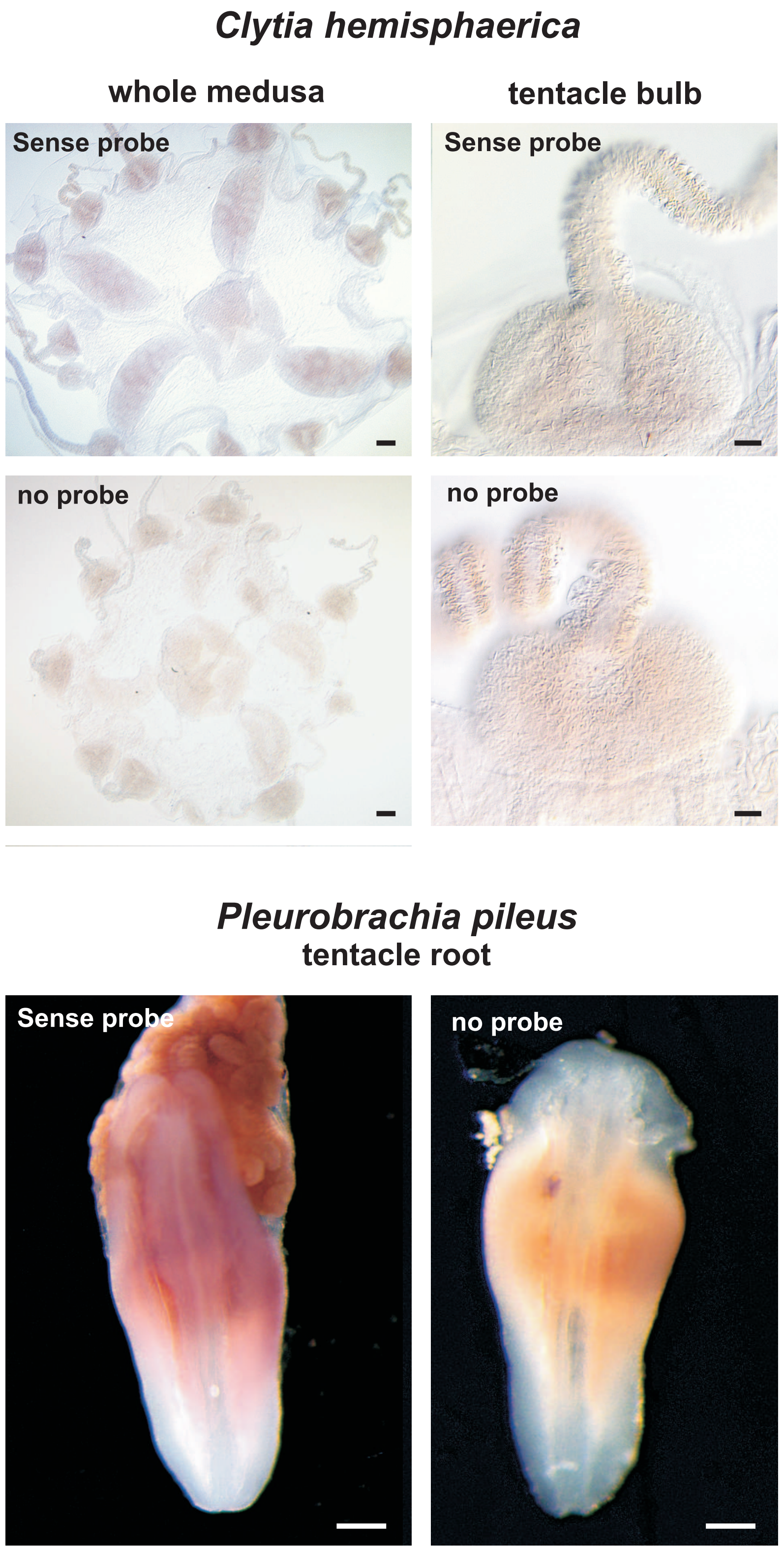

All scale bars: 100 µm
