# Supplementary material for: Comparative study of Hippo pathway genes in cellular conveyor belts of a ctenophore and a cnidarian
Source: EvoDevo. 2016 Feb 19;7:4. doi: 10.1186/s13227-016-0041-y (PMC4761220; doi:10.1186/s13227-016-0041-y)
Supplement: Supplementary file 7 — 10.1186/s13227-016-0041-y Details of PpiHpo expression in the tentacular apparatus. The pictures show that although PpiHpo is turned off in the differentiation zone of the colloblasts in the lateral cellular conveyor belts of the tentacle root (cf. Figure 3h), it is later re-expressed at a strong level in the epithelium of the tentacle axis and tentillae, starting from a point at a short distance from tentacle basis. [file 13227_2016_41_MOESM7_ESM.pdf]

**Comparative study of Hippo pathway genes in cellular conveyor belts of a ctenophore and a cnidarian**

A. Coste, M. Jager, J.-P. Chambon and M. Manuel

## Additional file 7

Details about Hippo expression in the *Pleurobrachia pileus* tentacle

A

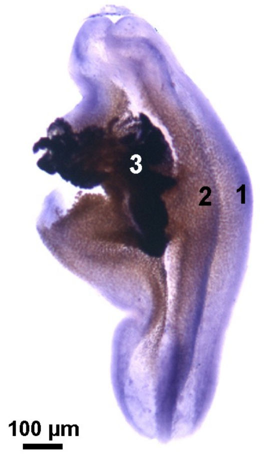


**Expression characteristics of *PpiHpo* in the tentacle root**

This picture of a whole-mount tentacle root in external view is complementary to Fig. 3G (whole mount, internal view) and Fig. 3H (transverse section).
In the colloblast cellular conveyor belt, *PpiHpo* is expressed in the stem cell / proliferation zone (1) (= lateral ridges), is not expressed in the differentiated colloblasts on the surface of the tentacle root (2) but is strongly expressed in the tentacle and tentillae (3).

B

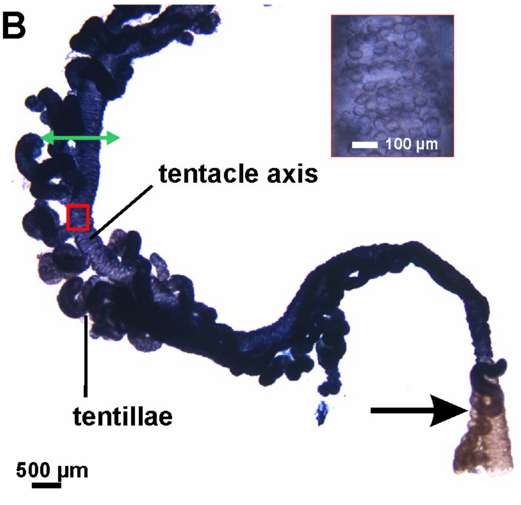


**Expression of *PpiHpo* along the tentacle**

This picture shows an entire tentacle that has been cut at its insertion point on the tentacle root.
*PpiHpo* is not expressed in the most proximal part (below arrow) but is strongly expressed in the rest (above arrow), both in the tentacle axis and its lateral diverticulae (tentillae).
Inset: detail of the aspect of the staining at the surface of the tentacle axis (red box on left picture).

C

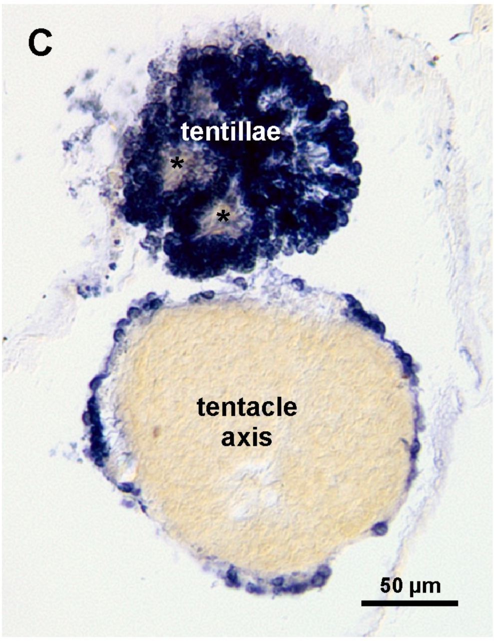


**Aspect of the *PpiHpo* staining in transverse cryosection of the tentacular apparatus** (approximately at the level of green double arrow in B)

The staining is superficial in the tentacle axis. *PpiHpo* is expressed not only in colloblasts (round cells), but also in other (flatter) epithelial cells.

In tentillae, the thick colloblast layer is intensely stained whereas the central part (asterisks) is unstained.
