# Supplementary material for: Comparative study of Hippo pathway genes in cellular conveyor belts of a ctenophore and a cnidarian
Source: EvoDevo. 2016 Feb 19;7:4. doi: 10.1186/s13227-016-0041-y (PMC4761220; doi:10.1186/s13227-016-0041-y)
Supplement: Supplementary file 8 — 10.1186/s13227-016-0041-y In situ hybridisation for CheHpo, CheYk, CheSav and CheSd in whole medusae. These pictures show ubiquitous expression of CheHpo, CheYk, CheSav and CheSd in the C. hemisphaerica medusa. Scale bars: 100 µm. [file 13227_2016_41_MOESM8_ESM.pdf]

Comparative study of Hippo pathway genes in cellular conveyor belts of a ctenophore and a cnidarian

A. Coste, M. Jager, J.-P. Chambon and M. Manuel

Additional file 8

In situ hybridisation for *CheHpo, CheYk, CheSav* and *CheSd* in whole medusae

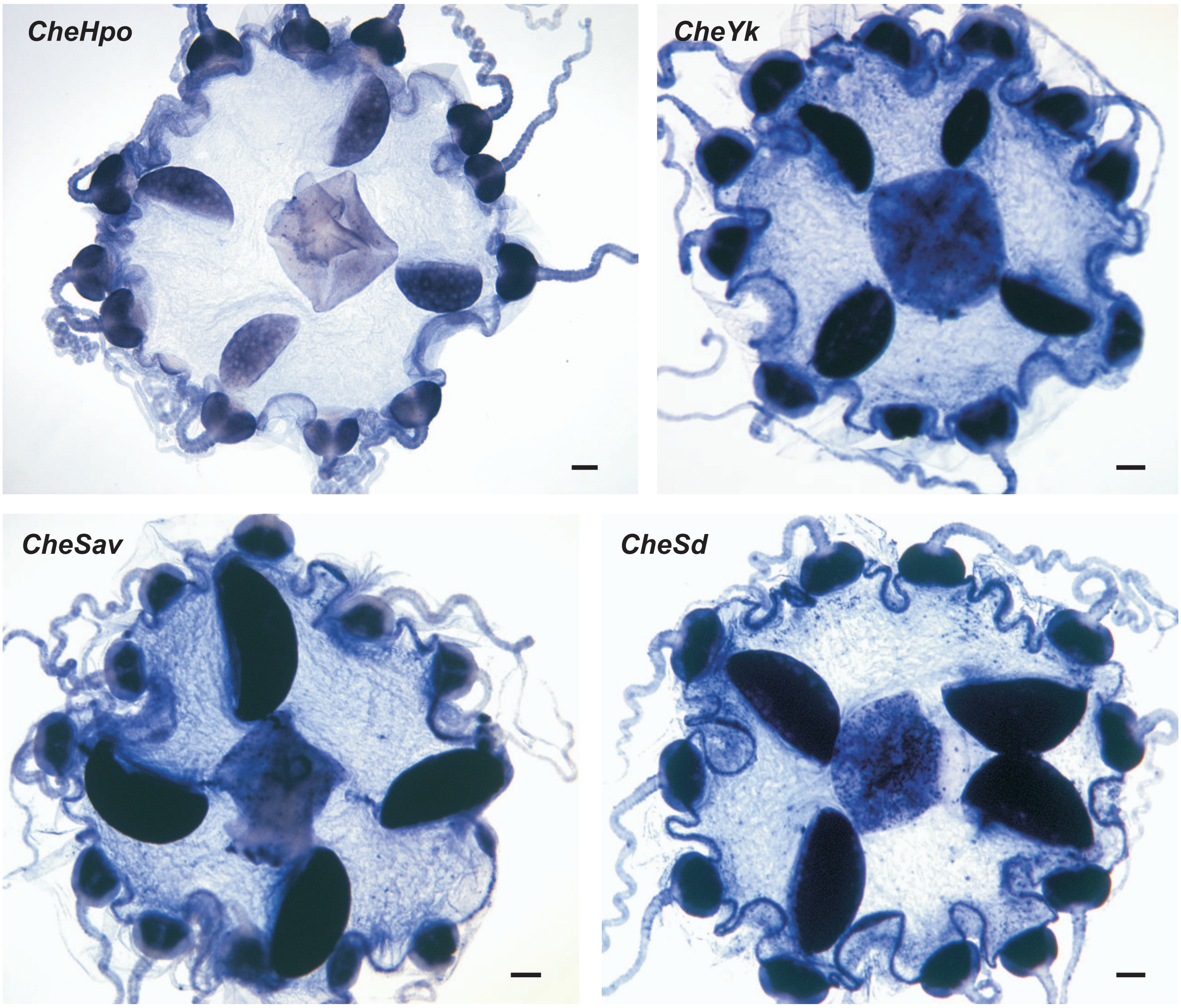

Scale bars: 100 µm
