# Supplementary material for: Comparative study of Hippo pathway genes in cellular conveyor belts of a ctenophore and a cnidarian
Source: EvoDevo. 2016 Feb 19;7:4. doi: 10.1186/s13227-016-0041-y (PMC4761220; doi:10.1186/s13227-016-0041-y)
Supplement: Supplementary file 9 — 10.1186/s13227-016-0041-y Anti-Yorkie immunoreactivity in the C. hemisphaerica subumbrellar epidermis (A) and in the manubrium (B). In (9A), the three pictures are focused on the subumbrella. rad can: radial canal; tent: tentacle (superimposed). (1) and (2) are conventional epifluorescence microscopy views; (3) is a confocal section. (9B) shows a confocal section in the manubrium epidermis, with graphs of Dapi and anti-Yorkie immunofluorescence along the four coloured arrows on the picture. [file 13227_2016_41_MOESM9_ESM.pdf]

Comparative study of Hippo pathway genes in cellular conveyor belts of a ctenophore and a cnidarian

A. Coste, M. Jager, J.-P. Chambon and M. Manuel

Additional file 9A

Anti-Yorkie immunoreactivity in the *C. hemisphaerica* sub-umbrellar epidermis

rad can: radial canal; tent: tentacle.

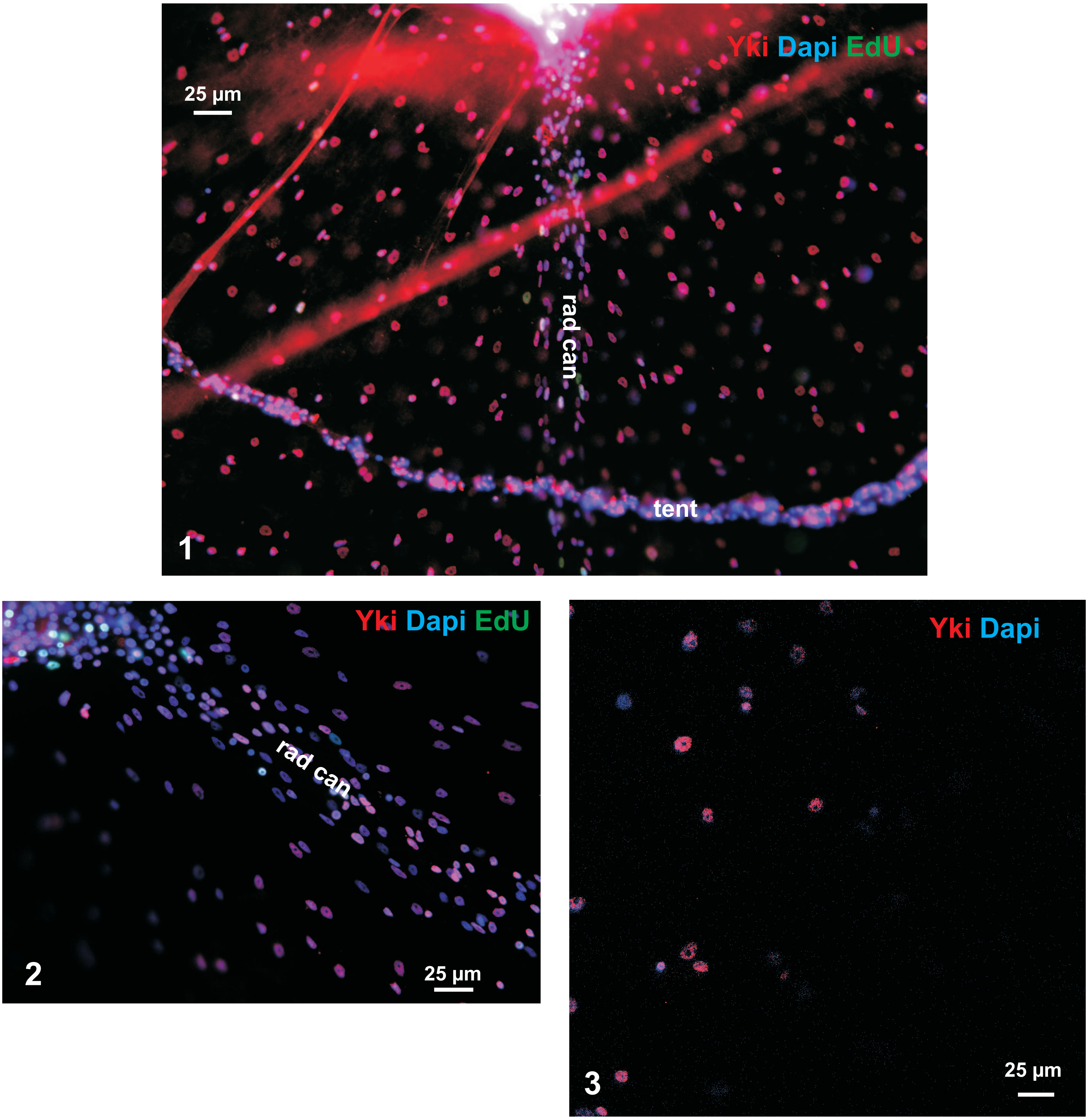

Additional file 9B

Anti-Yorkie immunoreactivity in the *C. hemisphaerica* manubrium

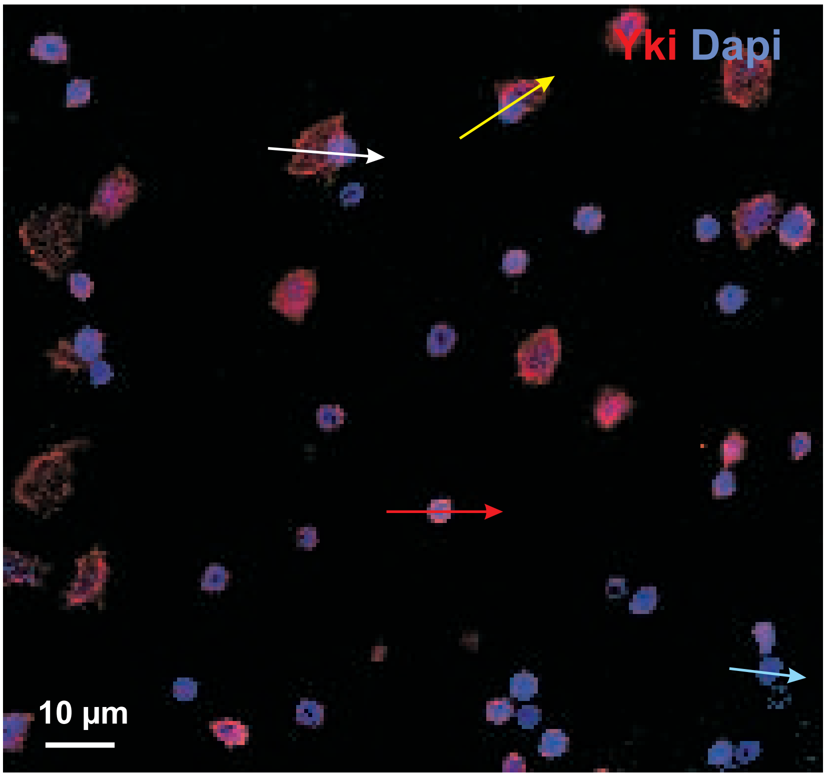

anti-Yorkie

Dapi

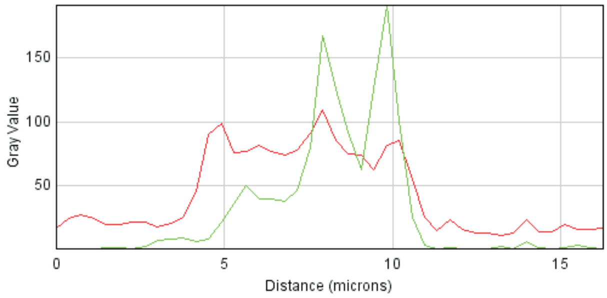

White arrow:
Yorkie in cytoplasm and nucleus

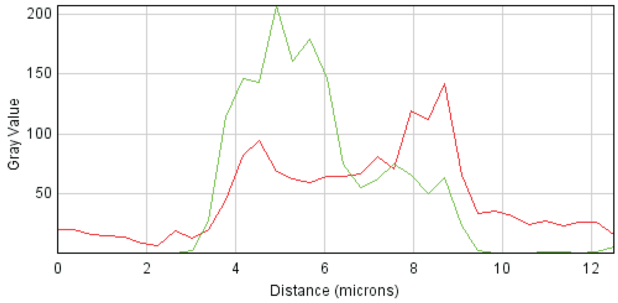

Yellow arrow:
Yorkie in cytoplasm and nucleus

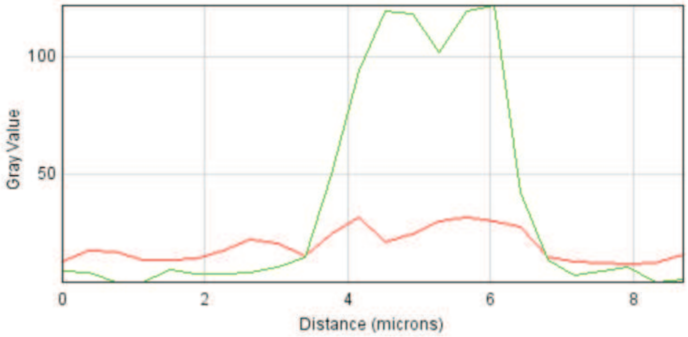

Blue arrow:
Cell without anti-Yorkie signal

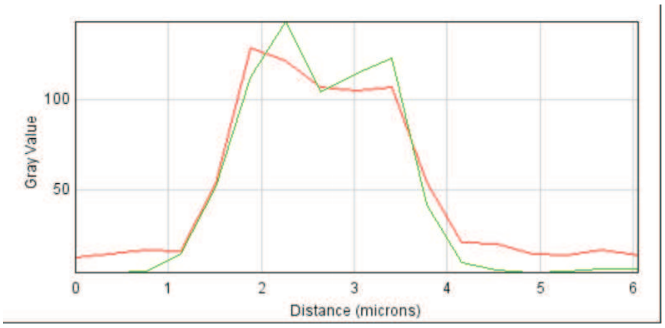

Red arrow:
Yorkie only in nucleus
