# Supplementary material for: Comparative study of Hippo pathway genes in cellular conveyor belts of a ctenophore and a cnidarian
Source: EvoDevo. 2016 Feb 19;7:4. doi: 10.1186/s13227-016-0041-y (PMC4761220; doi:10.1186/s13227-016-0041-y)

Comparative study of Hippo pathway genes in cellular conveyor belts of a ctenophore and a cnidarian

A. Coste, M. Jager, J.-P. Chambon and M. Manuel

Additional file 10

Identification of the ovoid anti-Yki stained structures in proximal half of the tentacle bulb as capsules (nematocysts) of nematoblasts undergoing differentiation

A - Image of anti-Yki stained capsules

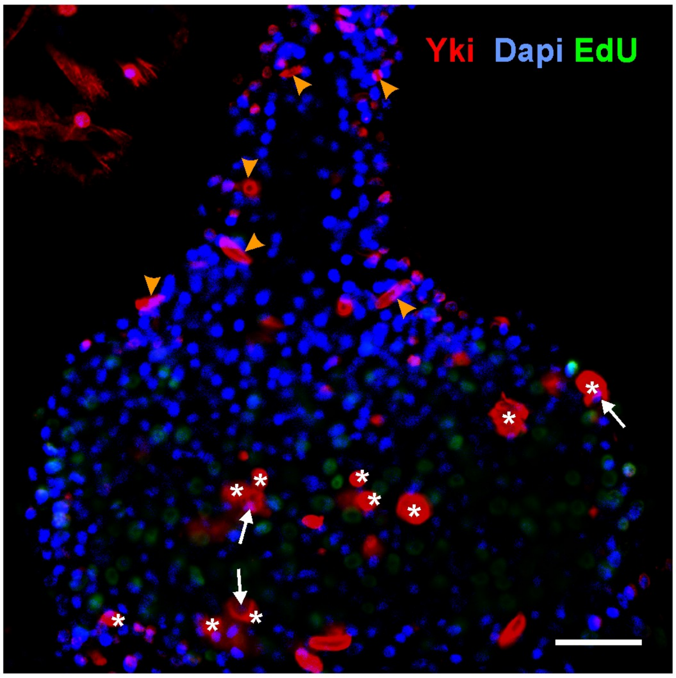

In this composite picture, with respect to those of Fig. 6, the red (Yki) signal has been considerably weakened (to reduce saturation around the capsules), the blue (Dapi) signal has been enhanced, and the green (EdU) toned down. Capsules of undifferentiated nematoblasts (recognisable as such by their size and ovoid-irregular shape) are indicated by **white asterisks** and the corresponding cell nuclei by **white arrows**; examples of differentiated capsules of mature nematocytes are indicated by **orange arrowheads**.
Scale bar: 20 µm.

B- Visualisation of nematoblast and nematocyte capsules using a classical marker: anti-glutamate antibody

White arrowheads: capsules of undifferentiated nematoblasts.

White double arrowheads: capsules of mature nematocytes.

By comparison with the picture shown in A, these observations confirm that the cytoplasmic Yki staining in tentacle bulb ectoderm corresponds to capsules (= nematocysts) of nematoblasts and of mature nematocytes.

Scale bars: 50 µm

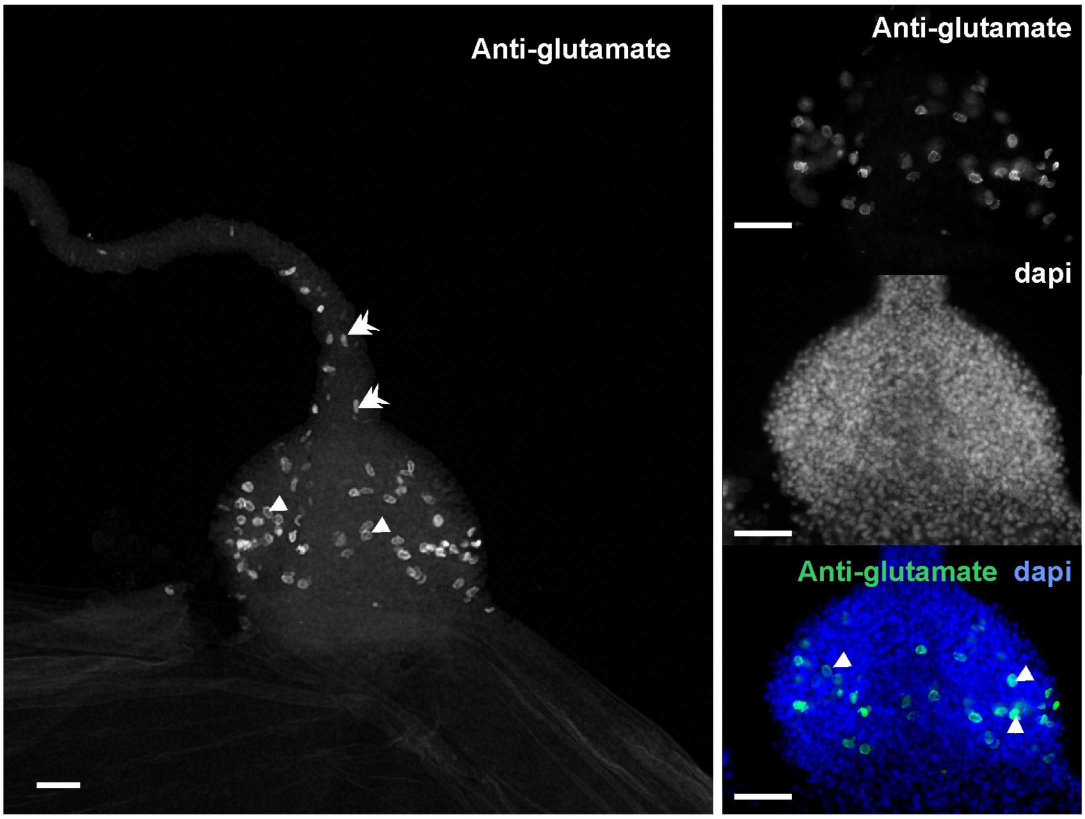

Supplement: Supplementary file 10 — 10.1186/s13227-016-0041-y Additional details about anti-Yki-stained capsules of nematoblasts and nematocysts. The upper picture is a confocal section of a tentacle bulb similar to that of Fig. 6b, but with red decreased and blue increased in order to de-saturate the Yki signal in nematoblast capsules and to allow visualisation of associated nuclei. The lower panel shows the aspect of capsules stained with an anti-glutamate antibody. [file 13227_2016_41_MOESM10_ESM.pdf]
