# Supplementary material for: Comparative study of Hippo pathway genes in cellular conveyor belts of a ctenophore and a cnidarian
Source: EvoDevo. 2016 Feb 19;7:4. doi: 10.1186/s13227-016-0041-y (PMC4761220; doi:10.1186/s13227-016-0041-y)
Supplement: Supplementary file 12 — 10.1186/s13227-016-0041-y Counts of anti-Yki cellular profiles in the exumbrellar epidermis. In this table are given the numbers that were used to build the graph of Fig. 8b. [file 13227_2016_41_MOESM12_ESM.pdf]

Comparative study of Hippo pathway genes in cellular conveyor belts of a ctenophore and a cnidarian

A. Coste, M. Jager, J.-P. Chambon and M. Manuel

Additional file 12

Quantification of nucleus profiles in the exumbrellar epidermis

See localisation of the areas on Fig. 8A and graphical presentation of these results in Fig. 8B.
Pictures of immunostaining in the exumbrellar epidermis are presented in Fig. 7.

		no Yki	Yki in nucleus only	Yki in nucleus and cytoplasm	Yki in cytoplasm only	total
**Central area**	**total**	**31**	**60**	**143**	**48**	**282**
	EdU in nucleus	0	3	2	0	
	no EdU	31	57	141	48	
**Peripheral area**	**total**	**3**	**286**	**9**	**0**	**298**
	EdU in nucleus	0	84	4	0	
	no EdU	3	202	5	0	
